# Accurate identification and discrimination of *Salmonella enterica* serovar Gallinarum biovars Gallinarum and Pullorum by a multiplex PCR based on the new genes of *torT* and *I137_14430*

**DOI:** 10.3389/fvets.2023.1220118

**Published:** 2023-07-05

**Authors:** Li Song, Ruimeng Tan, Dan Xiong, Xinan Jiao, Zhiming Pan

**Affiliations:** ^1^Jiangsu Key Laboratory of Zoonosis, Yangzhou University, Yangzhou, China; ^2^Jiangsu Co-innovation Center for Prevention and Control of Important Animal Infectious Diseases and Zoonoses, Yangzhou University, Yangzhou, China; ^3^Key Laboratory of Prevention and Control of Biological Hazard Factors (Animal Origin) for Agrifood Safety and Quality, Ministry of Agriculture and Rural Affairs, Yangzhou University, Yangzhou, China

**Keywords:** *Salmonella* Pullorum, *Salmonella* Gallinarum, multiplex PCR, *tor*T, *I137_14430*, accurate discrimination

## Abstract

Most cases of chicken salmonellosis are caused by *Salmonella enterica* serovar Gallinarum biovars Gallinarum and Pullorum, which lead to a significant morbidity and fatality rate. Although the conventional Kaufmann-White scheme is the reliable method for the serotyping of *Salmonella*, it does not distinguish between closely related biotypes like *S*. Pullorum and *S*. Gallinarum. Herein, we conducted a single one-step multiplex PCR assay that can identify and distinguish between *S*. Pullorum and *S*. Gallinarum in an accurate manner. This PCR method was based on three genes, including *torT* for *S*. Pullorum identification, *I137_14430* for *S*. Gallinarum identification, and *stn* as the genus-level reference gene for *Salmonella*. By comparing *S*. Pullorum to *S*. Gallinarum and other serovars of *Salmonella, in silico* study revealed that only the former has a deletion of 126 bp-region in the carboxyl terminus of *torT*. The *I137_14430* gene does not exist in *S*. Gallinarum. However, it is present in all other *Salmonella* serotypes. The multiplex PCR approach utilizes unique sets of primers that are intended to specifically target these three different genes. The established PCR method was capable of distinguishing between the biovars Pullorum and Gallinarum from the 29 distinct *Salmonella* serotypes as well as the 50 distinct pathogens that are not *Salmonella*, showing excellent specificity and exclusivity. The minimal amount of bacterial cells required for PCR detection was 100 CFU, while the lowest level of genomic DNA required was 27.5 pg/μL for both *S*. Pullorum and *S*. Gallinarum. After being implemented on the clinical *Salmonella* isolates collected from a poultry farm, the PCR test was capable of distinguishing the two biovars Pullorum and Gallinarum from the other *Salmonella* strains. The findings of the PCR assay were in line with those of the traditional serotyping and biochemical identification methods. This new multiplex PCR could be used as a novel tool to reinforce the clinical diagnosis and differentiation of *S*. Pullorum and *S*. Gallinarum, particularly in high-throughput screening situations, providing the opportunity for early screening of infections and, as a result, more effective management of the illness among flocks.

## 1. Introduction

*Salmonella enterica* is one of the most significant food-borne pathogens. Based on the White-Kauffmann-Le Minor method, over 2,650 distinct serotypes of *Salmonella* have been identified by their distinctive combinations of somatic (O) and flagellar (H) antigens (1, 2). The infection of *Salmonella enterica*, like *S*. Enteritidis, *S*. Typhimurium, *S*. Infantis and *S*. Kentucky, in poultry and poultry products is on the rise (3, 4). Fowls are the specific host of *Salmonella enterica* serovar Gallinarum biovars Pullorum and Gallinarum. *S*. Pullorum and S. Gallinarum are currently regarded as biovars of serovar Gallinarum within serogroup D, which cause pullorum disease (PD) and fowl typhoid (FT), respectively (5). *S*. Pullorum can be transmitted vertically to newborn hatchlings and horizontally to other birds and cause serious economic burden for the poultry industry (6).

Chicks aged < 3 weeks are most susceptible to contracting Pullorum disease, which is a systemic disease. White viscous diarrhea and acute septicemia are hallmarks of Pullorum disease, which is associated with a high rate of morbidity and mortality (reaching 100% among young chicks) (7). Symptoms of infections in adult birds include malnutrition, reduced egg production, diarrhea, reproductive system deformities, and more (8, 9). Animals that survive may become carriers, may not meet expected animal production requirements, and could produce contaminated eggs (10). In poultry, *S*. Gallinarum most often results in fowl typhoid, which may manifest as either chronic or acute septicemia in young birds as well as adult birds (11). The diseases continue to be of significant economic burden to the poultry business in several nations throughout South America, Central America, Asia, and Africa (12).

*Salmonella* subtypes below the subspecies classification have traditionally been determined using serotyping, which may help pinpoint the origins of the *Salmonella* isolates, determine the severity of the disease, and assess whether or not they are resistant to antibiotics (13). Therefore, determining *Salmonella* serotypes continues to be a crucial diagnostic necessity in the promotion of health (14). However, traditional *Salmonella* serotyping is a laborious method that involves several different typing antisera in addition to taking between 5 and 6 days to finish the overall experiment (15). Moreover, the biovars *S*. Pullorum and *S*. Gallinarum are antigenically similar, making it challenging to distinguish between them following the isolation of serovar Gallinarum. Despite this similarity, they each produce diseases with distinct clinical presentations and transmission patterns, and thus, it is crucial to distinguish between the two biovars. Biochemical assays were conducted in a previous study to distinguish between the two strains of bacteria by observing how they fermented ornithine, spironolactone, dulcitol, and maltose (16). This can be accomplished biochemically, but it will take between 2 and 3 days (17). Therefore, there is an immediate need for a technique of diagnostics that is both quick and inexpensive to recognize and distinguish between *S*. Gallinarum and *S*. Pullorum, as early identification of the pathogen helps in directing the prevention and control of pathogens (18).

The great specificity and sensitivity of polymerase chain reaction (PCR) has shown considerable potential in the screening of many infections due to the advancement of molecular biological methods. Notably, even highly related strains and variants of bacteria have been successfully identified and differentiated using PCR (19). The effectiveness of various PCR tests to distinguish various *Salmonella* serovars has been studied in separate investigations, which demonstrated the remarkable sensitivity and specificity of the PCR assay (20, 21).

In the present study, we developed and validated an accurate multiplex PCR assay to simultaneously detect and differentiate biovars *S*. Gallinarum and *S*. Pullorum. The multiplex PCR was based on three specific genes of *torT, I137_14430* and *stn*. The specificity and sensitivity of the multiplex PCR assay were evaluated for this assay. This newly developed method was applied to identify the two biovars *S*. Gallinarum and *S*. Pullorum in clinical samples from a chicken farm.

## 2. Materials and methods

### 2.1. Bacterial strains

The assays were conducted with pure, serologically characterized strains for further studies. Supplementary Table S1 lists the *Salmonella* and non-*Salmonella* pathogens utilized to develop and validate the multiplex PCR technique. A total of 75 *Salmonella* isolates including 29 different serovars and 50 non-*Salmonella* pathogens were obtained in routine laboratory work and used in the present study. Rapid agglutination diagnostic antisera (Tianrun Bio-Pharmaceutical, Ningbo, China) were used for serotyping all identified strains of *Salmonella*. The biovars Gallinarum and Pullorum were distinguished from one another with the aid of fermenting dulcitol and decarboxylating ornithine.

### 2.2. Bacterial growth and genomic DNA extraction

Frozen stocks of the isolates were recovered in Luria-Bertani (LB) agar (Oxoid, Basingstoke, UK) or brain heart infusion (BHI) agar (Becton, Dickinson and Company, Sparks, MD, United States) for 18 h at 37°C for DNA purification. The colonies were inoculated into LB or BHI broth and incubated overnight at 37°C with continuous shaking at 180 rpm.

Following the protocol outlined by the manufacturer of a DNA extraction kit (Tiangen, Beijing, China), genomic DNA was isolated from the cultured bacteria. Afterward, the DNA was resuspended with 100 μL of double distilled sterile water. A NanoDrop ND-1000 (Thermo Scientific, Wilmington, DE) was utilized to analyze the level and purity of the extracted genomic DNA. The extracted DNA was stored at−20°C in preparation for the PCR tests.

### 2.3. *In silico* analysis and specific primer design

The specificity of two candidate genes, *torT*, and *I137_14430*, was examined to establish an easy-to-use, quick, and reproducible approach for identifying and distinguishing *S*. Gallinarum and *S*. Pullorum. The genes *torT* (GenBank acc. no. AM933173.1, region 3797861-3798901) and *I137_14430* (GenBank acc. no. CP006575.1, region 3085007-3085765) were identified by screening the database for non-redundant nucleotide collection (nr/nt) utilizing the basic local alignment search tool (BLAST). All other settings were left at their defaults except for the upper limit on how many aligned sequences to be displayed, which was set at the maximum of 5,000. Sequence alignments of *I137_14430* and *torT* from *S*. Gallinarum, *S*. Pullorum and other serovars were conducted by Clustal W. BLAST search was conducted using *S*. Pullorum *torT, I137_14430* and *stn* nucleotide sequences against the *torT* or genome sequences of *Citrobacter freundii* strain FDAARGOS_549 (GenBank accession no. NZ_CP033744.1).

Three sets of primers of *torT, I137_14430* and *stn* were designed. The positions of the primers were based on the deficient region of *torT*, the unique sequence of *I137_14430*, and the conserved sequence of *stn* for *Salmonella* genus. The first primer set, *troT*-F/R, was designed to amplify an 801 bp fragment to allow for the specific identification of *S*. Pullorum. The second primer set, *I137_14430*-F/R, was designed to distinguish *S*. Gallinarum from other strains, while the third primer set, *stn*-F/R, was used as a reference gene to identify *Salmonella* genus (Table 1). The specificity of primers was assessed by using the BLAST and primers were synthesized commercially by GenScript (Nanjing, China).

**Table 1:** Primer sequences for the specific detection and differentiation of *S*. Gallinarum and *S*. Pullorum with the multiplex PCR system.

**Primers**	**Primer sequence (5^′^ → 3^′^)**	**Size (bp)**	**GenBank acc. no./Nt segments**	***Salmonella*** **serovars**		
				**SP**	**SG**	**non-SP/SG**
*torT F*	AAAAGCGGCCAAATTGTATGGC	801	AM933173.1 3798073-3798873	–	+	+
*torT R*	TACAAATAGACGGGCCGAAATC					
*I137_14430* F	ATTTGTCCTGCCAGATTTGCTTC	508	CP006575.1 3085114-3085621	+	–	+
*I137_14430* R	CAGCAATTACGTCGGAAACCGGAG					
*stn* F	GTTCGAGCAATTCGCTTACCAC	252	L16014.1 831-1082	+	+	+
*stn* R	TTTGGCATCAGCGTTATCAGCG					

SP, *S*. Pullorum; SG, *S*. Gallinarum.

### 2.4. Optimization and development of the multiplex PCR assay

PCR tests were conducted in a 25 μL reaction solution composed of 12.5 μL of 2 × Taq Master mix (Vazyme, Nanjing, China), primer concentrations including 80 nM *stn*, 40 nM *torT*, and 40 nM *I137_14430*, genomic DNA from bacteria (100 ng), and highly purified water was added till the total amount reached 25 μL. PCR amplification was conducted in a T100 thermal cycler (Bio-Rad, Hercules, CA, USA) with an initial denaturation of 94°C for 3 min, 25 cycles of 94°C for 45 s, 60°C for 30 s, and 72°C for 60 s, followed by a final extension at 72°C for 10 min. Following electrophoresis, the PCR products were examined on a 1% agarose gel before staining with GelRed Nucleic Acid Gel Stain (Biotium, Fremont, CA, USA), and subsequent visualization under UV light using a GelDoc XR Gel Documentation System (Bio-Rad).

### 2.5. Specificity of the multiplex PCR assay

Genomic DNA was taken from 75 *Salmonella* strains representing 29 distinct serovars and 50 non-*Salmonella* pathogens presented in Supplementary Table S1, in order to test the specificity as well as the compatibility of the primer sequences in the established multiplex PCR. Specificity of the one-step multiplex PCR for the detection and differentiation of *S*. Pullorum and *S*. Gallinarum was cross-validated in another laboratory. Two strains were randomly selected for each *Salmonella* serotype and non-*Salmonella* pathogens.

### 2.6. Sensitivity of the PCR assay

To determine the limit of detection in the PCR test, its sensitivity was assessed. *S*. Pullorum strain S06004 and *S*. Gallinarum strain SG9 were grown overnight in the LB medium and the DNA was extracted with a bacterial genomic DNA extraction kit. The genomic DNA was consecutively diluted from 27.5 ng/μL to 2.75 pg/μL in sterile water and served as the templates for the multiplex PCR. Two washes with PBS were used to rinse the bacterial culture of *S*. Gallinarum and *S*. Pullorum, adjusted to match the final concentration levels of 2 × 10^7^ to 2 × 10^3^ CFU/mL, and DNA from bacterial genomes were extracted by boiling for 10 min in a water bath. Eventually, 5 μL of each concentration recovered via centrifugation was utilized for this PCR test to determine the lowest number of *S*. Pullorum and *S*. Gallinarum cells.

### 2.7. Implementation of multiplex PCR test on chicken egg samples

The clinical *Salmonella* strains were isolated from dead eggs obtained from a poultry farm in Jiangsu, China. *Salmonella* was isolated from samples by following previously established procedures for sample processing, enrichment, and isolation (22, 23). Briefly, all the samples underwent pre-enrichment in 50 mL of buffered peptone water (Difco, BD, Sparks, MD, United States) for 24 h at 37°C. After being streaked over xylose lysine tergitol 4 (Difco, BD) agar, the bacterial culture was subjected to incubation for 16 h at 37°C. The established multiplex PCR approach was employed to detect the DNA from the putative *Salmonella* colonies. Moreover, each sample was also subjected to the standard bacterial culture procedures as well as the traditional serum agglutination test.

## 3. Results and discussion

### 3.1. Evaluation of sequence alignment and the development of primers

Turkeys, chickens, and a few other types of birds are susceptible to two different strains of *Salmonella*, called Gallinarum and Pullorum, which correspondingly cause fowl typhoid and Pullorum disease (8). Since *S*. Pullorum and *S*. Gallinarum are both members of the same serovar yet belong to separate biovars, biochemical features are the primary basis for identifying and distinguishing between them. Even though biochemical identification is the most common method, analyzing a large number of samples rapidly may be difficult, expensive, and time-consuming. Consequently, DNA-based approaches, particularly PCR-based methods, were required for distinguishing between the two closely related biovars. Molecular screening with the use of PCR is the most time-efficient method, as it has both a high degree of specificity and sensitivity and could be applied for the prompt detection and characterization of particular types of pathogenic microbial infection (24, 25).

**Figure 1:**
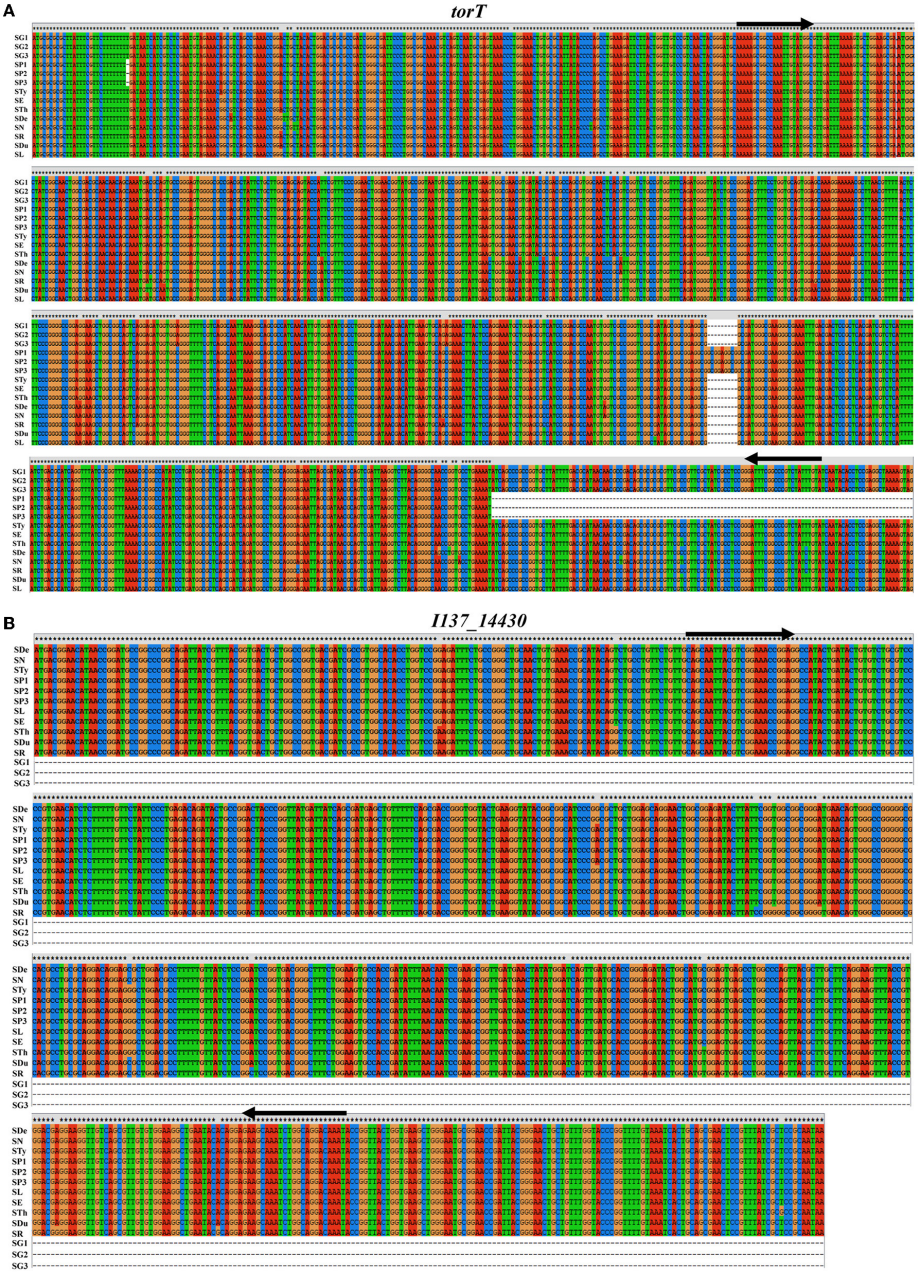
Sequence alignment of *torT* and *I137_14430* genes from *S*. Gallinarum, *S*. Pullorum and other serovars. **(A)** A 126 bp-region of deletion in the carboxyl terminal of *torT* was observed only in *S*. Pullorum after comparison with that of *S*. Gallinarum and other *Salmonella* serovars, which could be used for the specific identification of *S*. Pullorum. **(B)** The *I137_14430* gene is present in all *Salmonella* serovars except for *S*. Gallinarum, and this discrepancy could be used for the identification of *S*. Gallinarum. The designed primers are indicated with the black arrows. SG1, *S*. Gallinarum str. 9184 (GenBank acc. no. CP019035.1); SG2, *S*. Gallinarum str. 07Q015 (GenBank acc. no. CP077760.1); SG3, *S*. Gallinarum str. 287/91 (GenBank acc. no. AM933173.1); SP1, *S*. Pullorum str. S06004 (GenBank acc. no. CP006575.1); SP2, *S*. Pullorum str. R51 (GenBank acc. no. CP068386.1); SP3, *S*. Pullorum str. QJ-2D-Sal (GenBank acc. no. CP022963.1); STy, *S*. Typhimurium str. SCPM-O-B-4515 (GenBank acc. no. CP088136.1); SE, *S*. Enteritidis str. SE95 (GenBank acc. no. CP050716.1); STh, *S*. Thompson str. SH11G0791 (GenBank acc. no. CP041171.1); SDe, *S*. Derby str. FDA161736 (GenBank acc. no. CP075036.1); SN, *S*. Newport str. SAP18-8729 (GenBank acc. no. CP041208.1); SR, *S*. Rissen str. GJ0703-2 (GenBank acc. no. CP043509.1); SDu, *S*. Dublin str. USMARC-69807 (GenBank acc. no. CP032379.1); SL, *S*. London str. L1 (GenBank acc. no. CP117698.1).

The selection of the target is one of the most crucial aspects involved in the development of this kind of detection assay. Based on the bioinformatics analysis, we found that a 126 bp-region of deletion in the carboxyl-terminal of *torT* was observed only in *S*. Pullorum and not in other *Salmonella* serovars including *S*. Gallinarum, which may be employed to accurately identify *S*. Pullorum (Figure 1A; Supplementary Figure S1). The *I137_14430* gene, which is found in all *Salmonella* serovars besides *S*. Gallinarum, could be utilized to identify this biovar (Figure 1B; Supplementary Figure S2). The similarity and existence of the three targets were also examined in *C. freundii*. The results show that the length of *S*. Pullorum *torT* gene is 923 bp. However, the length of *C. freundii torT* gene is 1,032 bp. Besides, no significant similarity was found in the *torT* sequences between the two species (Supplementary Figure S3A). *I137_14430* and *stn* genes of *S*. Pullorum were aligned with the genome of *C. freundii*, and no significant similarity was found in *C. freundii* genome for both *I137_14430* sequence (Supplementary Figure S3B) and *stn* sequence (Supplementary Figure S3C). Furthermore, the gene *stn* is extensively employed as the reference control of the *Salmonella* genus (26–28). Thus, based on the sequence characteristics, three sets of primers of *torT, I137_14430*, and *stn* were designed. The three pairs of primers amplified three specific products for *torT* (801 bp), *I137_14430* (508 bp), and *stn* (252 bp) (Table 1).

Novel gene-based tests for identifying *Salmonella* have also been published. For instance, by examining the whole genome sequencing data of *S*. Pullorum serotypes, Xu et al. (29) effectively designed the PCR test for the recognition of *S*. Pullorum predicated on the novel gene *ipaJ* of *S*. Pullorum. However, it could not differentiate the two biovars of *S*. Gallinarum and *S*. Pullorum. Notably, our research successfully identified and differentiated *S*. Pullorum and *S*. Gallinarum for the first time by targeting the new genes of *torT* and *I137_14430*.

### 3.2. Specificity of the multiplex PCR test for distinguishing *S*. Gallinarum and *S*. Pullorum biovars

*Salmonella* needs to be identified and serotyped to offer additional details that can be used for the determination of the sources of infection during outbreak investigations and for strain identification (30). Nevertheless, the findings of the majority of genotyping techniques, such as plasmid profile analysis, pulsed-field gel electrophoresis (PFGE), ribotyping, and amplified fragment length polymorphism (AFLP), do not show a correlation between the genotype and the serotype of the *Salmonella* serogroup (31).

**Figure 2:**
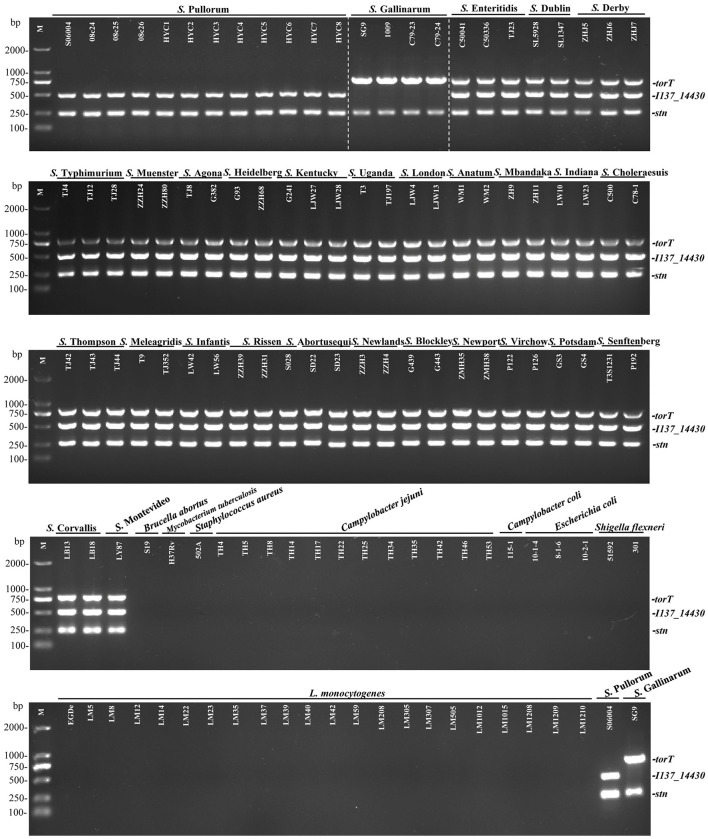
Specificity of the one-step multiplex PCR for the detection and differentiation of *S*. Pullorum and *S*. Gallinarum. Genomic DNA was taken from 75 *Salmonella* strains representing 29 distinct serovars and 43 non-*Salmonella* pathogens presented in Supplementary Table S1, in order to test the specificity as well as the compatibility of the new primer sets of *I137_14430, torT* and *stn*. The PCR amplifies only two specific products of 508-bp *I137_14430* and 252-bp *stn* for *S*. Pullorum. Only two products of 801-bp *torT* and 252-bp *stn* were generated for *S*. Gallinarum. All three products of *torT, I137-14430*, and *stn* were amplified for other *Salmonella* serovars. Nonetheless, there was not a single band generated for any other non-*Salmonella* pathogens. Lane M: DL2000 DNA marker (Takara Biotechnology Co., Dalian, China).

**Figure 3:**
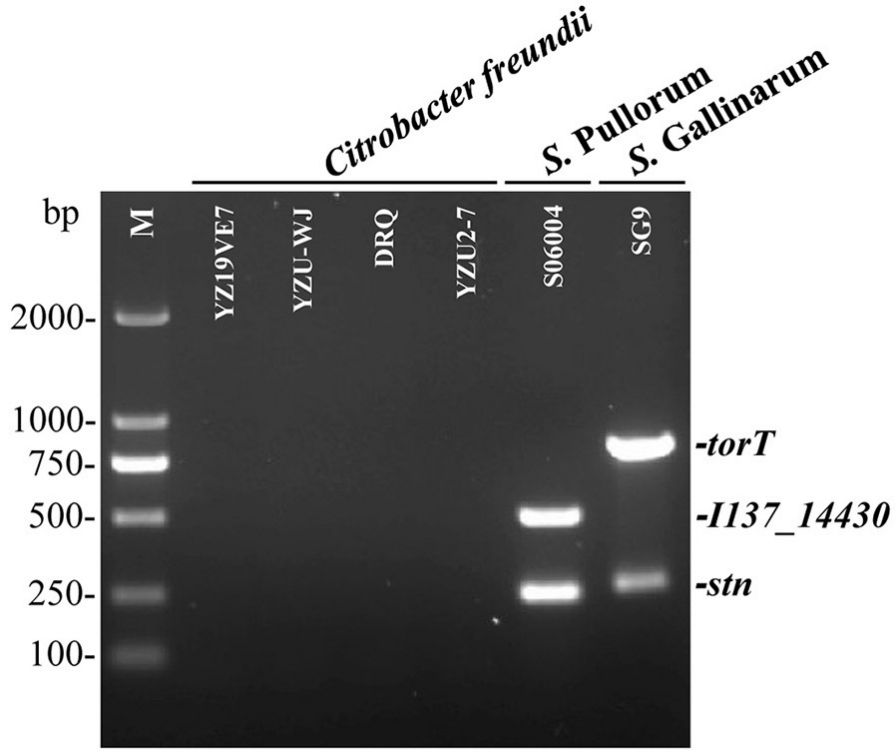
Determination of the accuracy of the one-step multiplex PCR for the identification of *S*. Pullorum and *S*. Gallinarum. Genomic DNA was taken from four strains of *C. freundii* preserved in our laboratory presented in Supplementary Table S1, in order to test the specificity of the designed primer sets of *I137_14430, torT* and *stn*. *S*. Pullorum strain S06004 and *S*. Gallinarum strain SG9 were used as the positive controls. The PCR amplifies only two specific products of 508-bp *I137_14430* and 252-bp *stn* for *S*. Pullorum. Only two products of 801-bp *torT* and 252-bp *stn* were generated for *S*. Gallinarum. Nonetheless, the three specific targets could not be amplified in four strains of *C. freundii* using the designed primers.

To evaluate the specificity of the three primers sets, the multiplex PCR was first optimized and then tested with DNA templates prepared from the 50 non-*Salmonella* and 75 *Salmonella* pathogens as listed in Supplementary Table S1. The results showed that only two specific products of 508-bp *I137_14430* and 252-bp *stn* were amplified for *S*. Pullorum. Only two products of 801-bp *torT* and 252-bp *stn* were generated for *S*. Gallinarum. All three products of *torT, I137_14430*, and *stn* were amplified for other *Salmonella* serovars. Nonetheless, there was not a single band generated for any of the pathogens that were not *Salmonella* (Figure 2). The three specific targets could not be amplified in four strains of *C. freundii* preserved in our laboratory using the designed primers in this study (Figure 3), which was consistent with those of bioinformatics analysis. Cross validation of tests between laboratories was carried out to verify the accuracy of the multiplex PCR assay. Two strains were randomly selected for each *Salmonella* serotype and non-*Salmonella* pathogens (including *Citrobacter spp*.). The results were consistent with those in our laboratory, and this multiplex PCR method could accurately identify and distinguish *S*. Gallinarum and *S*. Pullorum (Supplementary Figure S4), showing that this method has good reproducibility. Additionally, there were no false positives or negatives created in the established PCR method using the three pairs of specific primers. Meanwhile, the multiplex PCR did not indicate any cross-reaction with 100% specificity, in line with the BLAST results. This PCR test had excellent specificity and could effectively identify and differentiate between *S*. Pullorum and *S*. Gallinarum.

Traditionally, *S*. Pullorum was distinguished from *S*. Gallinarum utilizing approaches like PCR restriction fragment length polymorphism (RFLP) and single-strand conformational polymorphism (SSCP) which employ changeable sections of a gene or a single-nucleotide polymorphism (SNP) (32, 33). This research, however, used the *torT* and *I137_14430* genes to definitively tell *S*. Pullorum and *S*. Gallinarum apart. Moreover, as the primers of the *torT* gene are specific for *S*. Pullorum, and the primers of *I137_14430* are specific for *S*. Gallinarum, it would be possible to distinguish the two biovars utilizing the two specific targets independently. Significant time and labor savings are possible since the established multiplex PCR can amplify specific DNA sequences and distinguish between the two *Salmonella* biovars simultaneously.

### 3.3. Sensitivity of the multiplex PCR assay for the identification of biovars Pullorum and Gallinarum

**Figure 4:**
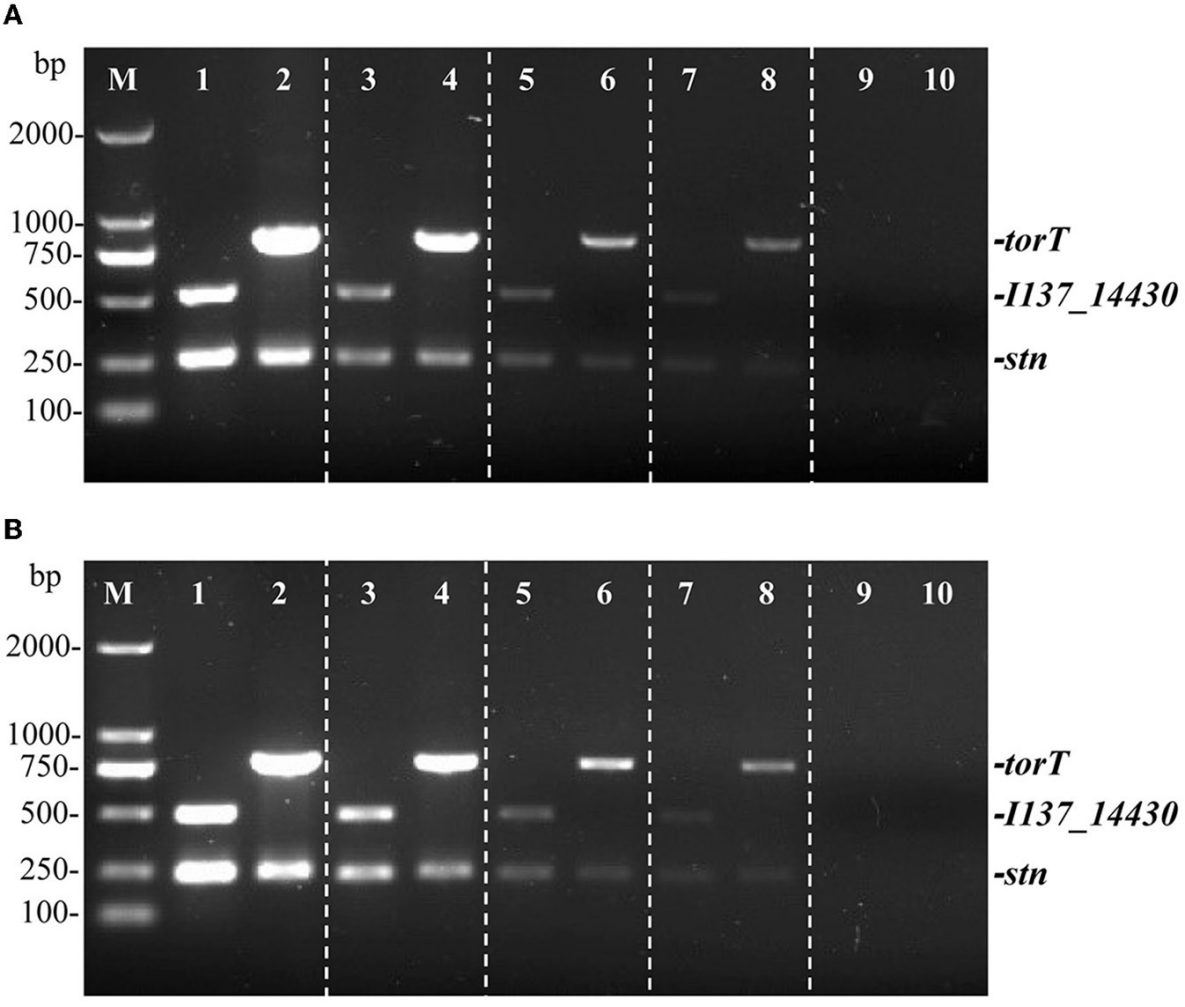
Identifying limit of the multiplex PCR method for the detection of genomic DNA and cells from *S*. Pullorum (S06004) and *S*. Gallinarum (SG9). The multiplex PCR amplifies three specific bands of *torT* (801 bp), *I137_14430* (508 bp) and *stn* (252 bp). The multiplex PCR for the detection of genomic DNA **(A)** and *Salmonella* cells **(B)**, lanes 1, 3, 5, 7, 9 (*S*. Pullorum) and 2, 4, 6, 8, 10 (*S*. Gallinarum): the templates of genomic DNA at the following concentrations, respectively: 27.5, 2.75 ng/μL, 275, 27.5, 2.75 pg/μL; the number of cells per PCR assay at the following concentrations, respectively: 10^5^ CFU, 10^4^ CFU, 10^3^ CFU, 10^2^ CFU and 10^1^ CFU.

Two distinct types of templates were used to determine the sensitivity of the multiplex PCR. The detection limit of the PCR technique was evaluated by serially diluting *S*. Gallinarum and *S*. Pullorum genomic DNA from 27.5 ng/μL to 2.75 pg/μL. It was determined via sensitivity testing that a minimum of 27.5 pg/μL of genomic DNA was needed for the identification of *S*. Pullorum and *S*. Gallinarum following the multiplex PCR detection (Figure 4A). The sensitivity of the developed multiplex PCR is higher than that of the HRM-PCR test, which has a sensitivity of 126.2 pg/μL (34).

Moreover, a ten-fold serial dilution of *S*. Pullorum and *S*. Gallinarum cells that ranged from 10^5^ CFU to 10^1^ CFU was used to determine the detectable limit of bacterial cells. The results from three primer sets confirmed that 100 CFU was the lowest concentration at which *S*. Gallinarum and *S*. Pullorum could be detected (Figure 4B). These PCR results showed a lower threshold for bacterial cell recognition than those from the *sefA* gene-based PCR (400 CFU) (35), comparable to the multiplex PCR centered on *flhB, lygD*, and *tcpS* genes (100 CFU) (36), and the PCR based on the *ipaJ* gene (100 CFU) (29). These findings confirmed that the PCR technique had a superior limit of detection, allowing for the identification of *S*. Pullorum and *S*. Gallinarum cells at very low quantities. Besides, this PCR assay was also very rapid, with a single-round PCR procedure taking < 2 h.

### 3.4. Application of the multiplex PCR in identifying and distinguishing between Pullorum and Gallinarum biovars

Both PD (which is caused by *S*. Pullorum) and FT (which is caused by *S*. Gallinarum) have a tendency to cause severe economic losses of livestock (37). *S*. Pullorum and *S*. Gallinarum are two of the most significant bacterial infections that affect chicken in China (38). The accurate identification and subsequent removal of diseased birds are essential for the prevention and success in eliminating *S*. Gallinarum and *S*. Pullorum in poultry.

To assess the diagnostic performance in terms of the accuracy of our multiplex PCR approach, further clinical samples obtained from naturally contaminated chicken egg specimens were used. The findings of the PCR proved that ten of the twenty-four samples only included the unique 508-bp target of *I137_14430* and the 252-bp target of *stn*, which implied that the isolates of Ch4, Ch5, Ch9, Ch10, Ch11, Ch12, Ch16, Ch17, Ch18 and Ch24 were *S*. Pullorum. Both the unique 801-bp *torT* and 252-bp *stn* were generated by two different isolates, confirming that the two isolates of Ch22 and Ch23 were *S*. Gallinarum. The outcomes of the PCR experiment were consistent with the findings obtained using traditional biochemical reactions and serotyping techniques (Table 2).

**Table 2:** The developed multiplex PCR method was applied for the identification of *S*. Pullorum and *S*. Gallinarum isolates from one chicken farm.

**Serovar (no. of isolates)**	**Isolate no**.	**PCR results (*torT/I137_14430*/*stn*)**	**Dulcitol fermentation**	**Ornithine decarboxylase**
Pullorum (10)	Ch4	−/+/+	–	+
	Ch5	−/+/+	–	+
	Ch9	−/+/+	–	+
	Ch10	−/+/+	–	+
	Ch11	−/+/+	–	+
	Ch12	−/+/+	–	+
	Ch16	−/+/+	–	+
	Ch17	−/+/+	–	+
	Ch18	−/+/+	–	+
	Ch24	−/+/+	–	+
Gallinarum (2)	Ch22	+/−/+	+	–
	Ch23	+/−/+	+	–
Enteritidis (9)	Ch1	+/+/+	/	/
	Ch2	+/+/+	/	/
	Ch3	+/+/+	/	/
	Ch6	+/+/+	/	/
	Ch7	+/+/+	/	/
	Ch8	+/+/+	/	/
	Ch13	+/+/+	/	/
	Ch14	+/+/+	/	/
	Ch15	+/+/+	/	/
Indiana (3)	Ch19	+/+/+	/	/
	Ch20	+/+/+	/	/
	Ch21	+/+/+	/	/

The traditional serotyping of the *Salmonella* isolates was determined based on the White-Kauffmann-Le Minor scheme. The traditional differentiation of *Salmonella* biovars Pullorum and Gallinarum was conducted with the dulcitol fermentation and ornithine decarboxylation. +, positive; −, negative.

Classical microbiological techniques are proven to be less efficient and less sensitive than PCR-based testing in identifying *Salmonella* serovars (5). Molecular techniques, like PCR accompanied by RFLP predicated on the *speC* or *fliC* genes, have been utilized to distinguish between *S*. Gallinarum and *S*. Pullorum to identify these infections (10, 32). Other methods, such as a duplex PCR and a multiplex real-time PCR, have been designed to tell apart between *S*. Pullorum and *S*. Gallinarum (2, 39). However, these methods were expensive and required special equipment. The multiplex PCR established in this study is a perfect fit for this need, and it offers technological assistance for eliminating the serovar Gallinarum in commercial poultry farms.

These findings validated *torT, I137_14430*, and *stn* genes as promising candidates for *S*. Gallinarum and *S*. Pullorum identification and differentiation. *Salmonella* serotyping using the traditional serological method is time-consuming, labor-intensive, and expensive. The technology developed in this study, which is based on multiplex PCR, makes it possible to distinguish *S*. Gallinarum from *S*. Pullorum rapidly and accurately. As poultry may also get infected with *S*. Enteritidis, *S*. Typhimurium, and other serovars, the multiplex PCR may be able to differentiate between *S*. Gallinarum biovars and other *Salmonella* serovars. This test might enable early diagnosis of infections, allowing for more efficient disease management in poultry as well as earlier detection of infections.

## 4. Conclusion

In summary, this study developed a novel multiplex PCR method based on three specific genes of *torT, I137_14430* and *stn* for the first time. The multiplex PCR could simultaneously detect and differentiate the prevalent *S*. Pullorum and *S*. Gallinarum, which exhibited efficient ability of identification and discrimination in cultured bacteria and clinical chicken egg samples. The developed multiplex PCR represents a single-step and economical procedure for the rapid, specific, and sensitive detection of both *S*. Pullorum and *S*. Gallinarum. It may contribute to more timely and efficient control measures on both PD and FT.

## Data availability statement

The datasets presented in this study can be found in online repositories. The names of the repository/repositories and accession number(s) can be found in the article/Supplementary material.

## Author contributions

LS, XJ, and ZP conceived the study and wrote the paper. LS designed the experiments, performed the assays, and analyzed the results. LS, RT, and DX performed the experiments and analyzed the results. All authors have contributed to the manuscript, read, and approved the final manuscript.
