# Supplementary material for: Accurate identification and discrimination of Salmonella enterica serovar Gallinarum biovars Gallinarum and Pullorum by a multiplex PCR based on the new genes of torT and I137_14430
Source: Front Vet Sci. 2023 Jul 5;10:1220118. doi: 10.3389/fvets.2023.1220118 (PMC10354433; doi:10.3389/fvets.2023.1220118)
Supplement: Supplementary file 2 [file Data_Sheet_1.PDF]

BLAST Results

Questions/comments

Job title: torT

**RID**	ZXFFK8UG013 (Expires on 03-02 09:52 am)
**Query ID**	lcl|Query_41565
**Description**	None
**Molecule type**	dna
**Query Length**	1041
**Database Name**	nt
**Description**	Nucleotide collection (nt)
**Program**	BLASTN 2.13.0+

Graphic Summary

Distribution of the top 100 Blast Hits on 2202 subject sequences

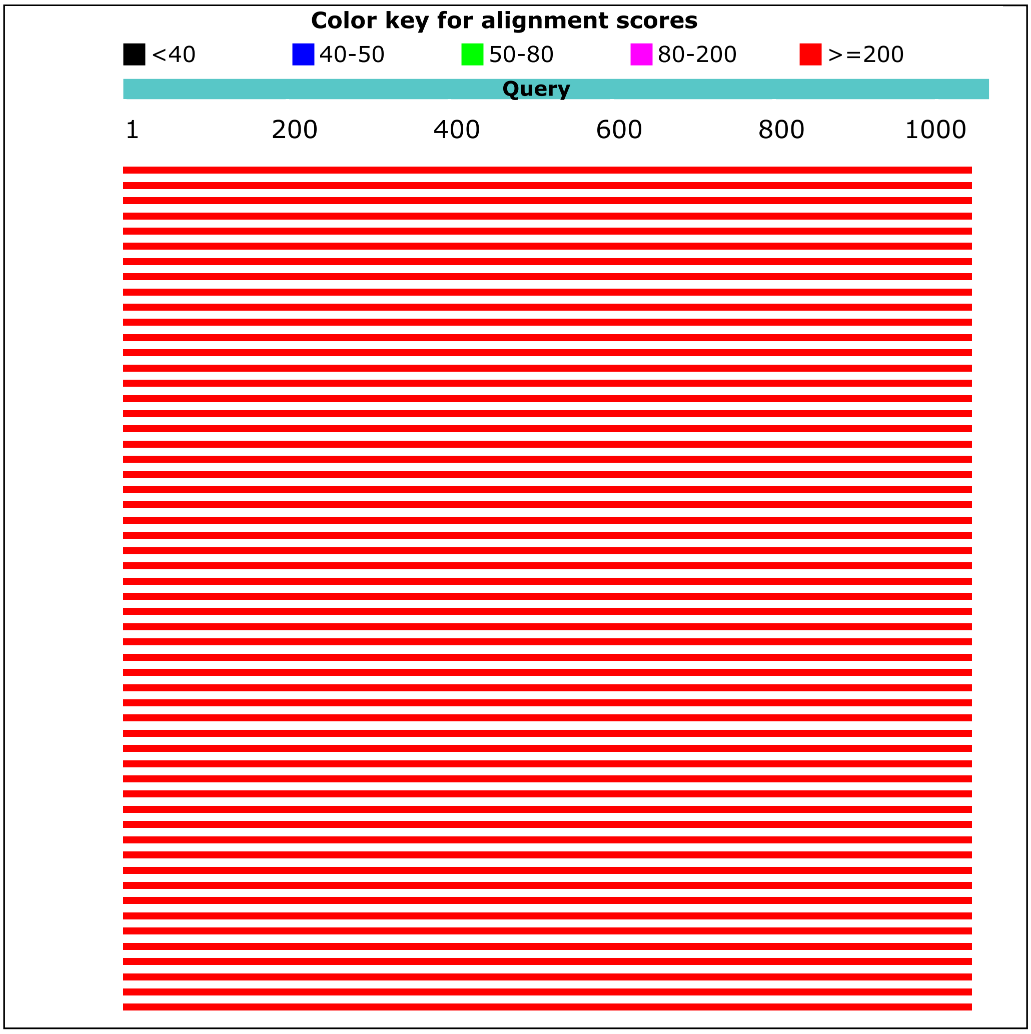

Descriptions

Sequences producing significant alignments:

Description	Max Score	Total Score	Query Cover	E value	Per. Ident	Accession
Salmonella enterica subsp. enterica serovar Gallinarum str. 9184 chromosome, complete genome	1923	1923	100%	0.0	100.00%	CP019035.1
Salmonella enterica subsp. enterica strain SA9R chromosome	1923	1923	100%	0.0	100.00%	CP110508.1
Salmonella enterica subsp. enterica strain SA68 chromosome, complete genome	1923	1923	100%	0.0	100.00%	CP110192.1
Salmonella enterica subsp. enterica serovar Gallinarum strain 07Q015 chromosome, complete genome	1923	1923	100%	0.0	100.00%	CP077760.1
Salmonella enterica subsp. enterica serovar Gallinarum str. 287/91 complete genome	1923	1923	100%	0.0	100.00%	AM933173.1
Salmonella enterica subsp. enterica serovar Typhimurium strain SCPM-O-B-4515 chromosome, complete genome	1912	1912	100%	0.0	99.81%	CP088136.1
Salmonella enterica subsp. enterica serovar Enteritidis strain SE95 chromosome, complete genome	1906	1906	100%	0.0	99.71%	CP050716.1
Salmonella enterica subsp. enterica serovar Enteritidis strain SE74 chromosome, complete genome	1906	1906	100%	0.0	99.71%	CP050723.1
Salmonella enterica subsp. enterica serovar Enteritidis strain SE81 chromosome, complete genome	1906	1906	100%	0.0	99.71%	CP050721.1
Salmonella enterica subsp. enterica serovar Enteritidis strain SE104 chromosome, complete genome	1906	1906	100%	0.0	99.71%	CP050712.1
Salmonella enterica subsp. enterica serovar Enteritidis strain SE109 chromosome, complete genome	1906	1906	100%	0.0	99.71%	CP050709.1
Salmonella enterica subsp. enterica serovar Enteritidis strain SE124 chromosome, complete genome	1906	1906	100%	0.0	99.71%	CP050706.1
Salmonella enterica subsp. enterica serovar Enteritidis strain NCCP 16206 chromosome, complete genome	1906	1906	100%	0.0	99.71%	CP041973.1
Salmonella enterica subsp. enterica serovar Enteritidis strain K12SE001 chromosome	1906	1906	100%	0.0	99.71%	CP043563.1
Salmonella enterica subsp. enterica serovar Enteritidis strain PT1 chromosome, complete genome	1906	1906	100%	0.0	99.71%	CP043433.1
Salmonella enterica subsp. enterica serovar Enteritidis strain SJTUF12367v2 chromosome, complete genome	1906	1906	100%	0.0	99.71%	CP041176.1
Salmonella enterica subsp. enterica serovar Enteritidis strain SJTUF12519v2 chromosome, complete genome	1906	1906	100%	0.0	99.71%	CP041173.1
Salmonella enterica subsp. enterica serovar Enteritidis strain SAP18-H9654 chromosome, complete genome	1906	1906	100%	0.0	99.71%	CP040646.1
Salmonella enterica subsp. enterica serovar Enteritidis strain SAP18-0432 chromosome, complete genome	1906	1906	100%	0.0	99.71%	CP040644.1
Salmonella enterica subsp. enterica serovar Enteritidis strain CFSAN076214 chromosome, complete genome	1906	1906	100%	0.0	99.71%	CP033340.1
Salmonella enterica subsp. enterica serovar Enteritidis strain SEO chromosome, complete genome	1906	1906	100%	0.0	99.71%	CP033090.1

Description	Max Score	Total Score	Query Cover	E value	Per. Ident	Accession
Salmonella enterica subsp. enterica serovar Enteritidis strain NCM 61 chromosome, complete genome	1906	1906	100%	0.0	99.71%	CP032851.1
Salmonella enterica strain FORC_074 chromosome, complete genome	1906	1906	100%	0.0	99.71%	CP023436.1
Salmonella enterica subsp. enterica serovar Enteritidis strain FORC_075 chromosome, complete genome	1906	1906	100%	0.0	99.71%	CP023475.1
Salmonella enterica strain FORC_078 chromosome, complete genome	1906	1906	100%	0.0	99.71%	CP026713.1
Salmonella enterica subsp. enterica serovar Enteritidis strain 2017K-0021 chromosome, complete genome	1906	1906	100%	0.0	99.71%	CP030794.1
Salmonella enterica subsp. enterica serovar Enteritidis str. RM2968 chromosome, complete genome	1906	1906	100%	0.0	99.71%	CP028151.1
Salmonella enterica subsp. enterica serovar Enteritidis strain RM4283 chromosome, complete genome	1906	1906	100%	0.0	99.71%	CP028157.1
Salmonella enterica subsp. enterica serovar Enteritidis str. EC20120916 genome	1906	1906	100%	0.0	99.71%	CP007332.2
Salmonella enterica subsp. enterica serovar Enteritidis strain ATCC BAA-708 chromosome, complete genome	1906	1906	100%	0.0	99.71%	CP025554.1
Salmonella enterica subsp. enterica serovar Enteritidis strain PIR00532 chromosome, complete genome	1906	1906	100%	0.0	99.71%	CP025559.1
Salmonella enterica subsp. enterica serovar Enteritidis strain SE86 chromosome, complete genome	1906	1906	100%	0.0	99.71%	CP019681.1
Salmonella enterica strain MFDS1004839 chromosome, complete genome	1906	1906	100%	0.0	99.71%	CP026569.1
Salmonella enterica strain FDAARGOS_70 chromosome, complete genome	1906	1906	100%	0.0	99.71%	CP026052.1
Salmonella enterica subsp. enterica serovar Typhimurium strain FORC50 chromosome, complete genome	1906	1906	100%	0.0	99.71%	CP019383.1
Salmonella enterica subsp. enterica serovar Enteritidis strain SJTUF10984 chromosome, complete genome	1906	1906	100%	0.0	99.71%	CP015526.1
Salmonella enterica subsp. enterica serovar Enteritidis strain SJTUF10978 chromosome, complete genome	1906	1906	100%	0.0	99.71%	CP015524.1
Salmonella enterica subsp. enterica serovar Enteritidis strain 95-0621 chromosome, complete genome	1906	1906	100%	0.0	99.71%	CP018661.1
Salmonella enterica subsp. enterica serovar Enteritidis strain 93-0639 chromosome, complete genome	1906	1906	100%	0.0	99.71%	CP018659.1
Salmonella enterica subsp. enterica serovar Enteritidis strain 81-1706 chromosome, complete genome	1906	1906	100%	0.0	99.71%	CP018655.1
Salmonella enterica subsp. enterica serovar Enteritidis strain 81-1705 chromosome, complete genome	1906	1906	100%	0.0	99.71%	CP018651.1
Salmonella enterica subsp. enterica serovar Enteritidis strain 81-1607 chromosome, complete genome	1906	1906	100%	0.0	99.71%	CP018648.1
Salmonella enterica subsp. enterica serovar Enteritidis strain 81-1435 chromosome, complete genome	1906	1906	100%	0.0	99.71%	CP018647.1
Salmonella enterica subsp. enterica serovar Enteritidis strain 77-2980 chromosome, complete genome	1906	1906	100%	0.0	99.71%	CP018644.1

Description	Max Score	Total Score	Query Cover	E value	Per. Ident	Accession
Salmonella enterica subsp. enterica serovar Enteritidis strain 74-1357 chromosome, complete genome	1906	1906	100%	0.0	99.71%	CP018642.1
Salmonella enterica subsp. enterica serovar Enteritidis strain 70-1605 chromosome, complete genome	1906	1906	100%	0.0	99.71%	CP018640.1
Salmonella enterica subsp. enterica serovar Enteritidis strain 69-3861 chromosome, complete genome	1906	1906	100%	0.0	99.71%	CP018637.1
Salmonella enterica subsp. enterica serovar Enteritidis strain 56-3991 chromosome, complete genome	1906	1906	100%	0.0	99.71%	CP018635.1
Salmonella enterica strain FORC_051 chromosome, complete genome	1906	1906	100%	0.0	99.71%	CP017232.1
Salmonella enterica strain FORC_056 chromosome, complete genome	1906	1906	100%	0.0	99.71%	CP017177.1
Salmonella enterica subsp. enterica serovar Enteritidis strain FORC_052, complete genome	1906	1906	100%	0.0	99.71%	CP016754.1
Salmonella enterica subsp. enterica serovar Enteritidis strain CFSAN051873, complete genome	1906	1906	100%	0.0	99.71%	CP022003.1
Salmonella enterica subsp. enterica serovar Enteritidis strain CFSAN033543 chromosome, complete genome	1906	1906	100%	0.0	99.71%	CP020825.1
Salmonella enterica subsp. enterica serovar Enteritidis strain CFSAN033541 chromosome, complete genome	1906	1906	100%	0.0	99.71%	CP020823.1
Salmonella enterica subsp. enterica serovar Moscow str. S-1843, complete genome	1906	1906	100%	0.0	99.71%	CP019415.1
Salmonella enterica subsp. enterica serovar Blegdam str. S-1824, complete genome	1906	1906	100%	0.0	99.71%	CP019406.1
Salmonella enterica strain FORC_019, complete genome	1906	1906	100%	0.0	99.71%	CP012396.1
Salmonella enterica subsp. enterica serovar Enteritidis strain OLF-00D989 87-1 chromosome, complete genome	1906	1906	100%	0.0	99.71%	CP011942.1
Salmonella enterica subsp. enterica serovar Enteritidis str. SA20094177 genome	1906	1906	100%	0.0	99.71%	CP007468.2
Salmonella enterica subsp. enterica serovar Enteritidis str. EC20110222 chromosome	1906	1906	100%	0.0	99.71%	CP007323.2
Salmonella enterica subsp. enterica serovar Enteritidis str. EC20120685 chromosome	1906	1906	100%	0.0	99.71%	CP007339.2
Salmonella enterica subsp. enterica serovar Enteritidis str. EC20120213 chromosome	1906	1906	100%	0.0	99.71%	CP007344.2
Salmonella enterica subsp. enterica serovar Enteritidis str. EC20120968 chromosome	1906	1906	100%	0.0	99.71%	CP007378.2
Salmonella enterica subsp. enterica serovar Enteritidis str. EC20122022 chromosome	1906	1906	100%	0.0	99.71%	CP007412.2
Salmonella enterica subsp. enterica serovar Enteritidis str. SA20082034 chromosome, complete genome	1906	1906	100%	0.0	99.71%	CP007425.2
Salmonella enterica subsp. enterica serovar Enteritidis str. EC20111515 chromosome	1906	1906	100%	0.0	99.71%	CP007325.2
Salmonella enterica subsp. enterica serovar Enteritidis str. EC20111510 chromosome	1906	1906	100%	0.0	99.71%	CP007498.2
Salmonella enterica subsp. enterica serovar Enteritidis str. SA20094301 chromosome	1906	1906	100%	0.0	99.71%	CP007469.2
Salmonella enterica subsp. enterica serovar Enteritidis str. SA20084824 chromosome	1906	1906	100%	0.0	99.71%	CP007467.2
Salmonella enterica subsp. enterica serovar Enteritidis str. SA20084644 chromosome	1906	1906	100%	0.0	99.71%	CP007466.2
Salmonella enterica subsp. enterica serovar Enteritidis str. EC20121747 chromosome	1906	1906	100%	0.0	99.71%	CP007464.2

Description	Max Score	Total Score	Query Cover	E value	Per. Ident	Accession
Salmonella enterica subsp. enterica serovar Enteritidis str. EC20120929 chromosome	1906	1906	100%	0.0	99.71%	CP007463.2
Salmonella enterica subsp. enterica serovar Enteritidis str. EC20120009 chromosome	1906	1906	100%	0.0	99.71%	CP007438.2
Salmonella enterica subsp. enterica serovar Enteritidis str. EC20120051 chromosome	1906	1906	100%	0.0	99.71%	CP007433.2
Salmonella enterica subsp. enterica serovar Enteritidis str. EC20121765 chromosome	1906	1906	100%	0.0	99.71%	CP007429.2
Salmonella enterica subsp. enterica serovar Enteritidis str. EC20120677 chromosome	1906	1906	100%	0.0	99.71%	CP007428.2
Salmonella enterica subsp. enterica serovar Enteritidis str. SA20100239 chromosome	1906	1906	100%	0.0	99.71%	CP007427.2
Salmonella enterica subsp. enterica serovar Enteritidis str. EC20100131 chromosome	1906	1906	100%	0.0	99.71%	CP007432.2
Salmonella enterica subsp. enterica serovar Enteritidis str. SA20094682 chromosome	1906	1906	100%	0.0	99.71%	CP007431.2
Salmonella enterica subsp. enterica serovar Enteritidis str. EC20090195 chromosome	1906	1906	100%	0.0	99.71%	CP007430.2
Salmonella enterica subsp. enterica serovar Enteritidis str. SA20085285 chromosome, complete genome	1906	1906	100%	0.0	99.71%	CP007426.2
Salmonella enterica subsp. enterica serovar Enteritidis str. EC20120774 chromosome	1906	1906	100%	0.0	99.71%	CP007404.2
Salmonella enterica subsp. enterica serovar Enteritidis str. EC20120738 chromosome	1906	1906	100%	0.0	99.71%	CP007401.2
Salmonella enterica subsp. enterica serovar Enteritidis str. EC20121542 chromosome	1906	1906	100%	0.0	99.71%	CP007368.2
Salmonella enterica subsp. enterica serovar Enteritidis str. EC20121541 chromosome	1906	1906	100%	0.0	99.71%	CP007367.2
Salmonella enterica subsp. enterica serovar Enteritidis str. EC20121004 chromosome	1906	1906	100%	0.0	99.71%	CP007366.2
Salmonella enterica subsp. enterica serovar Enteritidis str. SA20121703 chromosome	1906	1906	100%	0.0	99.71%	CP007372.2
Salmonella enterica subsp. enterica serovar Enteritidis str. EC20121689 chromosome	1906	1906	100%	0.0	99.71%	CP007371.2
Salmonella enterica subsp. enterica serovar Enteritidis str. EC20121672 chromosome	1906	1906	100%	0.0	99.71%	CP007370.2
Salmonella enterica subsp. enterica serovar Enteritidis str. EC20121671 chromosome	1906	1906	100%	0.0	99.71%	CP007369.2
Salmonella enterica subsp. enterica serovar Enteritidis str. EC20090531 chromosome	1906	1906	100%	0.0	99.71%	CP007422.2
Salmonella enterica subsp. enterica serovar Enteritidis str. EC20090884 chromosome	1906	1906	100%	0.0	99.71%	CP007421.2
Salmonella enterica subsp. enterica serovar Enteritidis str. EC20100103 chromosome	1906	1906	100%	0.0	99.71%	CP007420.2
Salmonella enterica subsp. enterica serovar Enteritidis str. EC20120200 chromosome, complete genome	1906	1906	100%	0.0	99.71%	CP007434.2
Salmonella enterica subsp. enterica serovar Enteritidis str. EC20130348 chromosome	1906	1906	100%	0.0	99.71%	CP007424.2
Salmonella enterica subsp. enterica serovar Enteritidis str. EC20130347 chromosome	1906	1906	100%	0.0	99.71%	CP007423.2
Salmonella enterica subsp. enterica serovar Enteritidis str. EC20130346 chromosome	1906	1906	100%	0.0	99.71%	CP007419.2
Salmonella enterica subsp. enterica serovar Enteritidis str. EC20130345 chromosome	1906	1906	100%	0.0	99.71%	CP007418.2
Salmonella enterica subsp. enterica serovar Enteritidis str. SA20123395 chromosome	1906	1906	100%	0.0	99.71%	CP007417.2
Salmonella enterica subsp. enterica serovar Enteritidis str. EC20122045 chromosome	1906	1906	100%	0.0	99.71%	CP007416.2
Salmonella enterica subsp. enterica serovar Enteritidis str. EC20122033 chromosome	1906	1906	100%	0.0	99.71%	CP007415.2

Description	Max Score	Total Score	Query Cover	E value	Per. Ident	Accession
Salmonella enterica subsp. enterica serovar Enteritidis str. EC20120528 chromosome	1906	1906	100%	0.0	99.71%	CP007354.2
Salmonella enterica subsp. enterica serovar Enteritidis str. SA20100349 chromosome	1906	1906	100%	0.0	99.71%	CP007361.2
Salmonella enterica subsp. enterica serovar Enteritidis str. EC20100134 chromosome	1906	1906	100%	0.0	99.71%	CP007359.2
Salmonella enterica subsp. enterica serovar Enteritidis str. EC20100130 chromosome	1906	1906	100%	0.0	99.71%	CP007358.2
Salmonella enterica subsp. enterica serovar Enteritidis str. EC20100100 chromosome	1906	1906	100%	0.0	99.71%	CP007357.2
Salmonella enterica subsp. enterica serovar Enteritidis str. EC20121825 chromosome	1906	1906	100%	0.0	99.71%	CP007382.2
Salmonella enterica subsp. enterica serovar Enteritidis str. EC20121812 chromosome	1906	1906	100%	0.0	99.71%	CP007381.2
Salmonella enterica subsp. enterica serovar Enteritidis str. EC20120970 chromosome	1906	1906	100%	0.0	99.71%	CP007380.2
Salmonella enterica subsp. enterica serovar Enteritidis str. EC20120969 chromosome	1906	1906	100%	0.0	99.71%	CP007379.2
Salmonella enterica subsp. enterica serovar Enteritidis str. EC20120963 chromosome	1906	1906	100%	0.0	99.71%	CP007377.2
Salmonella enterica subsp. enterica serovar Enteritidis str. EC20120927 chromosome	1906	1906	100%	0.0	99.71%	CP007376.2
Salmonella enterica subsp. enterica serovar Enteritidis str. EC20120994 chromosome	1906	1906	100%	0.0	99.71%	CP007365.2
Salmonella enterica subsp. enterica serovar Enteritidis str. SA20094350 chromosome	1906	1906	100%	0.0	99.71%	CP007311.2
Salmonella enterica subsp. enterica serovar Enteritidis str. SA20094079 chromosome	1906	1906	100%	0.0	99.71%	CP007310.2
Salmonella enterica subsp. enterica serovar Enteritidis str. SA20093977 chromosome	1906	1906	100%	0.0	99.71%	CP007309.2
Salmonella enterica subsp. enterica serovar Enteritidis str. SA20093950 chromosome	1906	1906	100%	0.0	99.71%	CP007308.2
Salmonella enterica subsp. enterica serovar Enteritidis str. SA20093788 genome	1906	1906	100%	0.0	99.71%	CP007307.2
Salmonella enterica subsp. enterica serovar Enteritidis str. SA20092320 chromosome	1906	1906	100%	0.0	99.71%	CP007334.2
Salmonella enterica subsp. enterica serovar Enteritidis str. EC20121177 chromosome, complete genome	1906	1906	100%	0.0	99.71%	CP007333.2
Salmonella enterica subsp. enterica serovar Enteritidis str. EC20120555 chromosome	1906	1906	100%	0.0	99.71%	CP007364.2
Salmonella enterica subsp. enterica serovar Enteritidis str. EC20120548 chromosome	1906	1906	100%	0.0	99.71%	CP007363.2
Salmonella enterica subsp. enterica serovar Enteritidis str. EC20120544 chromosome	1906	1906	100%	0.0	99.71%	CP007362.2
Salmonella enterica subsp. enterica serovar Enteritidis str. EC20121750 chromosome	1906	1906	100%	0.0	99.71%	CP007396.2
Salmonella enterica subsp. enterica serovar Enteritidis str. EC20121746 chromosome	1906	1906	100%	0.0	99.71%	CP007374.2
Salmonella enterica subsp. enterica serovar Enteritidis str. EC20120925 chromosome	1906	1906	100%	0.0	99.71%	CP007375.2
Salmonella enterica subsp. enterica serovar Enteritidis str. EC20120775 chromosome	1906	1906	100%	0.0	99.71%	CP007405.2
Salmonella enterica subsp. enterica serovar Enteritidis str. EC20100325 genome	1906	1906	100%	0.0	99.71%	CP007360.2
Salmonella enterica subsp. enterica serovar Enteritidis str. EC20120505 chromosome	1906	1906	100%	0.0	99.71%	CP007353.2
Salmonella enterica subsp. enterica serovar Enteritidis str. EC20120498 chromosome	1906	1906	100%	0.0	99.71%	CP007352.2
Salmonella enterica subsp. enterica serovar Enteritidis str. EC20120496 chromosome	1906	1906	100%	0.0	99.71%	CP007350.2

Description	Max Score	Total Score	Query Cover	E value	Per. Ident	Accession
Salmonella enterica subsp. enterica serovar Enteritidis str. EC20120469 chromosome	1906	1906	100%	0.0	99.71%	CP007349.2
Salmonella enterica subsp. enterica serovar Enteritidis str. EC20120356 chromosome	1906	1906	100%	0.0	99.71%	CP007348.2
Salmonella enterica subsp. enterica serovar Enteritidis str. EC20120240 chromosome	1906	1906	100%	0.0	99.71%	CP007347.2
Salmonella enterica subsp. enterica serovar Enteritidis str. EC20120229 chromosome	1906	1906	100%	0.0	99.71%	CP007346.2
Salmonella enterica subsp. enterica serovar Enteritidis str. EC20120219 chromosome	1906	1906	100%	0.0	99.71%	CP007345.2
Salmonella enterica subsp. enterica serovar Enteritidis str. EC20120686 chromosome	1906	1906	100%	0.0	99.71%	CP007340.2
Salmonella enterica subsp. enterica serovar Enteritidis str. EC20120597 chromosome	1906	1906	100%	0.0	99.71%	CP007338.2
Salmonella enterica subsp. enterica serovar Enteritidis str. EC20120581 chromosome	1906	1906	100%	0.0	99.71%	CP007336.2
Salmonella enterica subsp. enterica serovar Enteritidis str. EC20120580 chromosome	1906	1906	100%	0.0	99.71%	CP007335.2
Salmonella enterica subsp. enterica serovar Enteritidis str. EC20111576 chromosome	1906	1906	100%	0.0	99.71%	CP007328.2
Salmonella enterica subsp. enterica serovar Enteritidis str. EC20111554 chromosome	1906	1906	100%	0.0	99.71%	CP007326.2
Salmonella enterica subsp. enterica serovar Enteritidis str. EC20111514 chromosome	1906	1906	100%	0.0	99.71%	CP007324.2
Salmonella enterica subsp. enterica serovar Enteritidis str. SA20095440 chromosome, complete genome	1906	1906	100%	0.0	99.71%	CP007319.2
Salmonella enterica subsp. enterica serovar Enteritidis str. SA20094389 genome	1906	1906	100%	0.0	99.71%	CP007314.2
Salmonella enterica subsp. enterica serovar Enteritidis str. SA20094383 genome	1906	1906	100%	0.0	99.71%	CP007313.2
Salmonella enterica subsp. enterica serovar Enteritidis str. SA20094352 complete genome	1906	1906	100%	0.0	99.71%	CP007312.2
Salmonella enterica subsp. enterica serovar Enteritidis str. SA20093784 chromosome	1906	1906	100%	0.0	99.71%	CP007306.2
Salmonella enterica subsp. enterica serovar Enteritidis str. SA20093543 chromosome	1906	1906	100%	0.0	99.71%	CP007305.2
Salmonella enterica subsp. enterica serovar Enteritidis str. SA20093538 chromosome	1906	1906	100%	0.0	99.71%	CP007304.2
Salmonella enterica subsp. enterica serovar Enteritidis str. SA20093430 chromosome	1906	1906	100%	0.0	99.71%	CP007303.2
Salmonella enterica subsp. enterica serovar Enteritidis str. SA20093421 chromosome	1906	1906	100%	0.0	99.71%	CP007302.2
Salmonella enterica subsp. enterica serovar Enteritidis str. EC20100089 chromosome	1906	1906	100%	0.0	99.71%	CP007356.2
Salmonella enterica subsp. enterica serovar Enteritidis str. EC20100088 chromosome	1906	1906	100%	0.0	99.71%	CP007355.2
Salmonella enterica subsp. enterica serovar Enteritidis str. EC20090530 chromosome	1906	1906	100%	0.0	99.71%	CP007298.2
Salmonella enterica subsp. enterica serovar Enteritidis str. SA20090435 chromosome	1906	1906	100%	0.0	99.71%	CP007297.2
Salmonella enterica subsp. enterica serovar Enteritidis str. SA20090419 chromosome	1906	1906	100%	0.0	99.71%	CP007296.2
Salmonella enterica subsp. enterica serovar Enteritidis str. SA19981522 chromosome, complete genome	1906	1906	100%	0.0	99.71%	CP007286.2
Salmonella enterica subsp. enterica serovar Enteritidis str. SA19980677 chromosome, complete genome	1906	1906	100%	0.0	99.71%	CP007285.2
Salmonella enterica subsp. enterica serovar Enteritidis str. SA19970769 chromosome, complete genome	1906	1906	100%	0.0	99.71%	CP007283.2

Description	Max Score	Total Score	Query Cover	E value	Per. Ident	Accession
Salmonella enterica subsp. enterica serovar Enteritidis str. SA19992322 genome	1906	1906	100%	0.0	99.71%	CP007291.2
Salmonella enterica subsp. enterica serovar Enteritidis str. SA19983126 chromosome	1906	1906	100%	0.0	99.71%	CP007290.2
Salmonella enterica subsp. enterica serovar Enteritidis str. SA19982831 chromosome	1906	1906	100%	0.0	99.71%	CP007289.2
Salmonella enterica subsp. enterica serovar Enteritidis str. SA19981857 chromosome, complete genome	1906	1906	100%	0.0	99.71%	CP007288.2
Salmonella enterica subsp. enterica serovar Enteritidis str. SA19930684 chromosome	1906	1906	100%	0.0	99.71%	CP007277.2
Salmonella enterica subsp. enterica serovar Enteritidis str. SA20084384 chromosome	1906	1906	100%	0.0	99.71%	CP007295.2
Salmonella enterica subsp. enterica serovar Enteritidis str. SA20083636 chromosome, complete genome	1906	1906	100%	0.0	99.71%	CP007294.2
Salmonella enterica subsp. enterica serovar Enteritidis str. SA20083456 chromosome	1906	1906	100%	0.0	99.71%	CP007293.2
Salmonella enterica subsp. enterica serovar Enteritidis str. SA19994216 chromosome, complete genome	1906	1906	100%	0.0	99.71%	CP007292.2
Salmonella enterica subsp. enterica serovar Enteritidis str. SA19970510 chromosome, complete genome	1906	1906	100%	0.0	99.71%	CP007282.2
Salmonella enterica subsp. enterica serovar Enteritidis str. SA19961622 chromosome	1906	1906	100%	0.0	99.71%	CP007281.2
Salmonella enterica subsp. enterica serovar Enteritidis str. SA19960848 chromosome, complete genome	1906	1906	100%	0.0	99.71%	CP007280.2
Salmonella enterica subsp. enterica serovar Enteritidis str. SA19943269 chromosome	1906	1906	100%	0.0	99.71%	CP007279.2
Salmonella enterica subsp. enterica serovar Enteritidis str. SA19942384 chromosome	1906	1906	100%	0.0	99.71%	CP007278.2
Salmonella enterica subsp. enterica serovar Enteritidis str. SA20091739 chromosome	1906	1906	100%	0.0	99.71%	CP007301.2
Salmonella enterica subsp. enterica serovar Enteritidis str. SA20090877 chromosome	1906	1906	100%	0.0	99.71%	CP007300.2
Salmonella enterica subsp. enterica serovar Enteritidis str. SA20093266 chromosome	1906	1906	100%	0.0	99.71%	CP007274.2
Salmonella enterica subsp. enterica serovar Enteritidis str. EC20121180 chromosome, complete genome	1906	1906	100%	0.0	99.71%	CP007273.2
Salmonella enterica subsp. enterica serovar Enteritidis str. EC20121179 chromosome, complete genome	1906	1906	100%	0.0	99.71%	CP007272.2
Salmonella enterica subsp. enterica serovar Enteritidis str. EC20121178 chromosome, complete genome	1906	1906	100%	0.0	99.71%	CP007271.2
Salmonella enterica subsp. enterica serovar Enteritidis str. EC20121176 chromosome	1906	1906	100%	0.0	99.71%	CP007270.2
Salmonella enterica subsp. enterica serovar Enteritidis str. EC20122031 chromosome	1906	1906	100%	0.0	99.71%	CP007414.2
Salmonella enterica subsp. enterica serovar Enteritidis str. EC20122026 chromosome	1906	1906	100%	0.0	99.71%	CP007413.2
Salmonella enterica subsp. enterica serovar Enteritidis str. EC20121990 chromosome	1906	1906	100%	0.0	99.71%	CP007411.2
Salmonella enterica subsp. enterica serovar Enteritidis str. SA19971331 chromosome	1906	1906	100%	0.0	99.71%	CP007284.2
Salmonella enterica subsp. enterica serovar Enteritidis str. EC20110223 chromosome	1906	1906	100%	0.0	99.71%	CP007266.2
Salmonella enterica subsp. enterica serovar Enteritidis str. EC20120918 chromosome	1906	1906	100%	0.0	99.71%	CP007408.2

Description	Max Score	Total Score	Query Cover	E value	Per. Ident	Accession
Salmonella enterica subsp. enterica serovar Enteritidis str. EC20120917 chromosome	1906	1906	100%	0.0	99.71%	CP007407.2
Salmonella enterica subsp. enterica serovar Enteritidis str. EC20120776 chromosome	1906	1906	100%	0.0	99.71%	CP007406.2
Salmonella enterica subsp. enterica serovar Enteritidis str. EC20120773 chromosome	1906	1906	100%	0.0	99.71%	CP007403.2
Salmonella enterica subsp. enterica serovar Enteritidis str. EC20120765 chromosome	1906	1906	100%	0.0	99.71%	CP007402.2
Salmonella enterica subsp. enterica serovar Enteritidis str. EC20120734 chromosome	1906	1906	100%	0.0	99.71%	CP007400.2
Salmonella enterica subsp. enterica serovar Enteritidis str. EC20121753 genome	1906	1906	100%	0.0	99.71%	CP007398.2
Salmonella enterica subsp. enterica serovar Enteritidis str. EC20121751 genome	1906	1906	100%	0.0	99.71%	CP007397.2
Salmonella enterica subsp. enterica serovar Enteritidis str. EC20121748 genome	1906	1906	100%	0.0	99.71%	CP007395.2
Salmonella enterica subsp. enterica serovar Enteritidis str. EC20121989 chromosome	1906	1906	100%	0.0	99.71%	CP007388.2
Salmonella enterica subsp. enterica serovar Enteritidis str. EC20121986 genome	1906	1906	100%	0.0	99.71%	CP007387.2
Salmonella enterica subsp. enterica serovar Enteritidis str. EC20121976 genome	1906	1906	100%	0.0	99.71%	CP007386.2
Salmonella enterica subsp. enterica serovar Enteritidis str. EC20121970 genome	1906	1906	100%	0.0	99.71%	CP007385.2
Salmonella enterica subsp. enterica serovar Enteritidis str. EC20121969 genome	1906	1906	100%	0.0	99.71%	CP007384.2
Salmonella enterica subsp. enterica serovar Enteritidis str. EC20121826 genome	1906	1906	100%	0.0	99.71%	CP007383.2
Salmonella enterica subsp. enterica serovar Enteritidis str. EC20120722 genome	1906	1906	100%	0.0	99.71%	CP007343.2
Salmonella enterica subsp. enterica serovar Enteritidis str. EC20120697 genome	1906	1906	100%	0.0	99.71%	CP007342.2
Salmonella enterica subsp. enterica serovar Enteritidis str. EC20120007 chromosome	1906	1906	100%	0.0	99.71%	CP007331.2
Salmonella enterica subsp. enterica serovar Enteritidis str. EC20120003 chromosome	1906	1906	100%	0.0	99.71%	CP007330.2
Salmonella enterica subsp. enterica serovar Enteritidis str. EC20111561 chromosome	1906	1906	100%	0.0	99.71%	CP007327.2
Salmonella enterica subsp. enterica serovar Enteritidis str. EC20090332 chromosome	1906	1906	100%	0.0	99.71%	CP007322.2
Salmonella enterica subsp. enterica serovar Enteritidis str. EC20090193 chromosome	1906	1906	100%	0.0	99.71%	CP007321.2
Salmonella enterica subsp. enterica serovar Enteritidis str. EC20090135 chromosome	1906	1906	100%	0.0	99.71%	CP007320.2
Salmonella enterica subsp. enterica serovar Enteritidis str. SA20095309 chromosome	1906	1906	100%	0.0	99.71%	CP007318.2
Salmonella enterica subsp. enterica serovar Enteritidis str. SA20094803 chromosome	1906	1906	100%	0.0	99.71%	CP007317.2
Salmonella enterica subsp. enterica serovar Enteritidis str. SA20094642 genome	1906	1906	100%	0.0	99.71%	CP007316.2
Salmonella enterica subsp. enterica serovar Enteritidis str. SA20094521 genome	1906	1906	100%	0.0	99.71%	CP007315.2
Salmonella enterica subsp. enterica serovar Enteritidis str. EC20120590 genome	1906	1906	100%	0.0	99.71%	CP007337.2
Salmonella enterica subsp. enterica serovar Enteritidis strain CMCC50041 chromosome, complete genome	1906	1906	100%	0.0	99.71%	CP013097.1
Salmonella enterica subsp. enterica serovar Enteritidis strain FORC_007 chromosome, complete genome	1906	1906	100%	0.0	99.71%	CP009768.1

Description	Max Score	Total Score	Query Cover	E value	Per. Ident	Accession
Salmonella enterica subsp. enterica serovar Enteritidis str. EC20120005, complete genome	1906	1906	100%	0.0	99.71%	CP007267.2
Salmonella enterica subsp. enterica serovar Enteritidis str. EC20120002 genome	1906	1906	100%	0.0	99.71%	CP007329.2
Salmonella enterica subsp. enterica serovar Enteritidis str. EC20090641, complete genome	1906	1906	100%	0.0	99.71%	CP007249.2
Salmonella enterica strain CYX chromosome, complete genome	1906	1906	100%	0.0	99.71%	CP113540.1
Salmonella enterica subsp. enterica serovar Enteritidis str. 18569, complete genome	1906	1906	100%	0.0	99.71%	CP011394.1
Salmonella enterica subsp. enterica serovar Enteritidis strain MFDS1018147 chromosome, complete genome	1906	1906	100%	0.0	99.71%	CP110220.1
Salmonella enterica strain 190610_1 chromosome, complete genome	1906	1906	100%	0.0	99.71%	CP060678.1
Salmonella enterica strain 190704_2 chromosome, complete genome	1906	1906	100%	0.0	99.71%	CP060676.1
Salmonella enterica strain 190729_4 chromosome, complete genome	1906	1906	100%	0.0	99.71%	CP060674.1
Salmonella enterica strain 190729_8 chromosome, complete genome	1906	1906	100%	0.0	99.71%	CP060672.1
Salmonella enterica strain 190807_1 chromosome, complete genome	1906	1906	100%	0.0	99.71%	CP060670.1
Salmonella enterica strain 190819_2 chromosome, complete genome	1906	1906	100%	0.0	99.71%	CP060668.1
Salmonella enterica strain 190821_1 chromosome, complete genome	1906	1906	100%	0.0	99.71%	CP060666.1
Salmonella enterica strain PNUSAS119065 chromosome, complete genome	1906	1906	100%	0.0	99.71%	CP104358.1
Salmonella enterica strain PNUSAS118606 chromosome, complete genome	1906	1906	100%	0.0	99.71%	CP104362.1
Salmonella sp. FORC89 chromosome, complete genome	1906	1906	100%	0.0	99.71%	CP029682.1
Salmonella enterica subsp. enterica serovar Enteritidis strain OLF-SE2-98984-6, complete genome	1906	1906	100%	0.0	99.71%	CP009084.2
Salmonella enterica subsp. enterica serovar Enteritidis strain OLF-SE3-98983-4, complete genome	1906	1906	100%	0.0	99.71%	CP009085.2
Salmonella enterica subsp. enterica serovar Enteritidis strain R17.1476 chromosome, complete genome	1906	1906	100%	0.0	99.71%	CP100724.1
Salmonella enterica subsp. enterica serovar Enteritidis strain R18.1630 chromosome, complete genome	1906	1906	100%	0.0	99.71%	CP100666.1
Salmonella enterica subsp. enterica serovar Enteritidis strain SE006 chromosome, complete genome	1906	1906	100%	0.0	99.71%	CP099973.1
Salmonella enterica subsp. enterica strain B3-6 chromosome, complete genome	1906	1906	100%	0.0	99.71%	CP097262.1
Salmonella enterica subsp. enterica serovar Enteritidis strain PNUSAS034908 chromosome, complete genome	1906	1906	100%	0.0	99.71%	CP092321.1
Salmonella enterica strain PNUSAS011707 chromosome, complete genome	1906	1906	100%	0.0	99.71%	CP092314.1
Salmonella enterica strain PNUSAS020938 chromosome, complete genome	1906	1906	100%	0.0	99.71%	CP092326.1
Salmonella enterica subsp. enterica serovar Enteritidis strain SEJ, complete genome	1906	1906	100%	0.0	99.71%	CP008928.1
Salmonella enterica subsp. enterica serovar Enteritidis strain OLF-SE6-00219-16, complete genome	1906	1906	100%	0.0	99.71%	CP009088.1

Description	Max Score	Total Score	Query Cover	E value	Per. Ident	Accession
Salmonella enterica subsp. enterica serovar Enteritidis strain OLF-SE1-1019-1, complete genome	1906	1906	100%	0.0	99.71%	CP009083.1
Salmonella enterica subsp. enterica serovar Enteritidis strain OLF-SE5-1104-2, complete genome	1906	1906	100%	0.0	99.71%	CP009087.1
Salmonella enterica subsp. enterica serovar Enteritidis strain OLF-SE4-0317-8, complete genome	1906	1906	100%	0.0	99.71%	CP009086.1
Salmonella enterica subsp. enterica serovar Enteritidis strain OLF-SE11-10058, complete genome	1906	1906	100%	0.0	99.71%	CP009093.1
Salmonella enterica subsp. enterica serovar Enteritidis strain OLF-SE10-10052, complete genome	1906	1906	100%	0.0	99.71%	CP009092.1
Salmonella enterica subsp. enterica serovar Enteritidis strain OLF-SE9-10012, complete genome	1906	1906	100%	0.0	99.71%	CP009091.1
Salmonella enterica subsp. enterica serovar Enteritidis strain OLF-SE8-1021710, complete genome	1906	1906	100%	0.0	99.71%	CP009090.1
Salmonella enterica subsp. enterica serovar Enteritidis strain OLF-SE7-100819, complete genome	1906	1906	100%	0.0	99.71%	CP009089.1
Salmonella enterica subsp. enterica serovar Enteritidis str. 77-1427, complete genome	1906	1906	100%	0.0	99.71%	CP007598.1
Salmonella enterica subsp. enterica serovar Enteritidis strain Charizard chromosome, complete genome	1906	1906	100%	0.0	99.71%	CP085821.1
Salmonella enterica subsp. enterica serovar Enteritidis strain Ivysaur chromosome, complete genome	1906	1906	100%	0.0	99.71%	CP085824.1
Salmonella enterica subsp. enterica serovar Enteritidis strain Charmeleon chromosome, complete genome	1906	1906	100%	0.0	99.71%	CP085822.1
Salmonella enterica subsp. enterica serovar Enteritidis strain Clefairy chromosome, complete genome	1906	1906	100%	0.0	99.71%	CP085818.1
Salmonella enterica subsp. enterica serovar Enteritidis strain Venusaur chromosome, complete genome	1906	1906	100%	0.0	99.71%	CP085823.1
Salmonella enterica subsp. enterica serovar Enteritidis strain Azumarill chromosome, complete genome	1906	1906	100%	0.0	99.71%	CP085805.1
Salmonella enterica subsp. enterica serovar Enteritidis strain Marill chromosome, complete genome	1906	1906	100%	0.0	99.71%	CP085806.1
Salmonella enterica subsp. enterica serovar Enteritidis strain Clefable chromosome, complete genome	1906	1906	100%	0.0	99.71%	CP085817.1
Salmonella enterica subsp. enterica serovar Enteritidis strain Wailmer chromosome, complete genome	1906	1906	100%	0.0	99.71%	CP085802.1
Salmonella enterica subsp. enterica serovar Enteritidis strain Glaceon chromosome, complete genome	1906	1906	100%	0.0	99.71%	CP085803.1
Salmonella enterica subsp. enterica serovar Enteritidis strain Leafeon chromosome, complete genome	1906	1906	100%	0.0	99.71%	CP085804.1
Salmonella enterica subsp. enterica serovar Enteritidis strain Wailord chromosome, complete genome	1906	1906	100%	0.0	99.71%	CP085801.1
Salmonella enterica subsp. enterica serovar Enteritidis strain Taillow chromosome, complete genome	1906	1906	100%	0.0	99.71%	CP085800.1

Description	Max Score	Total Score	Query Cover	E value	Per. Ident	Accession
Salmonella enterica subsp. enterica serovar Enteritidis strain Swellow chromosome, complete genome	1906	1906	100%	0.0	99.71%	CP085799.1
Salmonella enterica subsp. enterica serovar Enteritidis strain Latios chromosome, complete genome	1906	1906	100%	0.0	99.71%	CP085797.1
Salmonella enterica subsp. enterica serovar Enteritidis strain Latias chromosome, complete genome	1906	1906	100%	0.0	99.71%	CP085798.1
Salmonella enterica subsp. enterica serovar Enteritidis strain Mightyena chromosome, complete genome	1906	1906	100%	0.0	99.71%	CP085792.1
Salmonella enterica subsp. enterica serovar Enteritidis strain Frogadier chromosome, complete genome	1906	1906	100%	0.0	99.71%	CP085795.1
Salmonella enterica subsp. enterica serovar Enteritidis strain Greninja chromosome, complete genome	1906	1906	100%	0.0	99.71%	CP085794.1
Salmonella enterica subsp. enterica serovar Enteritidis strain Poochyena chromosome, complete genome	1906	1906	100%	0.0	99.71%	CP085793.1
Salmonella enterica subsp. enterica serovar Enteritidis strain Froakie chromosome, complete genome	1906	1906	100%	0.0	99.71%	CP085796.1
Salmonella enterica subsp. enterica serovar Enteritidis strain SE211 chromosome, complete genome	1906	1906	100%	0.0	99.71%	CP084532.1
Salmonella enterica subsp. enterica serovar Enteritidis str. CDC_2010K_0968, complete genome	1906	1906	100%	0.0	99.71%	CP007528.1
Salmonella enterica subsp. enterica serovar Enteritidis strain Durban, complete genome	1906	1906	100%	0.0	99.71%	CP007507.1
Salmonella enterica subsp. enterica serovar Enteritidis str. EC20090698, complete genome	1906	1906	100%	0.0	99.71%	CP007248.1
Salmonella enterica subsp. enterica serovar Enteritidis str. EC20110221, complete genome	1906	1906	100%	0.0	99.71%	CP007247.1
Salmonella enterica subsp. enterica serovar Enteritidis str. EC20100101, complete genome	1906	1906	100%	0.0	99.71%	CP007246.1
Salmonella enterica subsp. enterica serovar Enteritidis str. EC20120008, complete genome	1906	1906	100%	0.0	99.71%	CP007245.1
Salmonella enterica subsp. enterica serovar Enteritidis str. EC20110354, complete genome	1906	1906	100%	0.0	99.71%	CP007175.1
Salmonella enterica subsp. enterica serovar Enteritidis str. EC20111174, complete genome	1906	1906	100%	0.0	99.71%	CP007253.1
Salmonella enterica subsp. enterica serovar Enteritidis str. EC20111175, complete genome	1906	1906	100%	0.0	99.71%	CP007252.1
Salmonella enterica subsp. enterica serovar Enteritidis str. EC20110353, complete genome	1906	1906	100%	0.0	99.71%	CP007251.1
Salmonella enterica subsp. enterica serovar Enteritidis str. EC20110355, complete genome	1906	1906	100%	0.0	99.71%	CP007250.1
Salmonella enterica subsp. enterica serovar Enteritidis str. SA19940857 genome	1906	1906	100%	0.0	99.71%	CP007465.1
Salmonella enterica subsp. enterica serovar Enteritidis str. EC20111095, complete genome	1906	1906	100%	0.0	99.71%	CP007254.1

Description	Max Score	Total Score	Query Cover	E value	Per. Ident	Accession
Salmonella enterica subsp. enterica serovar Enteritidis str. EC20110356, complete genome	1906	1906	100%	0.0	99.71%	CP007262.1
Salmonella enterica subsp. enterica serovar Enteritidis str. EC20110357, complete genome	1906	1906	100%	0.0	99.71%	CP007261.1
Salmonella enterica subsp. enterica serovar Enteritidis str. EC20110358, complete genome	1906	1906	100%	0.0	99.71%	CP007260.1
Salmonella enterica subsp. enterica serovar Enteritidis str. EC20110359, complete genome	1906	1906	100%	0.0	99.71%	CP007259.1
Salmonella enterica subsp. enterica serovar Enteritidis str. EC20110360, complete genome	1906	1906	100%	0.0	99.71%	CP007258.1
Salmonella enterica subsp. enterica serovar Enteritidis str. EC20110361, complete genome	1906	1906	100%	0.0	99.71%	CP007263.1
Salmonella enterica subsp. enterica serovar Newlands strain ZC-S1 3rd chromosome, complete genome	1906	1906	100%	0.0	99.71%	CP082916.1
Salmonella enterica subsp. enterica serovar Enteritidis strain CVM N17S192 chromosome, complete genome	1906	1906	100%	0.0	99.71%	CP082726.1
Salmonella enterica subsp. enterica serovar Enteritidis strain CVM N17S111 chromosome, complete genome	1906	1906	100%	0.0	99.71%	CP082729.1
Salmonella enterica strain SLR1_8245 chromosome, complete genome	1906	1906	100%	0.0	99.71%	CP080091.1
Salmonella enterica subsp. enterica serovar Enteritidis strain CFSAN051827 chromosome, complete genome	1906	1906	100%	0.0	99.71%	CP075122.1
Salmonella enterica subsp. enterica serovar Enteritidis strain CFSAN051882 chromosome, complete genome	1906	1906	100%	0.0	99.71%	CP075120.1
Salmonella enterica subsp. enterica serovar Enteritidis strain CFSAN022640 chromosome, complete genome	1906	1906	100%	0.0	99.71%	CP075019.1
Salmonella enterica subsp. enterica serovar Enteritidis strain CFSAN008104 chromosome, complete genome	1906	1906	100%	0.0	99.71%	CP074661.1
Salmonella enterica subsp. enterica serovar Enteritidis strain CFSAN051890 chromosome, complete genome	1906	1906	100%	0.0	99.71%	CP075118.1
Salmonella sp. SJTUF14523 chromosome, complete genome	1906	1906	100%	0.0	99.71%	CP074428.1
Salmonella sp. SJTUF15034 chromosome, complete genome	1906	1906	100%	0.0	99.71%	CP074425.1
Salmonella enterica subsp. enterica serovar Javiana str. CFSAN001992 chromosome, complete genome	1906	1906	100%	0.0	99.71%	CP074314.1
Salmonella enterica subsp. enterica serovar Enteritidis strain CFSAN026631 chromosome, complete genome	1906	1906	100%	0.0	99.71%	CP074254.1
Salmonella enterica subsp. enterica serovar Enteritidis strain CFSAN026633 chromosome, complete genome	1906	1906	100%	0.0	99.71%	CP074252.1
Salmonella enterica subsp. enterica serovar Enteritidis str. 607307-2 chromosome, complete genome	1906	1906	100%	0.0	99.71%	CP074238.1
Salmonella sp. SAL-045 chromosome, complete genome	1906	1906	100%	0.0	99.71%	CP071693.1
Salmonella sp. SAL-007 chromosome, complete genome	1906	1906	100%	0.0	99.71%	CP071686.1
Salmonella sp. SAL-020 chromosome, complete genome	1906	1906	100%	0.0	99.71%	CP071690.1

Description	Max Score	Total Score	Query Cover	E value	Per. Ident	Accession
Salmonella enterica strain Colony118 chromosome	1906	1906	100%	0.0	99.71%	CP070322.1
Salmonella enterica strain Colony582 chromosome	1906	1906	100%	0.0	99.71%	CP070307.1
Salmonella enterica strain Colony116 chromosome	1906	1906	100%	0.0	99.71%	CP070324.1
Salmonella enterica strain Colony117 chromosome	1906	1906	100%	0.0	99.71%	CP070323.1
Salmonella enterica strain Colony114 chromosome	1906	1906	100%	0.0	99.71%	CP070310.1
Salmonella enterica strain FDAARGOS_1066 chromosome, complete genome	1906	1906	100%	0.0	99.71%	CP066047.1
Salmonella enterica subsp. enterica serovar Enteritidis strain P125109 chromosome, complete genome	1906	1906	100%	0.0	99.71%	CP063700.1
Salmonella enterica subsp. enterica serovar Enteritidis strain CP255 chromosome, complete genome	1906	1906	100%	0.0	99.71%	CP063705.1
Salmonella enterica subsp. enterica serovar Enteritidis strain A1636 chromosome, complete genome	1906	1906	100%	0.0	99.71%	CP063708.1
Salmonella enterica subsp. enterica serovar Enteritidis strain D7795 chromosome, complete genome	1906	1906	100%	0.0	99.71%	CP063702.1
Salmonella enterica subsp. enterica serovar Enteritidis strain R17.4111 chromosome, complete genome	1906	1906	100%	0.0	99.71%	CP063289.1
Salmonella enterica strain SLR1_8250 chromosome, complete genome	1906	1906	100%	0.0	99.71%	CP060522.1
Salmonella enterica subsp. enterica serovar Enteritidis str. P125109 complete genome	1906	1906	100%	0.0	99.71%	AM933172.1
Salmonella enterica strain 2010K-2057 chromosome, complete genome	1901	1901	100%	0.0	99.62%	CP053404.1
Salmonella enterica SEHaa3795 DNA, complete genome	1901	1901	100%	0.0	99.62%	AP020330.1
Salmonella enterica subsp. enterica serovar Blockley strain 159838 chromosome, complete genome	1901	1901	100%	0.0	99.62%	CP043662.1
Salmonella enterica subsp. enterica serovar Birkenhead strain AUSMDU00010532 chromosome, complete genome	1901	1901	100%	0.0	99.62%	CP045958.1
Salmonella enterica subsp. enterica strain AD19 chromosome, complete genome	1901	1901	100%	0.0	99.62%	CP043027.1
Salmonella enterica strain CFSAN078398 chromosome, complete genome	1901	1901	100%	0.0	99.62%	CP042443.1
Salmonella enterica subsp. enterica serovar Thompson strain SH11G0791 chromosome, complete genome	1901	1901	100%	0.0	99.62%	CP041171.1
Salmonella enterica subsp. enterica serovar Thompson strain 7 isolate CFSAN047352 chromosome, complete genome	1901	1901	100%	0.0	99.62%	CP040699.1
Salmonella enterica subsp. enterica serovar Typhimurium strain sg_wt7 chromosome, complete genome	1901	1901	100%	0.0	99.62%	CP036168.1
Salmonella enterica subsp. enterica strain NCTC6759 genome assembly, chromosome: 1	1901	1901	100%	0.0	99.62%	LR134146.1
Salmonella enterica subsp. enterica strain NCTC5741 genome assembly, chromosome: 1	1901	1901	100%	0.0	99.62%	LR134232.1
Salmonella enterica strain SA20025921 chromosome, complete genome	1901	1901	100%	0.0	99.62%	CP030214.1

Description	Max Score	Total Score	Query Cover	E value	Per. Ident	Accession
Salmonella enterica subsp. enterica serovar Thompson strain NCTC8496 genome assembly, chromosome: 1	1901	1901	100%	0.0	99.62%	LS483493.1
Salmonella enterica subsp. enterica serovar Thompson strain NCTC5740 genome assembly, chromosome: 1	1901	1901	100%	0.0	99.62%	LS483419.1
Salmonella enterica subsp. enterica serovar Thompson strain HFCDC-SM-846 chromosome, complete genome	1901	1901	100%	0.0	99.62%	CP028729.1
Salmonella enterica strain MFDS1004024 chromosome, complete genome	1901	1901	100%	0.0	99.62%	CP025745.1
Salmonella enterica subsp. enterica serovar Typhimurium strain BL10 chromosome, complete genome	1901	1901	100%	0.0	99.62%	CP024619.1
Salmonella enterica subsp. enterica strain 08-00436 chromosome, complete genome	1901	1901	100%	0.0	99.62%	CP020492.1
Salmonella enterica subsp. enterica serovar Thompson strain CFSAN000738 chromosome, complete genome	1901	1901	100%	0.0	99.62%	CP019196.1
Salmonella enterica subsp. enterica serovar Inverness str. ATCC 10720, complete genome	1901	1901	100%	0.0	99.62%	CP019181.1
Salmonella enterica subsp. enterica serovar Enteritidis str. EC20121744 chromosome	1901	1901	100%	0.0	99.62%	CP007373.2
Salmonella enterica subsp. enterica serovar Enteritidis str. EC20120497 chromosome	1901	1901	100%	0.0	99.62%	CP007351.2
Salmonella enterica subsp. enterica serovar Enteritidis str. EC20120687 genome	1901	1901	100%	0.0	99.62%	CP007341.2
Salmonella enterica subsp. enterica serovar Thompson strain RM1986 chromosome, complete genome	1901	1901	100%	0.0	99.62%	CP012514.1
Salmonella enterica subsp. enterica serovar Thompson strain RM1984 chromosome, complete genome	1901	1901	100%	0.0	99.62%	CP012513.1
Salmonella enterica subsp. enterica serovar Thompson strain MFDS1011716 chromosome, complete genome	1901	1901	100%	0.0	99.62%	CP092690.1
Salmonella enterica subsp. enterica serovar Thompson chromosome, complete genome	1901	1901	100%	0.0	99.62%	CP092510.1
Salmonella enterica subsp. enterica serovar Thompson strain MFDS1011657 chromosome, complete genome	1901	1901	100%	0.0	99.62%	CP092627.1
Salmonella enterica subsp. enterica serovar Bispebjerg strain 20SAL-1342-3 chromosome, complete genome	1901	1901	100%	0.0	99.62%	CP115551.1
Salmonella enterica subsp. enterica serovar Thompson str. ATCC 8391, complete genome	1901	1901	100%	0.0	99.62%	CP011396.1
Salmonella enterica strain 1020677 chromosome, complete genome	1901	1901	100%	0.0	99.62%	CP104643.1
Salmonella enterica strain 1019942 chromosome, complete genome	1901	1901	100%	0.0	99.62%	CP104641.1
Salmonella enterica subsp. enterica strain QA-1986 973 chromosome, complete genome	1901	1901	100%	0.0	99.62%	CP101907.1
Salmonella enterica subsp. enterica strain QA-1986 974 chromosome, complete genome	1901	1901	100%	0.0	99.62%	CP101940.1
Salmonella enterica strain SC2014238 chromosome, complete genome	1901	1901	100%	0.0	99.62%	CP101369.1
Salmonella enterica subsp. enterica serovar Blockley strain R17.0776 chromosome, complete genome	1901	1901	100%	0.0	99.62%	CP100728.1

Description	Max Score	Total Score	Query Cover	E value	Per. Ident	Accession
Salmonella enterica subsp. enterica serovar Blockley strain R18.0186 chromosome, complete genome	1901	1901	100%	0.0	99.62%	CP100710.1
Salmonella enterica subsp. enterica serovar Thompson strain R18.0872 chromosome, complete genome	1901	1901	100%	0.0	99.62%	CP100702.1
Salmonella enterica subsp. enterica serovar Typhimurium strain R17.3867 chromosome, complete genome	1901	1901	100%	0.0	99.62%	CP100691.1
Salmonella enterica strain 628 chromosome, complete genome	1901	1901	100%	0.0	99.62%	CP091550.1
Salmonella enterica strain S639 chromosome, complete genome	1901	1901	100%	0.0	99.62%	CP089207.1
Salmonella enterica subsp. enterica strain 81882 chromosome	1901	1901	100%	0.0	99.62%	CP088146.1
Salmonella enterica subsp. enterica serovar Enteritidis strain Slowpoke chromosome, complete genome	1901	1901	100%	0.0	99.62%	CP085816.1
Salmonella enterica subsp. enterica serovar Enteritidis strain Slowbro chromosome, complete genome	1901	1901	100%	0.0	99.62%	CP085815.1
Salmonella enterica subsp. enterica serovar Enteritidis strain Plusle chromosome, complete genome	1901	1901	100%	0.0	99.62%	CP085814.1
Salmonella enterica subsp. enterica serovar Thompson str. RM6836, complete genome	1901	1901	100%	0.0	99.62%	CP006717.1
Salmonella enterica strain CFSAN033950 chromosome, complete genome	1901	1901	100%	0.0	99.62%	CP075141.1
Salmonella enterica strain CFSAN044885 chromosome, complete genome	1901	1901	100%	0.0	99.62%	CP075137.1
Salmonella enterica subsp. enterica serovar Inverness strain CFSAN044911 chromosome, complete genome	1901	1901	100%	0.0	99.62%	CP075132.1
Salmonella enterica strain CFSAN008789 chromosome, complete genome	1901	1901	100%	0.0	99.62%	CP075050.1
Salmonella enterica strain CFSAN008810 chromosome, complete genome	1901	1901	100%	0.0	99.62%	CP075046.1
Salmonella enterica strain CFSAN008812 chromosome, complete genome	1901	1901	100%	0.0	99.62%	CP075045.1
Salmonella enterica strain CFSAN008813 chromosome, complete genome	1901	1901	100%	0.0	99.62%	CP075044.1
Salmonella enterica strain FDA833961-41 chromosome, complete genome	1901	1901	100%	0.0	99.62%	CP075035.1
Salmonella enterica strain CFSAN008785 chromosome, complete genome	1901	1901	100%	0.0	99.62%	CP074658.1
Salmonella enterica strain CFSAN029868 chromosome, complete genome	1901	1901	100%	0.0	99.62%	CP074639.1
Salmonella enterica strain CFSAN029879 chromosome, complete genome	1901	1901	100%	0.0	99.62%	CP074630.1
Salmonella enterica strain CFSAN029891 chromosome, complete genome	1901	1901	100%	0.0	99.62%	CP074628.1
Salmonella enterica strain CFSAN029895 chromosome, complete genome	1901	1901	100%	0.0	99.62%	CP074626.1
Salmonella enterica subsp. enterica serovar Typhimurium strain SGSC 2187 chromosome, complete genome	1901	1901	100%	0.0	99.62%	CP074615.1
Salmonella enterica subsp. enterica serovar Montevideo str. CFSAN004346 chromosome, complete genome	1901	1901	100%	0.0	99.62%	CP074299.1
Salmonella enterica subsp. enterica serovar Meleagridis str. 0047 chromosome, complete genome	1901	1901	100%	0.0	99.62%	CP074321.1

Description	Max Score	Total Score	Query Cover	E value	Per. Ident	Accession
Salmonella enterica subsp. enterica serovar Thompson str. ATCC 8391 chromosome, complete genome	1901	1901	100%	0.0	99.62%	CP074209.1
Salmonella enterica subsp. enterica serovar Reading strain CFSAN080607 chromosome, complete genome	1901	1901	100%	0.0	99.62%	CP068783.1
Salmonella enterica strain CFSAN076147 chromosome, complete genome	1901	1901	100%	0.0	99.62%	CP068788.1
Salmonella enterica subsp. enterica serovar Reading strain CVM 21978 chromosome	1901	1901	100%	0.0	99.62%	CP051449.1
Salmonella enterica subsp. enterica serovar Reading strain CVM 35189 chromosome, complete genome	1901	1901	100%	0.0	99.62%	CP051307.1
Salmonella enterica strain GSJ/2017-Sal.-009 chromosome, complete genome	1901	1901	100%	0.0	99.62%	CP050833.1
Salmonella enterica subsp. enterica serovar Enteritidis strain 79-2359 chromosome, complete genome	1899	1899	100%	0.0	99.62%	CP018645.1
Salmonella enterica subsp. enterica serovar Enteritidis strain SEE2, complete genome	1899	1899	100%	0.0	99.62%	CP011791.1
Salmonella enterica subsp. enterica serovar Enteritidis strain SEE1, complete genome	1899	1899	100%	0.0	99.62%	CP011790.1
Salmonella enterica strain PNUSAS019263 chromosome, complete genome	1899	1899	100%	0.0	99.62%	CP092318.1
Salmonella enterica strain PNUSAS006351 chromosome, complete genome	1899	1899	100%	0.0	99.62%	CP092329.1
Salmonella enterica strain PNUSAS020468 chromosome, complete genome	1899	1899	100%	0.0	99.62%	CP092311.1
Salmonella enterica strain PNUSAS037276 chromosome, complete genome	1899	1899	100%	0.0	99.62%	CP092307.1
Salmonella enterica strain PNUSAS070846 chromosome, complete genome	1899	1899	100%	0.0	99.62%	CP092304.1
Salmonella enterica strain 2016AM-3354 chromosome, complete genome	1899	1899	100%	0.0	99.62%	CP092290.1
Salmonella enterica SESen3709 DNA, complete genome	1895	1895	100%	0.0	99.52%	AP020332.1
Salmonella enterica subsp. enterica serovar Paratyphi C strain 07-0715 chromosome, complete genome	1895	1895	100%	0.0	99.52%	CP053400.1
Salmonella enterica subsp. enterica serovar Senftenberg strain AR-0405 chromosome, complete genome	1895	1895	100%	0.0	99.52%	CP044203.1
Salmonella enterica strain QH chromosome, complete genome	1895	1895	100%	0.0	99.52%	CP043773.1
Salmonella enterica subsp. enterica serovar Senftenberg str. CFSAN004025 chromosome, complete genome	1895	1895	100%	0.0	99.52%	CP039270.1
Salmonella enterica subsp. enterica serovar Senftenberg strain 11-5006 chromosome, complete genome	1895	1895	100%	0.0	99.52%	CP038601.1
Salmonella enterica subsp. enterica serovar Senftenberg strain SA20061017 chromosome, complete genome	1895	1895	100%	0.0	99.52%	CP038591.1
Salmonella enterica subsp. enterica serovar Senftenberg strain SA20130280 chromosome, complete genome	1895	1895	100%	0.0	99.52%	CP038593.1
Salmonella enterica subsp. enterica serovar Senftenberg strain AR_0127 chromosome, complete genome	1895	1895	100%	0.0	99.52%	CP032194.1
Salmonella sp. SSDFZ54 chromosome, complete genome	1895	1895	100%	0.0	99.52%	CP034819.1
Salmonella enterica subsp. enterica serovar Karamoja strain RSE21 chromosome, complete genome	1895	1895	100%	0.0	99.52%	CP034709.1

Description	Max Score	Total Score	Query Cover	E value	Per. Ident	Accession
Salmonella enterica subsp. enterica serovar Karamoja strain RSE40 chromosome, complete genome	1895	1895	100%	0.0	99.52%	CP034698.1
Salmonella enterica subsp. enterica serovar Senftenberg str. 361154004 chromosome, complete genome	1895	1895	100%	0.0	99.52%	CP029036.1
Salmonella enterica subsp. enterica serovar Senftenberg strain CFSAN047866 chromosome, complete genome	1895	1895	100%	0.0	99.52%	CP029040.1
Salmonella enterica subsp. enterica serovar Senftenberg strain CFSAN045763 chromosome, complete genome	1895	1895	100%	0.0	99.52%	CP029038.1
Salmonella enterica strain CFSAN064034 chromosome, complete genome	1895	1895	100%	0.0	99.52%	CP028169.1
Salmonella enterica subsp. enterica serovar Senftenberg genome assembly NCTC10384, chromosome : 1	1895	1895	100%	0.0	99.52%	LN868943.1
Salmonella enterica strain CVCC 519 chromosome, complete genome	1895	1895	100%	0.0	99.52%	CP110934.1
Salmonella enterica strain XM3104 chromosome, complete genome	1895	1895	100%	0.0	99.52%	CP110931.1
Salmonella enterica subsp. enterica serovar Gallinarum strain SCPM-O-B-4493 chromosome, complete genome	1895	1895	100%	0.0	99.52%	CP088134.1
Salmonella enterica subsp. enterica serovar Choleraesuis strain C500, complete genome	1895	1895	100%	0.0	99.52%	CP007639.1
Salmonella enterica subsp. enterica serovar Enteritidis strain Minun chromosome, complete genome	1895	1895	100%	0.0	99.52%	CP085813.1
Salmonella enterica subsp. enterica serovar Senftenberg strain CVM N18S0991 chromosome, complete genome	1895	1895	100%	0.0	99.52%	CP082574.1
Salmonella enterica subsp. enterica serovar Senftenberg strain FSIS1608545 chromosome, complete genome	1895	1895	100%	0.0	99.52%	CP082434.1
Salmonella enterica strain CFSAN044875 chromosome, complete genome	1895	1895	100%	0.0	99.52%	CP075138.1
Salmonella enterica strain OSF056662 chromosome, complete genome	1895	1895	100%	0.0	99.52%	CP075117.1
Salmonella enterica strain CFSAN008783 chromosome, complete genome	1895	1895	100%	0.0	99.52%	CP074659.1
Salmonella enterica subsp. enterica serovar Choleraesuis strain CFSAN022628 chromosome, complete genome	1895	1895	100%	0.0	99.52%	CP075026.1
Salmonella enterica subsp. enterica serovar Kunzendorf strain CFSAN022631 chromosome, complete genome	1895	1895	100%	0.0	99.52%	CP075024.1
Salmonella enterica subsp. enterica strain CFSAN004085 chromosome, complete genome	1895	1895	100%	0.0	99.52%	CP074310.1
Salmonella enterica subsp. enterica serovar Senftenberg strain CFSAN006211 chromosome, complete genome	1895	1895	100%	0.0	99.52%	CP074285.1
Salmonella enterica subsp. enterica serovar Senftenberg strain CFSAN024719 chromosome, complete genome	1895	1895	100%	0.0	99.52%	CP074261.1
Salmonella enterica subsp. enterica serovar Senftenberg strain CFSAN024722 chromosome, complete genome	1895	1895	100%	0.0	99.52%	CP074260.1
Salmonella enterica subsp. enterica serovar Dessau strain KUFSE-SAL0043 chromosome, complete genome	1895	1895	100%	0.0	99.52%	CP047424.1
Salmonella enterica subsp. enterica serovar Dessau strain KUFSE-SAL0043 chromosome	1895	1895	100%	0.0	99.52%	CP043765.1

Description	Max Score	Total Score	Query Cover	E value	Per. Ident	Accession
Salmonella enterica subsp. enterica serovar Senftenberg strain CVM 20749 chromosome, complete genome	1895	1895	100%	0.0	99.52%	CP051413.1
Salmonella enterica subsp. enterica serovar Saintpaul strain CVM 22543 chromosome, complete genome	1895	1895	100%	0.0	99.52%	CP053055.1
Salmonella enterica subsp. enterica serovar Senftenberg strain CVM 24355 chromosome, complete genome	1895	1895	100%	0.0	99.52%	CP051384.1
Salmonella enterica subsp. enterica serovar Choleraesuis strain CVM 28296 chromosome, complete genome	1895	1895	100%	0.0	99.52%	CP051366.1
Salmonella enterica subsp. enterica serovar Senftenberg strain CVM 34514 chromosome, complete genome	1895	1895	100%	0.0	99.52%	CP051329.1
Salmonella enterica subsp. enterica serovar Westhampton strain CFSAN103867 chromosome, complete genome	1895	1895	100%	0.0	99.52%	CP065859.1
Salmonella enterica subsp. enterica serovar Paratyphi C strain RKS4594, complete genome	1895	1895	100%	0.0	99.52%	CP000857.1
Salmonella enterica subsp. enterica serovar Choleraesuis str. SC-B67 chromosome, complete genome	1895	1895	100%	0.0	99.52%	AE017220.1
Salmonella enterica subsp. enterica serovar Saintpaul strain AUSMDU00010531 chromosome, complete genome	1890	1890	100%	0.0	99.42%	CP045954.1
Salmonella enterica subsp. enterica serovar Saintpaul strain 5 isolate CFSAN047351 chromosome, complete genome	1890	1890	100%	0.0	99.42%	CP040700.1
Salmonella enterica subsp. enterica serovar Typhimurium strain NRRL B-4212 chromosome	1890	1890	100%	0.0	99.42%	CP039757.1
Salmonella enterica subsp. enterica serovar Brancaster strain sg_ww281 chromosome, complete genome	1890	1890	100%	0.0	99.42%	CP036166.1
Salmonella enterica subsp. enterica serovar Choleraesuis str. ATCC 10708, complete genome	1890	1890	100%	0.0	99.42%	CP012344.2
Salmonella enterica subsp. enterica serovar Newport str. CDC 2012K-0938 chromosome, complete genome	1890	1890	100%	0.0	99.42%	CP025246.1
Salmonella enterica subsp. enterica serovar Newport str. CDC 2009K-1331 chromosome, complete genome	1890	1890	100%	0.0	99.42%	CP025248.1
Salmonella enterica subsp. enterica serovar Newport str. USDA-ARS-USMARC-1927, complete genome	1890	1890	100%	0.0	99.42%	CP007216.2
Salmonella enterica subsp. enterica serovar Enteritidis strain 49-2444 chromosome, complete genome	1890	1890	100%	0.0	99.42%	CP018633.1
Salmonella enterica subsp. enterica serovar Ouakam str. SA20034636, complete genome	1890	1890	100%	0.0	99.42%	CP022116.1
Salmonella enterica subsp. enterica serovar Wandsworth str. SA20092095, complete genome	1890	1890	100%	0.0	99.42%	CP019417.1
Salmonella enterica subsp. enterica serovar Bardo strain SA20113257, complete genome	1890	1890	100%	0.0	99.42%	CP019404.1
Salmonella enterica subsp. enterica serovar Saintpaul strain CFSAN004174, complete genome	1890	1890	100%	0.0	99.42%	CP019206.1
Salmonella enterica subsp. enterica serovar Saintpaul strain CFSAN004173, complete genome	1890	1890	100%	0.0	99.42%	CP019204.1

Description	Max Score	Total Score	Query Cover	E value	Per. Ident	Accession
Salmonella enterica subsp. enterica serovar Saintpaul strain CFSAN004175, complete genome	1890	1890	100%	0.0	99.42%	CP019172.1
Salmonella enterica subsp. enterica serovar Newport strain CFSAN001660, complete genome	1890	1890	100%	0.0	99.42%	CP016010.1
Salmonella enterica subsp. enterica serovar Newport str. Levine 15 chromosome, complete genome	1890	1890	100%	0.0	99.42%	CP015924.1
Salmonella enterica subsp. enterica serovar Newport str. Levine 1 chromosome, complete genome	1890	1890	100%	0.0	99.42%	CP015923.1
Salmonella enterica subsp. enterica serovar Ouakam strain GNT-01 genome	1890	5670	100%	0.0	99.42%	CP012038.1
Salmonella enterica strain B-4212 chromosome, complete genome	1890	1890	100%	0.0	99.42%	CP117976.1
Salmonella enterica strain B-4212 chromosome, complete genome	1890	1890	100%	0.0	99.42%	CP117941.1
Salmonella enterica strain SalSpp_sample_05_No.1 chromosome, complete genome	1890	1890	100%	0.0	99.42%	CP104491.1
Salmonella enterica subsp. enterica serovar Gallinarum strain SCPM-O-B-4548 chromosome, complete genome	1890	1890	100%	0.0	99.42%	CP088142.1
Salmonella enterica subsp. enterica serovar Saintpaul strain CVM N18S0870 chromosome, complete genome	1890	1890	100%	0.0	99.42%	CP083383.1
Salmonella enterica strain CFSAN057240 chromosome, complete genome	1890	1890	100%	0.0	99.42%	CP076092.1
Salmonella enterica subsp. enterica serovar Java strain P7704 chromosome	1890	1890	100%	0.0	99.42%	CP065185.1
Salmonella enterica subsp. enterica serovar Newport strain CFSAN022621 chromosome, complete genome	1890	1890	100%	0.0	99.42%	CP075034.1
Salmonella enterica subsp. enterica serovar Newport strain CFSAN022622 chromosome, complete genome	1890	1890	100%	0.0	99.42%	CP075033.1
Salmonella enterica subsp. enterica strain CFSAN002014 chromosome, complete genome	1890	1890	100%	0.0	99.42%	CP074665.1
Salmonella enterica subsp. enterica serovar Newport strain CFSAN028549 chromosome, complete genome	1890	1890	100%	0.0	99.42%	CP074656.1
Salmonella enterica strain CFSAN029927 chromosome, complete genome	1890	1890	100%	0.0	99.42%	CP074625.1
Salmonella enterica subsp. enterica serovar Kunzendorf strain CFSAN022623 chromosome, complete genome	1890	1890	100%	0.0	99.42%	CP075031.1
Salmonella enterica subsp. enterica serovar Newport str. CFSAN000827 chromosome, complete genome	1890	1890	100%	0.0	99.42%	CP074606.1
Salmonella enterica subsp. enterica serovar Paratyphi B str. CFSAN000535 strain SGSC 2233 isolate SARA53 chromosome, complete genome	1890	1890	100%	0.0	99.42%	CP074611.1
Salmonella enterica subsp. enterica serovar Saintpaul strain CFSAN024564 chromosome, complete genome	1890	1890	100%	0.0	99.42%	CP074324.1
Salmonella enterica subsp. enterica strain CFSAN004090 chromosome, complete genome	1890	1890	100%	0.0	99.42%	CP074309.1
Salmonella enterica subsp. enterica strain CFSAN004112 chromosome, complete genome	1890	1890	100%	0.0	99.42%	CP074307.1

Description	Max Score	Total Score	Query Cover	E value	Per. Ident	Accession
Salmonella enterica subsp. enterica serovar Saintpaul strain CFSAN006166 chromosome, complete genome	1890	1890	100%	0.0	99.42%	CP074297.1
Salmonella enterica subsp. enterica serovar Saintpaul strain CFSAN006241 chromosome, complete genome	1890	1890	100%	0.0	99.42%	CP074280.1
Salmonella enterica subsp. enterica serovar Saintpaul strain CFSAN006246 chromosome, complete genome	1890	1890	100%	0.0	99.42%	CP074279.1
Salmonella enterica subsp. enterica serovar Saintpaul strain CFSAN006195 chromosome, complete genome	1890	1890	100%	0.0	99.42%	CP074291.1
Salmonella enterica subsp. enterica serovar Paratyphi B str. CFSAN000545 strain SGSC 2500 isolate SARB43 chromosome, complete genome	1890	1890	100%	0.0	99.42%	CP074225.1
Salmonella enterica subsp. enterica serovar Paratyphi B str. CFSAN000546 strain SGSC 2501 isolate SARB44 chromosome, complete genome	1890	1890	100%	0.0	99.42%	CP074223.1
Salmonella enterica strain FDAARGOS_879 chromosome	1890	1890	100%	0.0	99.42%	CP065890.1
Salmonella enterica subsp. enterica serovar Virchow strain FORC_080 chromosome, complete genome	1884	1884	100%	0.0	99.33%	CP025094.1
Salmonella enterica strain FORC_038 chromosome, complete genome	1884	1884	100%	0.0	99.33%	CP015574.1
Salmonella enterica subsp. enterica serovar Paratyphi B str. SPB7, complete genome	1884	1884	100%	0.0	99.33%	CP000886.1
Salmonella enterica strain 2016K-0213 chromosome, complete genome	1882	1882	100%	0.0	99.33%	CP053402.1
Salmonella enterica subsp. enterica strain NCTC9684 genome assembly, chromosome: 1	1882	1882	100%	0.0	99.23%	LR134233.1
Salmonella enterica subsp. enterica serovar Virchow strain AUSMDU00010533 chromosome, complete genome	1879	1879	100%	0.0	99.23%	CP045945.1
Salmonella enterica subsp. enterica serovar Hadar strain 12-2388 chromosome, complete genome	1879	1879	100%	0.0	99.23%	CP038595.1
Salmonella enterica strain SA20041605 chromosome, complete genome	1879	1879	100%	0.0	99.23%	CP030225.1
Salmonella enterica strain SA20052327 chromosome, complete genome	1879	1879	100%	0.0	99.23%	CP030202.1
Salmonella enterica subsp. enterica serovar Hadar strain FDAARGOS_313 chromosome, complete genome	1879	1879	100%	0.0	99.23%	CP022069.2
Salmonella enterica strain 2013K-1747 chromosome 1, complete sequence	1879	1879	100%	0.0	99.23%	CP111089.1
Salmonella enterica subsp. enterica serovar Hadar strain 2015AM-0414 chromosome, complete genome	1879	1879	100%	0.0	99.23%	CP093120.1
Salmonella enterica strain 2015AM-0511 chromosome, complete genome	1879	1879	100%	0.0	99.23%	CP093140.1
Salmonella enterica strain 2016AM-0673 chromosome, complete genome	1879	1879	100%	0.0	99.23%	CP093116.1
Salmonella enterica strain PNUSAS002131 chromosome, complete genome	1879	1879	100%	0.0	99.23%	CP093112.1
Salmonella enterica strain PNUSAS021403 chromosome, complete genome	1879	1879	100%	0.0	99.23%	CP093103.1
Salmonella enterica subsp. enterica serovar Hadar strain PNUSAS018090 chromosome, complete genome	1879	1879	100%	0.0	99.23%	CP093096.1
Salmonella enterica strain PNUSAS037609 chromosome, complete genome	1879	1879	100%	0.0	99.23%	CP093093.1

Description	Max Score	Total Score	Query Cover	E value	Per. Ident	Accession
Salmonella enterica strain PNUSAS039582 chromosome, complete genome	1879	1879	100%	0.0	99.23%	CP093088.1
Salmonella enterica strain PNUSAS067730 chromosome, complete genome	1879	1879	100%	0.0	99.23%	CP093086.1
Salmonella enterica strain PNUSAS127695 chromosome, complete genome	1879	1879	100%	0.0	99.23%	CP093135.1
Salmonella enterica strain PNUSAS147811 chromosome, complete genome	1879	1879	100%	0.0	99.23%	CP093084.1
Salmonella enterica strain PNUSAS148096 chromosome, complete genome	1879	1879	100%	0.0	99.23%	CP093081.1
Salmonella enterica strain 2016K-0377 chromosome, complete genome	1879	1879	100%	0.0	99.23%	CP093076.1
Salmonella enterica strain PNUSAS074905 chromosome, complete genome	1879	1879	100%	0.0	99.23%	CP093073.1
Salmonella enterica strain 2014AM-2067 chromosome, complete genome	1879	1879	100%	0.0	99.23%	CP093122.1
Salmonella enterica strain 2014AM-1331 chromosome, complete genome	1879	1879	100%	0.0	99.23%	CP093126.1
Salmonella enterica subsp. enterica serovar Hadar strain 2017AM-0493 chromosome, complete genome	1879	1879	100%	0.0	99.23%	CP093109.1
Salmonella enterica subsp. enterica serovar Hadar strain 2021K-0017 chromosome, complete genome	1879	1879	100%	0.0	99.23%	CP093072.1
Salmonella enterica subsp. enterica serovar Hadar strain CVM N18S2154 chromosome, complete genome	1879	1879	100%	0.0	99.23%	CP082531.1
Salmonella enterica subsp. enterica serovar Hadar strain CVM N18S1943 chromosome, complete genome	1879	1879	100%	0.0	99.23%	CP082540.1
Salmonella enterica subsp. enterica serovar Kiambu strain CVM N18S0824 chromosome, complete genome	1879	1879	100%	0.0	99.23%	CP082587.1
Salmonella enterica subsp. enterica serovar Hadar strain CVM N17S312 chromosome, complete genome	1879	1879	100%	0.0	99.23%	CP082720.1
Salmonella enterica subsp. enterica serovar Anatum strain FSIS1710858 chromosome, complete genome	1879	1879	100%	0.0	99.23%	CP082370.1
Salmonella enterica subsp. enterica serovar Hadar strain CVM N17S1270 isolate 17CO10GT07S-B chromosome, complete genome	1879	1879	100%	0.0	99.23%	CP082639.1
Salmonella enterica subsp. enterica serovar Litchfield strain CVM N17S922 isolate 17GA09GT08-S2 chromosome, complete genome	1879	1879	100%	0.0	99.23%	CP082600.1
Salmonella enterica strain CFSAN060808 chromosome, complete genome	1879	1879	100%	0.0	99.23%	CP075108.1
Salmonella enterica subsp. enterica serovar Abortusequi strain CFSAN022626 chromosome, complete genome	1879	1879	100%	0.0	99.23%	CP075029.1
Salmonella enterica subsp. enterica serovar Hadar strain CVM 24392 chromosome, complete genome	1879	1879	100%	0.0	99.23%	CP051370.1
Salmonella enterica subsp. enterica serovar Newport strain VNSEC023 chromosome, complete genome	1879	1879	100%	0.0	99.23%	CP039437.1
Salmonella enterica subsp. enterica serovar Anatum strain Sal-5091 chromosome Sal-5091, complete sequence	1873	1873	100%	0.0	99.14%	CP045518.1
Salmonella enterica subsp. enterica serovar Anatum strain Sal-4737 chromosome Sal-4737, complete sequence	1873	1873	100%	0.0	99.14%	CP045516.1

Description	Max Score	Total Score	Query Cover	E value	Per. Ident	Accession
Salmonella enterica subsp. enterica serovar Anatum strain Sal-4295 chromosome, complete genome	1873	1873	100%	0.0	99.14%	CP045515.1
Salmonella enterica subsp. enterica serovar Anatum strain Sal-3973 chromosome, complete genome	1873	1873	100%	0.0	99.14%	CP045466.1
Salmonella enterica subsp. enterica serovar Anatum strain Sal-3948 chromosome Sal-3948, complete sequence	1873	1873	100%	0.0	99.14%	CP045513.1
Salmonella enterica subsp. enterica serovar Anatum strain Sal-2097 chromosome, complete genome	1873	1873	100%	0.0	99.14%	CP045465.1
Salmonella enterica subsp. enterica serovar Anatum strain Sal-1135 chromosome, complete genome	1873	1873	100%	0.0	99.14%	CP045464.1
Salmonella enterica subsp. enterica serovar Anatum strain M-5360 chromosome M-5360, complete sequence	1873	1873	100%	0.0	99.14%	CP045509.1
Salmonella enterica subsp. enterica serovar Anatum strain M-3851 chromosome, complete genome	1873	1873	100%	0.0	99.14%	CP045461.1
Salmonella enterica subsp. enterica serovar Anatum strain M-3471 chromosome, complete genome	1873	1873	100%	0.0	99.14%	CP045458.1
Salmonella enterica subsp. enterica serovar Anatum str. CFSAN003961 chromosome, complete genome	1873	1873	100%	0.0	99.14%	CP041183.1
Salmonella enterica subsp. enterica serovar Anatum strain CFSAN003959 chromosome, complete genome	1873	1873	100%	0.0	99.14%	CP041184.1
Salmonella enterica subsp. enterica serovar Anatum strain CFSAN076215 chromosome, complete genome	1873	1873	100%	0.0	99.14%	CP033338.1
Salmonella enterica strain SA20101045 chromosome, complete genome	1873	1873	100%	0.0	99.14%	CP030233.1
Salmonella enterica subsp. enterica serovar Anatum strain R16.0676 chromosome, complete genome	1873	1873	100%	0.0	99.14%	CP029800.1
Salmonella enterica subsp. enterica serovar Hayindogo strain CFSAN050752 chromosome, complete genome	1873	1873	100%	0.0	99.14%	CP017719.1
Salmonella enterica subsp. enterica serovar Anatum str. USDA-ARS-USMARC-1765, complete genome	1873	1873	100%	0.0	99.14%	CP014659.2
Salmonella enterica subsp. enterica serovar Anatum str. USDA-ARS-USMARC-1781 chromosome, complete genome	1873	1873	100%	0.0	99.14%	CP014666.2
Salmonella enterica subsp. enterica serovar Anatum str. USDA-ARS-USMARC-1677 chromosome, complete genome	1873	1873	100%	0.0	99.14%	CP014663.2
Salmonella enterica subsp. enterica serovar Anatum str. USDA-ARS-USMARC-1175, complete genome	1873	1873	100%	0.0	99.14%	CP007483.2
Salmonella enterica subsp. enterica serovar Anatum str. USDA-ARS-USMARC-1727, complete genome	1873	1873	100%	0.0	99.14%	CP014621.2
Salmonella enterica subsp. enterica serovar Anatum str. CDC 06-0532, complete genome	1873	1873	100%	0.0	99.14%	CP007211.2
Salmonella enterica subsp. enterica serovar Anatum str. USDA-ARS-USMARC-1735 isolate SAN2113-2 chromosome, complete genome	1873	1873	100%	0.0	99.14%	CP007584.2
Salmonella enterica subsp. enterica serovar Anatum str. USDA-ARS-USMARC-1766 chromosome, complete genome	1873	1873	100%	0.0	99.14%	CP014665.1

Description	Max Score	Total Score	Query Cover	E value	Per. Ident	Accession
Salmonella enterica subsp. enterica serovar Anatum str. USDA-ARS-USMARC-1728 chromosome, complete genome	1873	1873	100%	0.0	99.14%	CP014664.1
Salmonella enterica subsp. enterica serovar Anatum str. USDA-ARS-USMARC-1783 chromosome, complete genome	1873	1873	100%	0.0	99.14%	CP014661.1
Salmonella enterica subsp. enterica serovar Anatum str. USDA-ARS-USMARC-1736 chromosome, complete genome	1873	1873	100%	0.0	99.14%	CP014657.1
Salmonella enterica subsp. enterica serovar Anatum str. USDA-ARS-USMARC-1676 chromosome, complete genome	1873	1873	100%	0.0	99.14%	CP014620.1
Salmonella enterica subsp. enterica serovar Anatum strain GT-38, complete genome	1873	1873	100%	0.0	99.14%	CP013226.1
Salmonella enterica subsp. enterica serovar Anatum strain GT-01, complete genome	1873	1873	100%	0.0	99.14%	CP013222.1
Salmonella enterica subsp. enterica serovar Anatum strain R17.0809 chromosome, complete genome	1873	1873	100%	0.0	99.14%	CP100678.1
Salmonella enterica subsp. enterica serovar Anatum str. ATCC BAA-1592, complete genome	1873	1873	100%	0.0	99.14%	CP007531.1
Salmonella enterica subsp. enterica serovar Hadar strain FSIS11705858 chromosome, complete genome	1873	1873	100%	0.0	99.14%	CP082396.1
Salmonella enterica subsp. enterica strain CFSAN002008 chromosome, complete genome	1873	1873	100%	0.0	99.14%	CP074672.1
Salmonella enterica strain CFSAN064276 chromosome, complete genome	1873	1873	100%	0.0	99.14%	CP075106.1
Salmonella enterica subsp. enterica serovar Anatum strain CFSAN003985 chromosome, complete genome	1873	1873	100%	0.0	99.14%	CP074670.1
Salmonella enterica subsp. enterica serovar Anatum strain CFSAN003983 chromosome, complete genome	1873	1873	100%	0.0	99.14%	CP074313.1
Salmonella enterica subsp. enterica serovar Anatum strain CFSAN006191 chromosome, complete genome	1873	1873	100%	0.0	99.14%	CP074292.1
Salmonella enterica subsp. enterica serovar Anatum strain CFSAN006188 chromosome, complete genome	1873	1873	100%	0.0	99.14%	CP074293.1
Salmonella enterica subsp. enterica serovar Anatum strain CFSAN024763 chromosome, complete genome	1873	1873	100%	0.0	99.14%	CP074259.1
Salmonella enterica subsp. enterica serovar Anatum str. CFSAN000511 strain SGSC 2459 isolate SARB2 chromosome, complete genome	1873	1873	100%	0.0	99.14%	CP074232.1
Salmonella enterica strain Colony627 chromosome	1873	1873	100%	0.0	99.14%	CP070316.1
Salmonella enterica strain CFSAN076166 chromosome, complete genome	1873	1873	100%	0.0	99.14%	CP068784.1
Salmonella enterica subsp. enterica serovar Othmarschen strain CFSAN103872 chromosome, complete genome	1873	1873	100%	0.0	99.14%	CP066260.1
Salmonella enterica subsp. enterica serovar Anatum strain CVM 20746 chromosome	1873	1873	100%	0.0	99.14%	CP051452.1
Salmonella enterica subsp. enterica serovar Anatum strain CVM 22448 chromosome, complete genome	1873	1873	100%	0.0	99.14%	CP051394.1
Salmonella enterica subsp. enterica serovar Anatum strain CVM 33822 chromosome, complete genome	1873	1873	100%	0.0	99.14%	CP051342.1

Description	Max Score	Total Score	Query Cover	E value	Per. Ident	Accession
Salmonella enterica subsp. enterica serovar Newport strain VNSEC031 chromosome, complete genome	1873	1873	100%	0.0	99.14%	CP039436.1
Salmonella enterica strain FDAARGOS_717 chromosome	1868	1868	100%	0.0	99.04%	CP054897.1
Salmonella enterica subsp. enterica strain NCTC8267 genome assembly, chromosome: 1	1868	1868	100%	0.0	99.04%	LR134153.1
Salmonella enterica strain SA20051528 chromosome, complete genome	1868	1868	100%	0.0	99.04%	CP030211.1
Salmonella enterica subsp. enterica serovar Aberdeen strain NCTC5791 genome assembly, chromosome: 1	1868	1868	100%	0.0	99.04%	LS483453.1
Salmonella enterica subsp. enterica serovar Java strain NCTC5706 genome assembly, chromosome: 1	1868	1868	100%	0.0	99.04%	LT571437.1
Salmonella enterica subsp. enterica serovar Liverpool strain Liv11 chromosome	1868	1868	100%	0.0	99.04%	CP083758.1
Salmonella enterica subsp. enterica serovar Abeokuta strain OG19FER4 chromosome, complete genome	1868	1868	100%	0.0	99.04%	CP093445.1
Salmonella enterica subsp. enterica serovar Corvallis strain CFSAN059903 chromosome, complete genome	1868	1868	100%	0.0	99.04%	CP075112.1
Salmonella enterica subsp. enterica serovar Paratyphi B str. CFSAN000541 strain SGSC 2239 isolate SARA59 chromosome, complete genome	1868	1868	100%	0.0	99.04%	CP074609.1
Salmonella enterica subsp. enterica serovar Paratyphi B str. CFSAN000542 strain SGSC 2240 isolate SARA60 chromosome, complete genome	1868	1868	100%	0.0	99.04%	CP074619.1
Salmonella enterica subsp. enterica serovar Nchanga str. CFSAN001091 chromosome, complete genome	1868	1868	100%	0.0	99.04%	CP074323.1
Salmonella enterica subsp. enterica serovar Paratyphi B str. CFSAN000548 strain SGSC 2503 isolate SARB46 chromosome, complete genome	1868	1868	100%	0.0	99.04%	CP074222.1
Salmonella enterica subsp. enterica serovar Hvittingfoss strain AUSMDU00005056 chromosome, complete genome	1862	1862	100%	0.0	98.94%	CP045831.1
Salmonella enterica subsp. enterica strain CFSA231 chromosome, complete genome	1862	1862	100%	0.0	98.94%	CP033350.2
Salmonella enterica subsp. enterica serovar California strain CD-SL01 chromosome, complete genome	1862	1862	100%	0.0	98.94%	CP028900.1
Salmonella enterica subsp. enterica serovar Derby strain Sa64 chromosome, complete genome	1862	1862	100%	0.0	98.94%	CP034250.1
Salmonella enterica subsp. enterica strain NCTC7836 genome assembly, chromosome: 1	1862	1862	100%	0.0	98.94%	LS483490.1
Salmonella enterica subsp. enterica serovar Derby strain 2014LSAL02547 chromosome	1862	1862	100%	0.0	98.94%	CP029486.1
Salmonella enterica subsp. enterica strain DRB_0883TJX21 chromosome	1862	1862	100%	0.0	98.94%	CP076466.1
Salmonella enterica strain S146 chromosome, complete genome	1862	1862	100%	0.0	98.94%	CP077662.1
Salmonella enterica strain S136 chromosome, complete genome	1862	1862	100%	0.0	98.94%	CP077664.1
Salmonella enterica subsp. enterica serovar Derby strain CVM N17S1441 chromosome, complete genome	1862	1862	100%	0.0	98.94%	CP082627.1

Description	Max Score	Total Score	Query Cover	E value	Per. Ident	Accession
Salmonella enterica subsp. enterica serovar Derby strain FSIS1609398 chromosome, complete genome	1862	1862	100%	0.0	98.94%	CP082426.1
Salmonella enterica subsp. enterica serovar Derby strain FSIS11704880 chromosome, complete genome	1862	1862	100%	0.0	98.94%	CP082411.1
Salmonella enterica subsp. enterica serovar Hartford strain CFSAN008725 chromosome, complete genome	1862	1862	100%	0.0	98.94%	CP074660.1
Salmonella enterica subsp. enterica serovar Derby str. 626 chromosome, complete genome	1862	1862	100%	0.0	98.94%	CP074319.1
Salmonella enterica subsp. enterica serovar Hartford strain CFSAN008740 chromosome, complete genome	1862	1862	100%	0.0	98.94%	CP074274.1
Salmonella enterica subsp. enterica serovar Derby strain 14-Sa79 chromosome, complete genome	1862	1862	100%	0.0	98.94%	CP066545.1
Salmonella enterica strain FDAARGOS_711 chromosome	1857	1857	100%	0.0	98.85%	CP055130.1
Salmonella enterica subsp. enterica serovar Typhimurium strain SL7207 chromosome, complete genome	1857	1857	100%	0.0	98.85%	CP053865.1
Salmonella enterica subsp. enterica serovar Typhimurium strain SS2017 chromosome, complete genome	1857	1857	100%	0.0	98.85%	CP053870.1
Salmonella enterica subsp. enterica serovar Typhimurium strain OLF-FSR1_WB_Gull_ST-29 chromosome, complete genome	1857	1857	100%	0.0	98.85%	CP051286.1
Salmonella enterica subsp. enterica serovar Typhimurium strain OLF-FSR1_WB_Gull_ST-32 chromosome, complete genome	1857	1857	100%	0.0	98.85%	CP051284.1
Salmonella enterica subsp. enterica serovar Typhimurium strain OLF-FSR1_WB_Junco_ST-35 chromosome, complete genome	1857	1857	100%	0.0	98.85%	CP051280.1
Salmonella enterica subsp. enterica serovar Typhimurium strain OLF-FSR1_WB_Sparrow_ST-87 chromosome, complete genome	1857	1857	100%	0.0	98.85%	CP051276.1
Salmonella enterica subsp. enterica serovar Worthington strain OLF-FSR1_WB_Partridge_SW-37 chromosome, complete genome	1857	1857	100%	0.0	98.85%	CP051273.1
Salmonella enterica subsp. enterica serovar Typhimurium strain OLF_FSR1_WB_Finch_ST-13 chromosome, complete genome	1857	1857	100%	0.0	98.85%	CP051269.1
Salmonella enterica subsp. enterica serovar Typhimurium strain OLF-FSR1_WB_Hawk_ST-33 chromosome, complete genome	1857	1857	100%	0.0	98.85%	CP051267.1
Salmonella enterica subsp. enterica serovar Typhimurium isolate SV68791 genome assembly, chromosome: 25	1857	1857	100%	0.0	98.85%	LR792461.1
Salmonella enterica subsp. enterica serovar Typhimurium strain ST45 chromosome, complete genome	1857	1857	100%	0.0	98.85%	CP050753.1
Salmonella enterica subsp. enterica serovar Typhimurium strain ST56 chromosome, complete genome	1857	1857	100%	0.0	98.85%	CP050739.1
Salmonella enterica subsp. enterica serovar Typhimurium strain ST106 chromosome, complete genome	1857	1857	100%	0.0	98.85%	CP050728.1

Description	Max Score	Total Score	Query Cover	E value	Per. Ident	Accession
Salmonella enterica subsp. enterica serovar Typhimurium strain ST101 chromosome, complete genome	1857	1857	100%	0.0	98.85%	CP050731.1
Salmonella enterica subsp. enterica serovar Typhimurium strain ST113 chromosome, complete genome	1857	1857	100%	0.0	98.85%	CP050726.1
Salmonella enterica subsp. enterica serovar Typhimurium strain ST53 chromosome, complete genome	1857	1857	100%	0.0	98.85%	CP050745.1
Salmonella enterica subsp. enterica serovar Typhimurium strain ST90 chromosome, complete genome	1857	1857	100%	0.0	98.85%	CP050734.1
Salmonella enterica subsp. enterica serovar Typhimurium strain ST46 chromosome, complete genome	1857	1857	100%	0.0	98.85%	CP050750.1
Salmonella enterica subsp. enterica serovar Typhimurium strain NCCP 16207 chromosome, complete genome	1857	1857	100%	0.0	98.85%	CP041976.1
Salmonella enterica subsp. enterica serovar Typhimurium strain RM13672 chromosome, complete genome	1857	1857	100%	0.0	98.85%	CP047323.1
Salmonella enterica subsp. enterica strain SCSM4.1 chromosome, complete genome	1857	1857	100%	0.0	98.85%	CP047115.1
Salmonella enterica subsp. enterica serovar 4,[5],12:i:- strain PNCS009991 chromosome, complete genome	1857	1857	100%	0.0	98.85%	CP037881.1
Salmonella enterica subsp. enterica serovar 4,[5],12:i:- strain PNCS014863 chromosome, complete genome	1857	1857	100%	0.0	98.85%	CP037879.1
Salmonella enterica subsp. enterica serovar 4,[5],12:i:- strain PNCS015054 chromosome, complete genome	1857	1857	100%	0.0	98.85%	CP037877.1
Salmonella enterica subsp. enterica serovar 4,[5],12:i:- strain PNCS014854 chromosome, complete genome	1857	1857	100%	0.0	98.85%	CP037874.1
Salmonella enterica subsp. enterica serovar 4,[5],12:i:- strain PNCS014875 chromosome, complete genome	1857	1857	100%	0.0	98.85%	CP037882.1
Salmonella enterica subsp. enterica serovar 4,[5],12:i:- strain PNCS009777 chromosome, complete genome	1857	1857	100%	0.0	98.85%	CP036174.1
Salmonella enterica strain FDAARGOS_687 chromosome, complete genome	1857	1857	100%	0.0	98.85%	CP046283.1
Salmonella enterica subsp. enterica serovar Typhimurium strain AUSMDU00008979 chromosome, complete genome	1857	1857	100%	0.0	98.85%	CP045952.1
Salmonella enterica subsp. enterica serovar Typhimurium strain AUSMDU00010529 chromosome, complete genome	1857	1857	100%	0.0	98.85%	CP045949.1
Salmonella enterica subsp. enterica serovar Typhimurium strain AUSMDU00010530 chromosome, complete genome	1857	1857	100%	0.0	98.85%	CP045947.1
Salmonella enterica subsp. enterica serovar Typhimurium strain SO21 chromosome, complete genome	1857	1857	100%	0.0	98.85%	CP032494.1
Salmonella enterica subsp. enterica serovar Typhimurium strain SL26 chromosome, complete genome	1857	1857	100%	0.0	98.85%	CP032490.1
Salmonella enterica subsp. enterica serovar 1,4,[5],12:i:- strain PNCS007098 chromosome, complete genome	1857	1857	100%	0.0	98.85%	CP044967.1
Salmonella enterica subsp. enterica serovar 1,4,[5],12:i:- strain PNCS014881 chromosome, complete genome	1857	1857	100%	0.0	98.85%	CP044961.1

Description	Max Score	Total Score	Query Cover	E value	Per. Ident	Accession
Salmonella enterica subsp. enterica serovar 1,4,[5],12:i:- strain PNCS007087 chromosome, complete genome	1857	1857	100%	0.0	98.85%	CP044957.1
Salmonella enterica strain S61394 chromosome, complete genome	1857	1857	100%	0.0	98.85%	CP035915.1
Salmonella enterica strain S44712 chromosome, complete genome	1857	1857	100%	0.0	98.85%	CP035917.1
Salmonella enterica subsp. enterica serovar Typhimurium strain AR-0408 chromosome	1857	1857	100%	0.0	98.85%	CP044198.1
Salmonella enterica subsp. enterica serovar Albert strain AR-0401 chromosome, complete genome	1857	1857	100%	0.0	98.85%	CP044188.1
Salmonella enterica subsp. enterica serovar Typhimurium str. 14028S substr. JY996 chromosome, complete genome	1857	1857	100%	0.0	98.85%	CP043400.1
Salmonella enterica subsp. enterica serovar Typhimurium str. 14028S substr. GXS275 chromosome, complete genome	1857	1857	100%	0.0	98.85%	CP043399.1
Salmonella enterica subsp. enterica serovar Typhimurium str. 14028S substr. GXS254 chromosome, complete genome	1857	1857	100%	0.0	98.85%	CP043402.1
Salmonella enterica subsp. enterica serovar Typhimurium str. 14028S substr. GXS259 chromosome, complete genome	1857	1857	100%	0.0	98.85%	CP043401.1
Salmonella enterica subsp. enterica strain ST1539 chromosome, complete genome	1857	1857	100%	0.0	98.85%	CP035301.1
Salmonella enterica subsp. enterica serovar 4,[5],12:i:- strain USDA15WA-1 chromosome, complete genome	1857	1857	100%	0.0	98.85%	CP040686.1
Salmonella enterica subsp. enterica serovar 1,4,[5],12:i:- strain SA20143792 chromosome, complete genome	1857	1857	100%	0.0	98.85%	CP041026.1
Salmonella enterica subsp. enterica serovar Typhimurium strain 01ST04081 chromosome, complete genome	1857	1857	100%	0.0	98.85%	CP029840.1
Salmonella enterica subsp. enterica serovar Typhimurium strain 10ST07093 chromosome, complete genome	1857	1857	100%	0.0	98.85%	CP029839.1
Salmonella enterica strain FDAARGOS_768 chromosome, complete genome	1857	1857	100%	0.0	98.85%	CP041005.1
Salmonella enterica subsp. enterica serovar Typhimurium strain SAP18-6199 chromosome, complete genome	1857	1857	100%	0.0	98.85%	CP040900.1
Salmonella enterica subsp. enterica serovar Typhimurium strain 14028 chromosome, complete genome	1857	1857	100%	0.0	98.85%	CP034479.1
Salmonella enterica subsp. enterica serovar 1,4,[5],12:i:- strain SA20082869 chromosome, complete genome	1857	1857	100%	0.0	98.85%	CP040668.1
Salmonella enterica subsp. enterica serovar 1,4,[5],12:i:- strain SA20070548 chromosome, complete genome	1857	1857	100%	0.0	98.85%	CP040651.1
Salmonella enterica subsp. enterica serovar Rough O:-:- strain PNCS009880 chromosome, complete genome	1857	1857	100%	0.0	98.85%	CP040648.1
Salmonella enterica subsp. enterica serovar Typhimurium strain SAP17-8290 chromosome, complete genome	1857	1857	100%	0.0	98.85%	CP040568.1
Salmonella enterica subsp. enterica serovar Typhimurium strain SAP17-7699 chromosome, complete genome	1857	1857	100%	0.0	98.85%	CP040564.1
Salmonella enterica subsp. enterica serovar Typhimurium strain SAP17-7299 chromosome, complete genome	1857	1857	100%	0.0	98.85%	CP040566.1

Description	Max Score	Total Score	Query Cover	E value	Per. Ident	Accession
Salmonella enterica subsp. enterica serovar Typhimurium strain SAP17-7399 chromosome, complete genome	1857	1857	100%	0.0	98.85%	CP040562.1
Salmonella enterica subsp. enterica serovar Typhimurium strain TJWQ005 chromosome, complete genome	1857	1857	100%	0.0	98.85%	CP040458.1
Salmonella enterica subsp. enterica serovar Rough O:-:- strain PNCS009887 chromosome, complete genome	1857	1857	100%	0.0	98.85%	CP040318.1
Salmonella enterica subsp. enterica serovar Typhimurium strain PNCS014879 chromosome, complete genome	1857	1857	100%	0.0	98.85%	CP040321.1
Salmonella enterica subsp. enterica strain CFSA122 chromosome, complete genome	1857	1857	100%	0.0	98.85%	CP033226.2
Salmonella enterica subsp. enterica strain CFSA244 chromosome, complete genome	1857	1857	100%	0.0	98.85%	CP033255.2
Salmonella enterica subsp. enterica strain CFSA12 chromosome, complete genome	1857	1857	100%	0.0	98.85%	CP033257.2
Salmonella enterica subsp. enterica strain CFSA629 chromosome, complete genome	1857	1857	100%	0.0	98.85%	CP033352.2
Salmonella enterica subsp. enterica serovar 1,4,[5],12:i:- strain PNCS014880 chromosome, complete genome	1857	1857	100%	0.0	98.85%	CP039860.1
Salmonella enterica subsp. enterica serovar 1,4,[5],12:i:- strain PNCS014876 chromosome, complete genome	1857	1857	100%	0.0	98.85%	CP039856.1
Salmonella enterica subsp. enterica serovar 1,4,[5],12:i:- strain PNCS014864 chromosome, complete genome	1857	1857	100%	0.0	98.85%	CP039854.1
Salmonella enterica subsp. enterica serovar 1,4,[5],12:i:- strain PNCS014860 chromosome, complete genome	1857	1857	100%	0.0	98.85%	CP039719.1
Salmonella enterica subsp. enterica serovar 1,4,[5],12:i:- strain PNCS000211 chromosome, complete genome	1857	1857	100%	0.0	98.85%	CP039716.1
Salmonella enterica subsp. enterica serovar 1,4,[5],12:i:- strain PNCS014853 chromosome, complete genome	1857	1857	100%	0.0	98.85%	CP039713.1
Salmonella enterica subsp. enterica serovar 1,4,[5],12:i:- strain PNCS014875 chromosome, complete genome	1857	1857	100%	0.0	98.85%	CP039610.1
Salmonella enterica subsp. enterica serovar 1,4,[5],12:i:- strain PNCS014873 chromosome, complete genome	1857	1857	100%	0.0	98.85%	CP039607.1
Salmonella enterica subsp. enterica serovar 1,4,[5],12:i:- strain PNCS014868 chromosome, complete genome	1857	1857	100%	0.0	98.85%	CP039603.1
Salmonella enterica subsp. enterica serovar 1,4,[5],12:i:- strain PNCS014867 chromosome, complete genome	1857	1857	100%	0.0	98.85%	CP039599.1
Salmonella enterica subsp. enterica serovar 1,4,[5],12:i:- strain PNCS014863 chromosome, complete genome	1857	1857	100%	0.0	98.85%	CP039593.1
Salmonella enterica subsp. enterica serovar 1,4,[5],12:i:- strain PNCS014865 chromosome, complete genome	1857	1857	100%	0.0	98.85%	CP039595.1
Salmonella enterica subsp. enterica serovar 1,4,[5],12:i:- strain PNCS014850 chromosome, complete genome	1857	1857	100%	0.0	98.85%	CP039567.1
Salmonella enterica subsp. enterica serovar 1,4,[5],12:i:- strain PNCS014858 chromosome, complete genome	1857	1857	100%	0.0	98.85%	CP039582.1

Description	Max Score	Total Score	Query Cover	E value	Per. Ident	Accession
Salmonella enterica subsp. enterica serovar 1,4,[5],12:i:- strain PNCS014857 chromosome, complete genome	1857	1857	100%	0.0	98.85%	CP039579.1
Salmonella enterica subsp. enterica serovar Typhimurium strain PNCS014862 chromosome, complete genome	1857	1857	100%	0.0	98.85%	CP039591.1
Salmonella enterica subsp. enterica serovar 1,4,[5],12:i:- strain PNCS014852 chromosome, complete genome	1857	1857	100%	0.0	98.85%	CP039569.1
Salmonella enterica subsp. enterica serovar 1,4,[5],12:i:- strain PNCS014861 chromosome, complete genome	1857	1857	100%	0.0	98.85%	CP039588.1
Salmonella enterica subsp. enterica serovar 1,4,[5],12:i:- strain PNCS014848 chromosome, complete genome	1857	1857	100%	0.0	98.85%	CP039564.1
Salmonella enterica subsp. enterica serovar 1,4,[5],12:i:- strain PNCS014855 chromosome, complete genome	1857	1857	100%	0.0	98.85%	CP039572.1
Salmonella enterica subsp. enterica serovar 1,4,[5],12:i:- strain PNCS014847 chromosome, complete genome	1857	1857	100%	0.0	98.85%	CP039561.1
Salmonella enterica subsp. enterica serovar 1,4,[5],12:i:- strain PNCS014849 chromosome, complete genome	1857	1857	100%	0.0	98.85%	CP039565.1
Salmonella enterica subsp. enterica serovar 1,4,[5],12:i:- strain PNCS014859 chromosome, complete genome	1857	1857	100%	0.0	98.85%	CP039585.1
Salmonella enterica subsp. enterica serovar Typhimurium strain PNCS014856 chromosome, complete genome	1857	1857	100%	0.0	98.85%	CP039576.1
Salmonella enterica subsp. enterica serovar 1,4,[5],12:i:- strain PNCS014846 chromosome, complete genome	1857	1857	100%	0.0	98.85%	CP039558.1
Salmonella enterica subsp. enterica serovar 1,4,[5],12:i:- strain PNCS014866 chromosome, complete genome	1857	1857	100%	0.0	98.85%	CP038849.1
Salmonella enterica subsp. enterica serovar Typhimurium strain PNCS014851 chromosome, complete genome	1857	1857	100%	0.0	98.85%	CP038847.1
Salmonella enterica subsp. enterica serovar Typhimurium strain E40V chromosome, complete genome	1857	1857	100%	0.0	98.85%	CP038434.1
Salmonella enterica subsp. enterica serovar Typhimurium strain E40 chromosome, complete genome	1857	1857	100%	0.0	98.85%	CP038432.1
Salmonella enterica subsp. enterica serovar 4,[5],12:i:- L-3841 DNA, complete genome	1857	1857	100%	0.0	98.85%	AP019375.1
Salmonella enterica subsp. enterica serovar 4,[5],12:i:- L-3838 DNA, complete genome	1857	1857	100%	0.0	98.85%	AP019374.1
Salmonella enterica subsp. enterica serovar Typhimurium strain ATCC 14028 chromosome, complete genome	1857	1857	100%	0.0	98.85%	CP034230.1
Salmonella enterica subsp. enterica serovar Typhimurium strain D23580 genome assembly, chromosome: D23580_liv_o	1857	1857	100%	0.0	98.85%	LS997973.1
Salmonella enterica subsp. enterica serovar Typhimurium strain B3589 chromosome, complete genome	1857	1857	100%	0.0	98.85%	CP034968.1
Salmonella enterica subsp. enterica serovar Typhimurium strain FORC_079 chromosome, complete genome	1857	1857	100%	0.0	98.85%	CP025736.1
Salmonella enterica subsp. enterica serovar Typhimurium strain RSE04 chromosome, complete genome	1857	1857	100%	0.0	98.85%	CP034719.1

Description	Max Score	Total Score	Query Cover	E value	Per. Ident	Accession
Salmonella enterica subsp. enterica serovar Typhimurium var. 5- strain CFSAN067216 chromosome, complete genome	1857	1857	100%	0.0	98.85%	CP028318.1
Salmonella enterica subsp. enterica serovar Typhimurium var. 5- strain CFSAN067217 chromosome, complete genome	1857	1857	100%	0.0	98.85%	CP028314.1
Salmonella enterica subsp. enterica strain LSP 389/97 chromosome, complete genome	1857	1857	100%	0.0	98.85%	CP018219.1
Salmonella enterica subsp. enterica serovar Typhimurium strain UGA14 chromosome, complete genome	1857	1857	100%	0.0	98.85%	CP021462.1
Salmonella enterica subsp. enterica serovar Typhimurium strain DA34833 chromosome, complete genome	1857	1857	100%	0.0	98.85%	CP029595.1
Salmonella enterica subsp. enterica serovar Typhimurium strain DA34827 chromosome, complete genome	1857	1857	100%	0.0	98.85%	CP029593.1
Salmonella enterica subsp. enterica serovar Typhimurium strain DA34821 chromosome, complete genome	1857	1857	100%	0.0	98.85%	CP029567.1
Salmonella enterica subsp. enterica serovar Typhimurium strain DA34837 chromosome, complete genome	1857	1857	100%	0.0	98.85%	CP029568.1
Salmonella enterica subsp. enterica serovar Typhimurium strain FDAARGOS_321 chromosome, complete genome	1857	1857	100%	0.0	98.85%	CP022070.2
Salmonella enterica subsp. enterica serovar Typhimurium strain PIR00538 chromosome, complete genome	1857	1857	100%	0.0	98.85%	CP025555.1
Salmonella enterica subsp. enterica serovar Typhimurium strain FDAARGOS_320 chromosome, complete genome	1857	1857	100%	0.0	98.85%	CP027414.1
Salmonella enterica subsp. enterica serovar Typhimurium strain FDAARGOS_317 chromosome, complete genome	1857	1857	100%	0.0	98.85%	CP027410.1
Salmonella enterica subsp. enterica serovar Typhimurium var. 5- strain FDAARGOS_312 chromosome, complete genome	1857	1857	100%	0.0	98.85%	CP022062.2
Salmonella enterica strain LT2 chromosome, complete genome	1857	1857	100%	0.0	98.85%	CP014051.2
Salmonella enterica subsp. enterica serovar Typhimurium strain AR_0031 chromosome, complete genome	1857	1857	100%	0.0	98.85%	CP026700.1
Salmonella enterica subsp. enterica serovar Enteritidis strain 92-0392 chromosome, complete genome	1857	1857	100%	0.0	98.85%	CP018657.1
Salmonella enterica subsp. enterica serovar Typhimurium isolate VNB151-sc-2315230 genome assembly, chromosome: 1	1857	1857	100%	0.0	98.85%	LT795114.1
Salmonella enterica subsp. enterica serovar Typhimurium strain WW012 chromosome, complete genome	1857	1857	100%	0.0	98.85%	CP022168.1
Salmonella enterica subsp. enterica serovar Manhattan strain SA20084699 chromosome, complete genome	1857	1857	100%	0.0	98.85%	CP022497.1
Salmonella enterica subsp. enterica serovar Typhimurium var. monophasic 4,5,12:i:- strain TW-Stm6 chromosome, complete genome	1857	1857	100%	0.0	98.85%	CP019649.1
Salmonella enterica subsp. enterica strain ST1120, complete genome	1857	1857	100%	0.0	98.85%	CP021909.1
Salmonella enterica subsp. enterica strain 16A242, complete genome	1857	1857	100%	0.0	98.85%	CP020922.1

Description	Max Score	Total Score	Query Cover	E value	Per. Ident	Accession
Salmonella enterica subsp. enterica serovar Typhimurium isolate STMU2UK genome assembly, chromosome: 1	1857	1857	100%	0.0	98.85%	LT855376.1
Salmonella enterica strain FORC_030, complete genome	1857	1857	100%	0.0	98.85%	CP015598.1
Salmonella enterica subsp. enterica serovar Typhimurium strain RM10607 chromosome, complete genome	1857	1857	100%	0.0	98.85%	CP013720.1
Salmonella enterica subsp. enterica serovar Typhimurium str. USDA-ARS-USMARC-1899, complete genome	1857	1857	100%	0.0	98.85%	CP007235.2
Salmonella enterica subsp. enterica serovar Typhimurium str. USDA-ARS-USMARC-1898, complete genome	1857	1857	100%	0.0	98.85%	CP014971.2
Salmonella enterica subsp. enterica serovar Typhimurium str. CDC H2662, complete genome	1857	1857	100%	0.0	98.85%	CP014979.2
Salmonella enterica subsp. enterica serovar Typhimurium str. USDA-ARS-USMARC-1810, complete genome	1857	1857	100%	0.0	98.85%	CP014982.2
Salmonella enterica subsp. enterica serovar Typhimurium strain 81741, complete genome	1857	1857	100%	0.0	98.85%	CP019442.1
Salmonella enterica subsp. enterica serovar Cerro strain 87 chromosome, complete genome	1857	1857	100%	0.0	98.85%	CP008925.1
Salmonella enterica subsp. enterica serovar Typhimurium strain RM10961 chromosome, complete genome	1857	1857	100%	0.0	98.85%	CP013702.1
Salmonella enterica subsp. enterica serovar Newport strain CFSAN003387 chromosome, complete genome	1857	1857	100%	0.0	98.85%	CP016014.1
Salmonella enterica subsp. enterica serovar Typhimurium str. SARA13, complete sequence	1857	1857	100%	0.0	98.85%	CP017728.1
Salmonella enterica subsp. enterica serovar Typhimurium strain 13-931, complete genome	1857	1857	100%	0.0	98.85%	CP016385.1
Salmonella enterica subsp. enterica serovar Typhimurium strain NC983 chromosome, complete genome	1857	1857	100%	0.0	98.85%	CP015157.1
Salmonella enterica subsp. enterica strain SA972816 chromosome, complete genome	1857	1857	100%	0.0	98.85%	CP007484.1
Salmonella enterica subsp. enterica serovar Typhimurium str. USDA-ARS-USMARC-1896 chromosome, complete genome	1857	1857	100%	0.0	98.85%	CP014977.1
Salmonella enterica subsp. enterica serovar Typhimurium str. CDC 2009K-1640 chromosome, complete genome	1857	1857	100%	0.0	98.85%	CP014975.1
Salmonella enterica subsp. enterica serovar Typhimurium str. USDA-ARS-USMARC-1808 chromosome, complete genome	1857	1857	100%	0.0	98.85%	CP014969.1
Salmonella enterica subsp. enterica serovar Typhimurium str. CDC 2011K-1702 chromosome, complete genome	1857	1857	100%	0.0	98.85%	CP014967.1
Salmonella enterica subsp. enterica serovar Typhimurium str. CDC 2010K-1587 chromosome, complete genome	1857	1857	100%	0.0	98.85%	CP014965.1
Salmonella enterica subsp. enterica serovar Typhimurium str. CDC 2009K-2059 chromosome, complete genome	1857	1857	100%	0.0	98.85%	CP014983.1
Salmonella enterica subsp. enterica serovar Typhimurium str. USDA-ARS-USMARC-1880 chromosome, complete genome	1857	1857	100%	0.0	98.85%	CP014981.1

Description	Max Score	Total Score	Query Cover	E value	Per. Ident	Accession
Salmonella enterica subsp. enterica serovar Typhimurium strain RM9437, complete genome	1857	1857	100%	0.0	98.85%	CP012985.1
Salmonella enterica subsp. enterica serovar Typhimurium strain SL1344RX genome	1857	1857	100%	0.0	98.85%	CP011233.1
Salmonella enterica subsp. enterica serovar Typhimurium strain YU15, complete genome	1857	1857	100%	0.0	98.85%	CP014358.1
Salmonella enterica subsp. enterica serovar Typhimurium strain SO2, complete genome	1857	1857	100%	0.0	98.85%	CP014356.1
Salmonella enterica subsp. enterica serovar Typhimurium strain SO3, complete genome	1857	1857	100%	0.0	98.85%	CP014536.1
Salmonella enterica subsp. enterica serovar Typhimurium isolate SO4698-09 genome assembly, chromosome: I	1857	1857	100%	0.0	98.85%	LN999997.1
Salmonella enterica subsp. enterica serovar Typhimurium str. LT2, complete genome	1857	1857	100%	0.0	98.85%	AE006468.2
Salmonella enterica subsp. enterica serovar Cerro str. CFSAN001588 chromosome, complete genome	1857	1857	100%	0.0	98.85%	CP012833.1
Salmonella enterica subsp. enterica serovar Typhimurium strain 33676 chromosome, complete genome	1857	1857	100%	0.0	98.85%	CP012681.1
Salmonella enterica subsp. enterica serovar Typhimurium strain ATCC 14028 chromosome, complete genome	1857	1857	100%	0.0	98.85%	CP117244.1
Salmonella enterica subsp. enterica strain 123 chromosome, complete genome	1857	1857	100%	0.0	98.85%	CP117184.1
Salmonella enterica strain PIW95_S14_0299 chromosome, complete genome	1857	1857	100%	0.0	98.85%	CP117033.1
Salmonella enterica subsp. enterica serovar Typhimurium strain S46L1 chromosome, complete genome	1857	1857	100%	0.0	98.85%	CP091540.1
Salmonella enterica subsp. enterica serovar Typhimurium strain S58L2 chromosome, complete genome	1857	1857	100%	0.0	98.85%	CP091542.1
Salmonella enterica subsp. enterica serovar Typhimurium strain R15.0430 chromosome, complete genome	1857	1857	100%	0.0	98.85%	CP115834.1
Salmonella enterica strain 2011K-0052 chromosome	1857	1857	100%	0.0	98.85%	CP114540.1
Salmonella enterica SECerM2017 DNA, complete genome	1857	1857	100%	0.0	98.85%	AP024345.1
Salmonella enterica strain XSK chromosome, complete genome	1857	1857	100%	0.0	98.85%	CP113538.1
Salmonella enterica strain ZLQ chromosome, complete genome	1857	1857	100%	0.0	98.85%	CP113535.1
Salmonella enterica strain ZCX chromosome, complete genome	1857	1857	100%	0.0	98.85%	CP113536.1
Salmonella enterica strain CHC chromosome, complete genome	1857	1857	100%	0.0	98.85%	CP113541.1
Salmonella enterica subsp. enterica strain YU39, complete genome	1857	1857	100%	0.0	98.85%	CP011428.1
Salmonella enterica subsp. enterica serovar Typhimurium str. CDC 2011K-0870, complete genome	1857	1857	100%	0.0	98.85%	CP007523.1
Salmonella enterica subsp. enterica serovar Typhimurium strain MeganVac1 chromosome	1857	1857	100%	0.0	98.85%	CP112994.1
Salmonella enterica subsp. enterica strain S2122 chromosome, complete genome	1857	1857	100%	0.0	98.85%	CP110657.1

Description	Max Score	Total Score	Query Cover	E value	Per. Ident	Accession
Salmonella enterica subsp. enterica serovar Typhimurium strain 1104-65 chromosome, complete genome	1857	1857	100%	0.0	98.85%	CP110201.1
Salmonella enterica subsp. enterica serovar Typhimurium strain 1104-75 chromosome, complete genome	1857	1857	100%	0.0	98.85%	CP110198.1
Salmonella enterica subsp. enterica serovar Paratyphi B strain SZ21B23 chromosome, complete genome	1857	1857	100%	0.0	98.76%	CP107010.1
Salmonella enterica strain SalSpp_sample_07_No.3 chromosome, complete genome	1857	1857	100%	0.0	98.85%	CP104484.1
Salmonella enterica strain PNUSAS048232 chromosome, complete genome	1857	1857	100%	0.0	98.85%	CP104368.1
Salmonella enterica subsp. enterica serovar Typhimurium strain PNUSAS028809 chromosome, complete genome	1857	1857	100%	0.0	98.85%	CP104366.1
Salmonella enterica subsp. enterica serovar Typhimurium strain ATCC 14028 chromosome, complete genome	1857	1857	100%	0.0	98.85%	CP102669.1
Salmonella enterica strain SC2017297 chromosome, complete genome	1857	1857	100%	0.0	98.85%	CP101390.1
Salmonella enterica strain SC2017167 chromosome, complete genome	1857	1857	100%	0.0	98.85%	CP101388.1
Salmonella enterica strain SC2017100 chromosome, complete genome	1857	1857	100%	0.0	98.85%	CP101386.1
Salmonella enterica strain SC2017030 chromosome, complete genome	1857	1857	100%	0.0	98.85%	CP101382.1
Salmonella enterica strain SC2016290 chromosome, complete genome	1857	1857	100%	0.0	98.85%	CP101379.1
Salmonella enterica strain SC2016091 chromosome, complete genome	1857	1857	100%	0.0	98.85%	CP101376.1
Salmonella enterica strain SC2016090 chromosome, complete genome	1857	1857	100%	0.0	98.85%	CP101375.1
Salmonella enterica strain SC2016042 chromosome, complete genome	1857	1857	100%	0.0	98.85%	CP101373.1
Salmonella enterica strain SC2016025 chromosome, complete genome	1857	1857	100%	0.0	98.85%	CP101372.1
Salmonella enterica subsp. enterica serovar Typhimurium strain 013+ chromosome, complete genome	1857	1857	100%	0.0	98.85%	CP099705.1
Salmonella enterica subsp. enterica serovar Typhimurium strain R18.1932 chromosome, complete genome	1857	1857	100%	0.0	98.85%	CP100732.1
Salmonella enterica subsp. enterica serovar Typhimurium strain R18.0409 chromosome, complete genome	1857	1857	100%	0.0	98.85%	CP100707.1
Salmonella enterica subsp. enterica serovar Typhimurium strain R17.5474 chromosome, complete genome	1857	1857	100%	0.0	98.85%	CP100715.1
Salmonella enterica subsp. enterica serovar Typhimurium strain R18.0292 chromosome, complete genome	1857	1857	100%	0.0	98.85%	CP100739.1
Salmonella enterica subsp. enterica serovar Typhimurium strain GD19PS1 chromosome, complete genome	1857	1857	100%	0.0	98.85%	CP098834.1
Salmonella enterica subsp. enterica serovar Typhimurium strain HJL222 chromosome, complete genome	1857	1857	100%	0.0	98.85%	CP098741.1
Salmonella enterica subsp. enterica serovar Typhimurium strain ATOMSal-L6 chromosome, complete genome	1857	1857	100%	0.0	98.85%	CP098438.1
Salmonella enterica subsp. enterica serovar Hissar strain SCPM-O-B-4549 chromosome, complete genome	1857	1857	100%	0.0	98.85%	CP088138.1

Description	Max Score	Total Score	Query Cover	E value	Per. Ident	Accession
Salmonella enterica strain 2 chromosome, complete genome	1857	1857	100%	0.0	98.85%	CP091571.1
Salmonella enterica strain 131 chromosome, complete genome	1857	1857	100%	0.0	98.85%	CP091569.1
Salmonella enterica strain 137 chromosome, complete genome	1857	1857	100%	0.0	98.85%	CP091566.1
Salmonella enterica strain 143 chromosome, complete genome	1857	1857	100%	0.0	98.85%	CP091563.1
Salmonella enterica strain 179 chromosome, complete genome	1857	1857	100%	0.0	98.85%	CP091560.1
Salmonella enterica strain 418 chromosome, complete genome	1857	1857	100%	0.0	98.85%	CP091558.1
Salmonella enterica strain 632 chromosome, complete genome	1857	1857	100%	0.0	98.85%	CP091556.1
Salmonella enterica strain 751 chromosome, complete genome	1857	1857	100%	0.0	98.85%	CP091554.1
Salmonella enterica strain 1559 chromosome, complete genome	1857	1857	100%	0.0	98.85%	CP091547.1
Salmonella enterica strain 1618 chromosome, complete genome	1857	1857	100%	0.0	98.85%	CP091544.1
Salmonella enterica strain 1722 chromosome, complete genome	1857	1857	100%	0.0	98.85%	CP068018.1
Salmonella enterica subsp. enterica strain PartC-Senterica-RM8376 chromosome, complete genome	1857	1857	100%	0.0	98.85%	CP064385.1
Salmonella enterica subsp. enterica strain 3018683606 chromosome, complete genome	1857	1857	100%	0.0	98.85%	CP094332.1
Salmonella enterica strain P048595 chromosome, complete genome	1857	1857	100%	0.0	98.85%	CP093386.1
Salmonella enterica subsp. enterica serovar Typhimurium strain FD01851333 chromosome	1857	1857	100%	0.0	98.85%	CP069164.1
Salmonella enterica subsp. enterica serovar Typhimurium strain NCCP16345 chromosome, complete genome	1857	1857	100%	0.0	98.85%	CP068696.1
Salmonella enterica strain 2016062-SE chromosome, complete genome	1857	1857	100%	0.0	98.85%	CP090539.1
Salmonella enterica strain 2010112-SE chromosome, complete genome	1857	1857	100%	0.0	98.85%	CP090541.1
Salmonella enterica strain 2016089-SE chromosome, complete genome	1857	1857	100%	0.0	98.85%	CP090535.1
Salmonella enterica strain 2017005-SE chromosome, complete genome	1857	1857	100%	0.0	98.85%	CP090533.1
Salmonella enterica strain 2008079-SE chromosome, complete genome	1857	1857	100%	0.0	98.85%	CP090529.1
Salmonella enterica strain 2017028-SE chromosome, complete genome	1857	1857	100%	0.0	98.85%	CP090545.1
Salmonella enterica subsp. enterica serovar Typhimurium strain ZJUJY chromosome, complete genome	1857	1857	100%	0.0	98.85%	CP090304.1
Salmonella enterica subsp. enterica serovar Typhimurium strain SJTUF10330 chromosome, complete genome	1857	1857	100%	0.0	98.85%	CP047542.1
Salmonella enterica subsp. enterica serovar Typhimurium strain SJTUF10057 chromosome, complete genome	1857	1857	100%	0.0	98.85%	CP047550.1
Salmonella enterica subsp. enterica serovar Typhimurium strain SJTUF10112 chromosome, complete genome	1857	1857	100%	0.0	98.85%	CP047555.1
Salmonella enterica subsp. enterica serovar Typhimurium strain SJTUF10169 chromosome, complete genome	1857	1857	100%	0.0	98.85%	CP047548.1

Description	Max Score	Total Score	Query Cover	E value	Per. Ident	Accession
Salmonella enterica subsp. enterica serovar Typhimurium strain SJTUF10231 chromosome, complete genome	1857	1857	100%	0.0	98.85%	CP047553.1
Salmonella enterica subsp. enterica serovar Typhimurium strain SJTUF10236 chromosome, complete genome	1857	1857	100%	0.0	98.85%	CP047546.1
Salmonella enterica subsp. enterica serovar Typhimurium strain SJTUF10250 chromosome, complete genome	1857	1857	100%	0.0	98.85%	CP047544.1
Salmonella enterica subsp. enterica serovar Typhimurium strain SJTUF10359 chromosome, complete genome	1857	1857	100%	0.0	98.85%	CP047540.1
Salmonella enterica subsp. enterica serovar Typhimurium strain SJTUF10405 chromosome, complete genome	1857	1857	100%	0.0	98.85%	CP047537.1
Salmonella enterica subsp. enterica serovar Typhimurium strain SJTUF10484 chromosome, complete genome	1857	1857	100%	0.0	98.85%	CP047531.1
Salmonella enterica subsp. enterica serovar Typhimurium strain SJTUF10640 chromosome, complete genome	1857	1857	100%	0.0	98.85%	CP047529.1
Salmonella enterica subsp. enterica serovar Typhimurium strain SJTUF10648 chromosome, complete genome	1857	1857	100%	0.0	98.85%	CP047527.1
Salmonella enterica subsp. enterica serovar Typhimurium strain SJTUF11077 chromosome, complete genome	1857	1857	100%	0.0	98.85%	CP047525.1
Salmonella enterica subsp. enterica serovar Typhimurium strain SJTUF11216 chromosome, complete genome	1857	1857	100%	0.0	98.85%	CP047522.1
Salmonella enterica subsp. enterica serovar Typhimurium strain ATCC 13311, complete genome	1857	1857	100%	0.0	98.85%	CP009102.1
Salmonella enterica subsp. enterica serovar Typhimurium strain VNP20009, complete genome	1857	1857	100%	0.0	98.85%	CP007804.2
Salmonella enterica strain S15 chromosome, complete genome	1857	1857	100%	0.0	98.85%	CP089209.1
Salmonella enterica strain sg1722-2 chromosome, complete genome	1857	1857	100%	0.0	98.85%	CP081189.2
Salmonella enterica subsp. enterica serovar Typhimurium str. L-3553 DNA, complete genome	1857	1857	100%	0.0	98.85%	AP014565.1
Salmonella enterica subsp. enterica serovar Typhimurium strain S34 chromosome, complete genome	1857	1857	100%	0.0	98.85%	CP086118.1
Salmonella enterica strain SZL 38 chromosome, complete genome	1857	1857	100%	0.0	98.85%	CP085987.1
Salmonella enterica subsp. enterica serovar Typhimurium strain Wartortle chromosome, complete genome	1857	1857	100%	0.0	98.85%	CP085820.1
Salmonella enterica subsp. enterica serovar Typhimurium strain S29 chromosome, complete genome	1857	1857	100%	0.0	98.85%	CP085699.1
Salmonella sp. A39 chromosome, complete genome	1857	1857	100%	0.0	98.85%	CP084194.1
Salmonella enterica subsp. enterica serovar Typhimurium strain 138736, complete genome	1857	1857	100%	0.0	98.85%	CP007581.1
Salmonella enterica strain SP chromosome, complete genome	1857	1857	100%	0.0	98.85%	CP077668.1
Salmonella enterica subsp. enterica serovar Cerro strain FSIS1607502 chromosome, complete genome	1857	1857	100%	0.0	98.85%	CP082902.1

Description	Max Score	Total Score	Query Cover	E value	Per. Ident	Accession
Salmonella enterica subsp. enterica serovar Typhimurium strain CVM N18S2188 chromosome, complete genome	1857	1857	100%	0.0	98.85%	CP082523.1
Salmonella enterica subsp. enterica serovar Typhimurium strain CVM N18S2170 chromosome, complete genome	1857	1857	100%	0.0	98.85%	CP082526.1
Salmonella enterica subsp. enterica serovar Typhimurium strain CVM N18S1677 chromosome, complete genome	1857	1857	100%	0.0	98.85%	CP082543.1
Salmonella enterica subsp. enterica serovar Typhimurium strain CVM N18S1634 chromosome, complete genome	1857	1857	100%	0.0	98.85%	CP082553.1
Salmonella enterica subsp. enterica serovar Typhimurium strain CVM N18S1595 chromosome, complete genome	1857	1857	100%	0.0	98.85%	CP082558.1
Salmonella enterica subsp. enterica serovar 4,[5],12:i:- strain CVM N18S0993 chromosome, complete genome	1857	1857	100%	0.0	98.85%	CP082571.1
Salmonella enterica subsp. enterica serovar Typhimurium strain CVM N18S0981 chromosome, complete genome	1857	1857	100%	0.0	98.85%	CP082577.1
Salmonella enterica subsp. enterica serovar Typhimurium strain CVM N18S0666 chromosome, complete genome	1857	1857	100%	0.0	98.85%	CP082596.1
Salmonella enterica subsp. enterica serovar Typhimurium strain CVM N18S0645 chromosome, complete genome	1857	1857	100%	0.0	98.85%	CP082674.1
Salmonella enterica subsp. enterica serovar Typhimurium strain CVM N18S0597 chromosome, complete genome	1857	1857	100%	0.0	98.85%	CP082681.1
Salmonella enterica subsp. enterica serovar 4,[5],12:i:- strain CVM N18S0173 chromosome, complete genome	1857	1857	100%	0.0	98.85%	CP082696.1
Salmonella enterica subsp. enterica serovar 4,[5],12:i:- strain CVM N17S1465 chromosome, complete genome	1857	1857	100%	0.0	98.85%	CP082624.1
Salmonella enterica subsp. enterica serovar 4,[5],12:i:- strain CVM N17S380 chromosome, complete genome	1857	1857	100%	0.0	98.85%	CP082706.1
Salmonella enterica subsp. enterica serovar Muenchen strain CVM N17S129 chromosome, complete genome	1857	1857	100%	0.0	98.85%	CP082727.1
Salmonella enterica subsp. enterica serovar Typhimurium var. 5- strain CVM N17S016 chromosome, complete genome	1857	1857	100%	0.0	98.85%	CP082670.1
Salmonella enterica subsp. enterica serovar Typhimurium var. 5- strain CVM N16S214 chromosome, complete genome	1857	1857	100%	0.0	98.85%	CP082645.1
Salmonella enterica subsp. enterica serovar 4,[5],12:i:- strain CVM N16S144 chromosome, complete genome	1857	1857	100%	0.0	98.85%	CP082650.1
Salmonella enterica subsp. enterica serovar Typhimurium var. 5- strain CVM N16S189 chromosome, complete genome	1857	1857	100%	0.0	98.85%	CP082647.1
Salmonella enterica subsp. enterica serovar Typhimurium var. 5- strain CVM N16S132 chromosome, complete genome	1857	1857	100%	0.0	98.85%	CP082652.1
Salmonella enterica subsp. enterica serovar 4,[5],12:i:- strain FSIS1702343 chromosome, complete genome	1857	1857	100%	0.0	98.85%	CP082376.1
Salmonella enterica subsp. enterica serovar Typhimurium strain FSIS1702508 chromosome, complete genome	1857	1857	100%	0.0	98.85%	CP082374.1
Salmonella enterica subsp. enterica serovar 4,[5],12:i:- strain FSIS1701601 chromosome, complete genome	1857	1857	100%	0.0	98.85%	CP082384.1

Description	Max Score	Total Score	Query Cover	E value	Per. Ident	Accession
Salmonella enterica subsp. enterica serovar Typhimurium var. 5- strain CVM N16S070 chromosome, complete genome	1857	1857	100%	0.0	98.85%	CP082739.1
Salmonella enterica subsp. enterica serovar Typhimurium var. 5- strain CVM N16S089 chromosome, complete genome	1857	1857	100%	0.0	98.85%	CP082662.1
Salmonella enterica subsp. enterica serovar 4,[5],12:i:- strain FSIS11704067 chromosome, complete genome	1857	1857	100%	0.0	98.85%	CP082417.1
Salmonella enterica subsp. enterica serovar Typhimurium strain FSIS11704063 chromosome, complete genome	1857	1857	100%	0.0	98.85%	CP082421.1
Salmonella enterica subsp. enterica serovar Typhimurium strain FSIS1609224 chromosome, complete genome	1857	1857	100%	0.0	98.85%	CP082429.1
Salmonella enterica subsp. enterica serovar 4,[5],12:i:- strain FSIS1700433 chromosome, complete genome	1857	1857	100%	0.0	98.85%	CP082391.1
Salmonella enterica subsp. enterica serovar Typhimurium strain FSIS1607455 chromosome, complete genome	1857	1857	100%	0.0	98.85%	CP082468.1
Salmonella enterica subsp. enterica serovar 4,[5],12:i:- strain FSIS1607294 chromosome, complete genome	1857	1857	100%	0.0	98.85%	CP082475.1
Salmonella enterica subsp. enterica serovar Typhimurium strain FSIS1607447 chromosome, complete genome	1857	1857	100%	0.0	98.85%	CP082473.1
Salmonella enterica subsp. enterica serovar Muenchen strain FSIS1606504 chromosome, complete genome	1857	1857	100%	0.0	98.85%	CP082506.1
Salmonella enterica subsp. enterica serovar 4,[5],12:i:- strain FSIS1605910 chromosome, complete genome	1857	1857	100%	0.0	98.85%	CP082518.1
Salmonella enterica subsp. enterica serovar Typhimurium strain FSIS1606077 chromosome	1857	1857	100%	0.0	98.85%	CP082515.1
Salmonella enterica subsp. enterica serovar 4,[5],12:i:- strain FSIS1607481 chromosome, complete genome	1857	1857	100%	0.0	98.85%	CP082496.1
Salmonella enterica subsp. enterica serovar 4,[5],12:i:- strain CVM N17S107 isolate 17LA02GT01-S chromosome, complete genome	1857	1857	100%	0.0	98.85%	CP082608.1
Salmonella enterica subsp. enterica serovar Typhimurium strain CVM N17S520 isolate 17GA07GT17-S2 chromosome, complete genome	1857	1857	100%	0.0	98.85%	CP082704.1
Salmonella enterica subsp. enterica serovar Typhimurium strain CVM N17S620 isolate 17KS07GB10-S2 chromosome, complete genome	1857	1857	100%	0.0	98.85%	CP082606.1
Salmonella enterica subsp. enterica strain IBG7b4 chromosome, complete genome	1857	1857	100%	0.0	98.85%	CP082338.1
Salmonella enterica strain sg1722-1 chromosome, complete genome	1857	1857	100%	0.0	98.85%	CP081187.1
Salmonella enterica strain CFSAN057241 chromosome, complete genome	1857	1857	100%	0.0	98.85%	CP076090.1
Salmonella enterica strain CFSAN057258 chromosome, complete genome	1857	1857	100%	0.0	98.85%	CP076086.1
Salmonella enterica subsp. enterica strain CFSAN002003 chromosome, complete genome	1857	1857	100%	0.0	98.85%	CP074673.1
Salmonella enterica strain CFSAN057220 chromosome, complete genome	1857	1857	100%	0.0	98.85%	CP076095.1

Description	Max Score	Total Score	Query Cover	E value	Per. Ident	Accession
Salmonella enterica subsp. enterica strain CFSAN058606 chromosome, complete genome	1857	1857	100%	0.0	98.85%	CP077711.1
Salmonella enterica subsp. enterica serovar Typhimurium strain Colony566 chromosome	1857	1857	100%	0.0	98.85%	CP078531.1
Salmonella enterica subsp. enterica serovar Typhimurium strain Colony560 chromosome	1857	1857	100%	0.0	98.85%	CP078533.1
Salmonella enterica strain Colony628 chromosome	1857	1857	100%	0.0	98.85%	CP078535.1
Salmonella enterica subsp. enterica serovar Typhimurium strain Colony110 chromosome	1857	1857	100%	0.0	98.85%	CP078532.1
Salmonella enterica subsp. enterica serovar Typhimurium str. DT2, complete genome	1857	1857	100%	0.0	98.85%	HG326213.1
Salmonella enterica subsp. enterica serovar Typhimurium DT104 main chromosome, complete genome	1857	1857	100%	0.0	98.85%	HF937208.1
Salmonella enterica subsp. enterica serovar 4,[5],12:i:- str. 08-1736 chromosome, complete genome	1857	1857	100%	0.0	98.85%	CP006602.1
Salmonella enterica subsp. enterica serovar Typhimurium var. 5- str. CFSAN001921, complete genome	1857	1857	100%	0.0	98.85%	CP006048.1
Salmonella enterica strain CFSAN024219 chromosome, complete genome	1857	1857	100%	0.0	98.85%	CP075013.1
Salmonella enterica subsp. enterica strain CFSAN002017 chromosome, complete genome	1857	1857	100%	0.0	98.85%	CP074671.1
Salmonella enterica subsp. enterica serovar Typhimurium strain CFSAN008081 chromosome, complete genome	1857	1857	100%	0.0	98.85%	CP074663.1
Salmonella enterica strain CFSAN029943 chromosome, complete genome	1857	1857	100%	0.0	98.85%	CP074622.1
Salmonella enterica subsp. enterica serovar Typhimurium strain CFSAN018746 chromosome, complete genome	1857	1857	100%	0.0	98.85%	CP028199.2
Salmonella enterica subsp. enterica serovar 4,[5],12:i:- L-4741 DNA, complete genome	1857	1857	100%	0.0	98.85%	AP023319.1
Salmonella enterica subsp. enterica serovar 4,[5],12:i:- L-4681 DNA, complete genome	1857	1857	100%	0.0	98.85%	AP023317.1
Salmonella enterica subsp. enterica serovar 4,[5],12:i:- L-4614 DNA, complete genome	1857	1857	100%	0.0	98.85%	AP023315.1
Salmonella enterica subsp. enterica serovar 4,[5],12:i:- L-4605 DNA, complete genome	1857	1857	100%	0.0	98.85%	AP023313.1
Salmonella enterica subsp. enterica serovar 4,[5],12:i:- L-4596 DNA, complete genome	1857	1857	100%	0.0	98.85%	AP023311.1
Salmonella enterica subsp. enterica serovar 4,[5],12:i:- L-4578 DNA, complete genome	1857	1857	100%	0.0	98.85%	AP023309.1
Salmonella enterica subsp. enterica serovar 4,[5],12:i:- L-4567 DNA, complete genome	1857	1857	100%	0.0	98.85%	AP023306.1
Salmonella enterica subsp. enterica serovar 4,[5],12:i:- L-4551 DNA, complete genome	1857	1857	100%	0.0	98.85%	AP023304.1
Salmonella enterica subsp. enterica serovar 4,[5],12:i:- L-4526 DNA, complete genome	1857	1857	100%	0.0	98.85%	AP023303.1
Salmonella enterica subsp. enterica serovar 4,[5],12:i:- L-4445 DNA, complete genome	1857	1857	100%	0.0	98.85%	AP023300.1
Salmonella enterica subsp. enterica serovar 4,[5],12:i:- L-4334 DNA, complete genome	1857	1857	100%	0.0	98.85%	AP023299.1
Salmonella enterica subsp. enterica serovar 4,[5],12:i:- L-4261 DNA, complete genome	1857	1857	100%	0.0	98.85%	AP023294.1
Salmonella enterica subsp. enterica serovar 4,[5],12:i:- L-4233 DNA, complete genome	1857	1857	100%	0.0	98.85%	AP023292.1

Description	Max Score	Total Score	Query Cover	E value	Per. Ident	Accession
Salmonella enterica subsp. enterica serovar Typhimurium L-4126 DNA, complete genome	1857	1857	100%	0.0	98.85%	AP023291.1
Salmonella enterica subsp. enterica serovar 4,[5],12:i:- L-3844 DNA, complete genome	1857	1857	100%	0.0	98.85%	AP023290.1
Salmonella enterica subsp. enterica serovar 4,[5],12:i:- L-3837 DNA, complete genome	1857	1857	100%	0.0	98.85%	AP023289.1
Salmonella enterica subsp. enterica serovar 4,[5],12:i:- L-3835 DNA, complete genome	1857	1857	100%	0.0	98.85%	AP023288.1
Salmonella enterica strain no75 chromosome, complete genome	1857	1857	100%	0.0	98.85%	CP075372.1
Salmonella enterica strain CFSAN029958 chromosome, complete genome	1857	1857	100%	0.0	98.85%	CP074620.1
Salmonella enterica subsp. enterica serovar Typhimurium strain SGSC 2190 chromosome, complete genome	1857	1857	100%	0.0	98.85%	CP074617.1
Salmonella enterica subsp. enterica serovar Typhimurium str. CFSAN000648 strain SGSC 2189 isolate SARA9 chromosome, complete genome	1857	1857	100%	0.0	98.85%	CP074607.1
Salmonella enterica subsp. enterica serovar Typhimurium strain CFSAN000982 chromosome, complete genome	1857	1857	100%	0.0	98.85%	CP074604.1
Salmonella enterica subsp. enterica serovar Typhimurium strain AUSMDU00007171 genome assembly, chromosome: C1	1857	1857	100%	0.0	98.85%	OU015342.1
Salmonella enterica subsp. enterica serovar Typhimurium strain AUSMDU00027944 genome assembly, chromosome: C1	1857	1857	100%	0.0	98.85%	OU015340.1
Salmonella enterica subsp. enterica serovar Typhimurium strain AUSMDU00018340 genome assembly, chromosome: C1	1857	1857	100%	0.0	98.85%	OU015337.1
Salmonella enterica subsp. enterica serovar Typhimurium strain AUSMDU00005124 genome assembly, chromosome: C1	1857	1857	100%	0.0	98.85%	OU015334.1
Salmonella enterica subsp. enterica serovar Typhimurium strain AUSMDU00027951 genome assembly, chromosome: C1	1857	1857	100%	0.0	98.85%	OU015328.1
Salmonella enterica subsp. enterica serovar Typhimurium strain AUSMDU00005182 genome assembly, chromosome: C1	1857	1857	100%	0.0	98.85%	OU015325.1
Salmonella enterica subsp. enterica serovar Typhimurium strain AUSMDU00004549 genome assembly, chromosome: C1	1857	1857	100%	0.0	98.85%	OU015323.1
Salmonella enterica subsp. enterica serovar Give var. 15 str. CFSAN004343 chromosome, complete genome	1857	1857	100%	0.0	98.85%	CP074305.1
Salmonella enterica subsp. enterica serovar Typhimurium var. 5- strain CFSAN030089 chromosome, complete genome	1857	1857	100%	0.0	98.85%	CP074240.1
Salmonella enterica subsp. enterica serovar Typhimurium strain C_cip chromosome, complete genome	1857	1857	100%	0.0	98.85%	CP074098.1
Salmonella enterica subsp. enterica serovar Typhimurium strain SO_8752_Stm chromosome, complete genome	1857	1857	100%	0.0	98.85%	CP074092.1
Salmonella enterica subsp. enterica serovar Typhimurium strain LB5000 chromosome, complete genome	1857	1857	100%	0.0	98.85%	CP067397.1
Salmonella enterica subsp. enterica serovar Typhimurium strain ER3625 chromosome, complete genome	1857	1857	100%	0.0	98.85%	CP067091.1
Salmonella enterica subsp. enterica serovar Typhimurium str. U288, complete genome	1857	1857	100%	0.0	98.85%	CP003836.1

Description	Max Score	Total Score	Query Cover	E value	Per. Ident	Accession
Salmonella enterica subsp. enterica serovar Typhimurium strain AB42049 chromosome, complete genome	1857	1857	100%	0.0	98.85%	CP064919.1
Salmonella enterica subsp. enterica serovar Typhimurium strain CVM 28321-b chromosome, complete genome	1857	1857	100%	0.0	98.85%	CP051363.1
Salmonella enterica subsp. enterica serovar Typhimurium strain Colony541 chromosome	1857	1857	100%	0.0	98.85%	CP070319.1
Salmonella enterica subsp. enterica serovar Typhimurium strain Colony113 chromosome	1857	1857	100%	0.0	98.85%	CP070321.1
Salmonella enterica subsp. enterica serovar Typhimurium strain ATCC 14028 chromosome, complete genome	1857	1857	100%	0.0	98.85%	CP043907.1
Salmonella enterica subsp. enterica strain Se32 chromosome, complete genome	1857	1857	100%	0.0	98.85%	CP067339.1
Salmonella enterica strain UWI-PS 6 isolate CFSAN103852 chromosome, complete genome	1857	1857	100%	0.0	98.85%	CP066328.1
Salmonella enterica subsp. enterica serovar Muenchen strain CVM 20744 chromosome, complete genome	1857	1857	100%	0.0	98.85%	CP051416.1
Salmonella enterica subsp. enterica serovar Havana strain CVM 20761 chromosome, complete genome	1857	1857	100%	0.0	98.85%	CP051407.1
Salmonella enterica subsp. enterica serovar Typhimurium strain CVM 20762 chromosome, complete genome	1857	1857	100%	0.0	98.85%	CP051404.1
Salmonella enterica subsp. enterica serovar Muenchen strain CVM 22536 chromosome, complete genome	1857	1857	100%	0.0	98.85%	CP051389.1
Salmonella enterica subsp. enterica serovar Typhimurium strain CVM 24350 chromosome, complete genome	1857	1857	100%	0.0	98.85%	CP051386.1
Salmonella enterica subsp. enterica serovar Typhimurium strain CVM 24362 chromosome, complete genome	1857	1857	100%	0.0	98.85%	CP051380.1
Salmonella enterica subsp. enterica serovar Typhimurium strain CVM 28321-a chromosome, complete genome	1857	1857	100%	0.0	98.85%	CP053051.1
Salmonella enterica strain FDAARGOS_1067 chromosome, complete genome	1857	1857	100%	0.0	98.85%	CP066009.1
Salmonella enterica strain FDAARGOS_878 chromosome, complete genome	1857	1857	100%	0.0	98.85%	CP065718.1
Salmonella enterica strain FDAARGOS_928 chromosome, complete genome	1857	1857	100%	0.0	98.85%	CP065639.1
Salmonella enterica subsp. enterica serovar Typhimurium strain R18.1078 chromosome, complete genome	1857	1857	100%	0.0	98.85%	CP065567.1
Salmonella enterica subsp. enterica serovar Typhimurium strain S304 chromosome, complete genome	1857	1857	100%	0.0	98.85%	CP061126.1
Salmonella enterica subsp. enterica serovar Typhimurium strain S438 chromosome, complete genome	1857	1857	100%	0.0	98.85%	CP061124.1
Salmonella enterica subsp. enterica serovar Typhimurium strain S441 chromosome, complete genome	1857	1857	100%	0.0	98.85%	CP061122.1
Salmonella enterica subsp. enterica serovar Typhimurium strain S520 chromosome, complete genome	1857	1857	100%	0.0	98.85%	CP061119.1
Salmonella enterica subsp. enterica serovar Typhimurium strain S585 chromosome, complete genome	1857	1857	100%	0.0	98.85%	CP061115.1

Description	Max Score	Total Score	Query Cover	E value	Per. Ident	Accession
Salmonella enterica subsp. enterica serovar Typhimurium strain LT7 chromosome, complete genome	1857	1857	100%	0.0	98.85%	CP064263.1
Salmonella enterica subsp. enterica serovar Typhimurium strain R17.1451 chromosome, complete genome	1857	1857	100%	0.0	98.85%	CP063294.1
Salmonella enterica subsp. enterica serovar Typhimurium strain BBS1358sml chromosome, complete genome	1857	1857	100%	0.0	98.85%	CP061050.1
Salmonella enterica subsp. enterica serovar Typhimurium strain BBS1358lrg chromosome, complete genome	1857	1857	100%	0.0	98.85%	CP061049.1
Salmonella enterica subsp. enterica serovar Typhimurium strain BBS1359 chromosome, complete genome	1857	1857	100%	0.0	98.85%	CP061047.1
Salmonella enterica subsp. enterica serovar Typhimurium strain BBS1406 chromosome, complete genome	1857	1857	100%	0.0	98.85%	CP061045.1
Salmonella enterica subsp. enterica serovar Typhimurium strain BBS1407 chromosome, complete genome	1857	1857	100%	0.0	98.85%	CP061044.1
Salmonella enterica subsp. enterica serovar Typhimurium isolate 5d8c9f00c6184d00f9da85a6:sample:O1960-05 genome assembly, chromosome: 1	1857	1857	100%	0.0	98.85%	LR861808.1
Salmonella enterica subsp. enterica serovar Typhimurium isolate S.Tm LT2p22_assembled genome assembly, chromosome: 1_Linear-	1857	1857	100%	0.0	98.85%	LR881463.1
Salmonella enterica strain SLR1_8094 chromosome, complete genome	1857	1857	100%	0.0	98.85%	CP060515.1
Salmonella enterica subsp. enterica serovar Typhimurium strain LT2 chromosome, complete genome	1857	1857	100%	0.0	98.85%	CP060507.1
Salmonella enterica subsp. enterica serovar Typhimurium strain BKQZM9 chromosome, complete genome	1857	1857	100%	0.0	98.85%	CP060169.1
Salmonella enterica subsp. enterica serovar Typhimurium strain D37712 chromosome, complete genome	1857	1857	100%	0.0	98.85%	CP060165.1
Salmonella enterica subsp. enterica serovar Typhimurium isolate 5d8c9f00c6184d00f9da85a6:sample:O1960-05 genome assembly, chromosome: 1	1857	3195	100%	0.0	98.85%	LR862421.1
Salmonella enterica subsp. enterica serovar Typhimurium strain SH160 chromosome	1857	1857	100%	0.0	98.85%	CP053294.1
Salmonella enterica subsp. enterica serovar Typhimurium strain GSJ/2017-Sal-008 chromosome, complete genome	1857	1857	100%	0.0	98.85%	CP050130.1
Salmonella enterica strain SRC27 chromosome, complete genome	1857	1857	100%	0.0	98.85%	CP058807.1
Salmonella enterica subsp. enterica serovar Typhimurium str. UK-1, complete genome	1857	1857	100%	0.0	98.85%	CP002614.1
Salmonella enterica subsp. enterica serovar Typhimurium str. ST4/74 chromosome, complete genome	1857	1857	100%	0.0	98.85%	CP002487.1
Salmonella enterica subsp. enterica serovar Typhimurium str. T000240 DNA, complete genome	1857	1857	100%	0.0	98.85%	AP011957.1
Salmonella enterica subsp. enterica serovar Typhimurium SL1344 complete genome	1857	1857	100%	0.0	98.85%	FQ312003.1
Salmonella enterica subsp. enterica serovar Typhimurium str. 14028S chromosome, complete genome	1857	1857	100%	0.0	98.85%	CP001363.1

Description	Max Score	Total Score	Query Cover	E value	Per. Ident	Accession
Salmonella enterica subsp. enterica serovar Typhimurium str. D23580 complete genome	1857	1857	100%	0.0	98.85%	FN424405.1
Salmonella enterica subsp. enterica strain NCTC6754 genome assembly, chromosome: 1	1855	1855	100%	0.0	98.67%	LR134190.1
Salmonella enterica subsp. enterica serovar Concord strain AR-0407 chromosome, complete genome	1851	1851	100%	0.0	98.75%	CP044177.1
Salmonella enterica subsp. enterica serovar Corvallis strain AR-0406 chromosome, complete genome	1851	1851	100%	0.0	98.75%	CP044200.1
Salmonella enterica subsp. enterica serovar Stanley strain AR-0403 chromosome, complete genome	1851	1851	100%	0.0	98.75%	CP044184.1
Salmonella enterica strain CFSAN079107 chromosome, complete genome	1851	1851	100%	0.0	98.75%	CP042440.1
Salmonella enterica subsp. enterica serovar Stanley strain sg_wt8 chromosome, complete genome	1851	1851	100%	0.0	98.75%	CP036167.1
Salmonella enterica subsp. enterica strain NCTC8271 genome assembly, chromosome: 1	1851	1851	100%	0.0	98.75%	LR134148.1
Salmonella enterica strain SA20094620 chromosome, complete genome	1851	1851	100%	0.0	98.75%	CP030185.1
Salmonella enterica subsp. enterica serovar Corvallis strain 12-01738 chromosome, complete genome	1851	1851	100%	0.0	98.75%	CP027677.1
Salmonella enterica subsp. enterica serovar Concord strain CFSAN018747 chromosome, complete genome	1851	1851	100%	0.0	98.75%	CP028196.1
Salmonella enterica subsp. enterica serovar Yovokome str. S-1850, complete genome	1851	1851	100%	0.0	98.75%	CP019418.1
Salmonella enterica strain P164045 chromosome, complete genome	1851	1851	100%	0.0	98.75%	CP092861.1
Salmonella enterica subsp. enterica strain 661 chromosome, complete genome	1851	1851	100%	0.0	98.75%	CP067078.1
Salmonella enterica subsp. enterica serovar Typhimurium strain Blastoise chromosome, complete genome	1851	1851	100%	0.0	98.75%	CP085819.1
Salmonella enterica subsp. enterica serovar Typhimurium var. 5- strain CVM N16S021 chromosome, complete genome	1851	1851	100%	0.0	98.75%	CP082751.1
Salmonella enterica subsp. enterica strain CFSAN058565 chromosome, complete genome	1851	1851	100%	0.0	98.75%	CP077704.1
Salmonella enterica subsp. enterica serovar Paratyphi B strain CFSAN008708 chromosome, complete genome	1851	1851	100%	0.0	98.75%	CP074668.1
Salmonella enterica subsp. enterica serovar Bareilly str. CFSAN000224 chromosome, complete genome	1851	1851	100%	0.0	98.75%	CP074236.1
Salmonella enterica subsp. enterica serovar Typhimurium strain A_cip chromosome, complete genome	1851	1851	100%	0.0	98.75%	CP074094.1
Salmonella enterica strain colony_17 chromosome	1851	1851	100%	0.0	98.75%	CP067991.1
Salmonella enterica subsp. enterica serovar Bareilly strain FC745 chromosome, complete genome	1851	1851	100%	0.0	98.75%	CP063684.2
Salmonella enterica subsp. enterica serovar Typhimurium strain 22792, complete genome	1849	1849	100%	0.0	98.75%	CP017621.1
Salmonella enterica subsp. enterica serovar Typhimurium strain 22495, complete genome	1849	1849	100%	0.0	98.75%	CP017617.1

Description	Max Score	Total Score	Query Cover	E value	Per. Ident	Accession
Salmonella enterica subsp. enterica serovar 4,12:i:- strain CVM N17S056 isolate 17IA01GT15-S H2 chromosome, complete genome	1849	1849	100%	0.0	98.75%	CP082609.1
Salmonella enterica subsp. enterica serovar Stanley strain NCTC5716 genome assembly, chromosome: 1	1845	1845	100%	0.0	98.66%	LS483434.1
Salmonella enterica subsp. enterica serovar Typhimurium strain 2B_cip chromosome, complete genome	1845	1845	100%	0.0	98.66%	CP074096.1
Salmonella enterica subsp. enterica serovar Typhimurium strain AB42052 chromosome, complete genome	1829	1829	100%	0.0	98.37%	CP064918.1
Salmonella enterica subsp. enterica serovar Typhimurium strain AB42142 chromosome, complete genome	1827	1827	100%	0.0	98.37%	CP064916.1
Salmonella enterica subsp. enterica serovar Typhimurium strain FORC098 chromosome, complete genome	1823	1823	100%	0.0	98.27%	CP030029.1
Salmonella enterica subsp. enterica serovar Montevideo strain ATCC 8387 chromosome, complete genome	1823	1823	100%	0.0	98.27%	CP034232.1
Salmonella enterica subsp. enterica serovar Tennessee strain CFSAN076210 chromosome, complete genome	1823	1823	100%	0.0	98.27%	CP033345.1
Salmonella enterica subsp. enterica serovar Tennessee strain PIR00537 chromosome, complete genome	1823	1823	100%	0.0	98.27%	CP025217.1
Salmonella enterica subsp. enterica serovar Tennessee strain ATCC 10722 chromosome, complete genome	1823	1823	100%	0.0	98.27%	CP025218.1
Salmonella enterica subsp. enterica serovar Tennessee strain CFSAN070643 chromosome, complete genome	1823	1823	100%	0.0	98.27%	CP024168.1
Salmonella enterica subsp. enterica serovar Tennessee strain CFSAN070645 chromosome, complete genome	1823	1823	100%	0.0	98.27%	CP024164.1
Salmonella enterica subsp. enterica serovar Tennessee strain CFSAN001387 chromosome, complete genome	1823	1823	100%	0.0	98.27%	CP014994.1
Salmonella enterica subsp. enterica serovar Tennessee strain 21-SA00318-0 chromosome, complete genome	1823	1823	100%	0.0	98.27%	CP091878.1
Salmonella enterica subsp. enterica serovar Tennessee str. TXSC_TXSC08-19, complete genome	1823	1823	100%	0.0	98.27%	CP007505.1
Salmonella enterica strain CFSAN076155 chromosome, complete genome	1823	1823	100%	0.0	98.27%	CP068786.1
Salmonella enterica subsp. enterica serovar Typhimurium str. 798, complete genome	1820	1820	100%	0.0	98.09%	CP003386.1
Salmonella enterica subsp. enterica strain EQAS2016S1 chromosome, complete genome	1818	1818	100%	0.0	98.17%	CP033360.1
Salmonella enterica strain CFSAN064033 chromosome, complete genome	1818	1818	100%	0.0	98.17%	CP028172.1
Salmonella enterica subsp. enterica serovar Bovismorbificans strain SB699 chromosome, complete genome	1818	1818	100%	0.0	98.17%	CP091300.1
Salmonella enterica subsp. enterica serovar Bovismorbificans str. 3114 complete genome	1818	1818	100%	0.0	98.17%	HF969015.2
Salmonella enterica subsp. enterica serovar Bovismorbificans str. Sal610 chromosome	1818	1818	100%	0.0	98.17%	CP076747.1
Salmonella enterica subsp. enterica serovar Bovismorbificans strain 2020LSAL11867 chromosome, complete genome	1818	1818	100%	0.0	98.17%	CP073715.1

Description	Max Score	Total Score	Query Cover	E value	Per. Ident	Accession
Salmonella enterica strain GSJ/2016-Sal.-018 chromosome, complete genome	1818	1818	100%	0.0	98.17%	CP069166.1
Salmonella enterica strain Colony561 chromosome	1818	1818	100%	0.0	98.17%	CP070314.1
Salmonella enterica strain Colony593 chromosome	1818	1818	100%	0.0	98.17%	CP070311.1
Salmonella enterica subsp. enterica serovar Bovismorbificans strain GSJ/2016-Sal-017 chromosome, complete genome	1818	1818	100%	0.0	98.17%	CP069297.1
Salmonella enterica subsp. enterica serovar Bovismorbificans strain CVM 30176 chromosome, complete genome	1818	1818	100%	0.0	98.17%	CP051349.1
Salmonella enterica strain SLR1_7627 chromosome, complete genome	1818	1818	100%	0.0	98.17%	CP060517.1
Salmonella enterica subsp. enterica serovar Typhimurium strain AB42086 chromosome, complete genome	1816	1816	100%	0.0	98.17%	CP064917.1
Salmonella enterica strain FARPER-222 chromosome, complete genome	1816	1816	100%	0.0	98.17%	CP041622.1
Salmonella enterica subsp. enterica serovar Saintpaul strain CVM N16S133 chromosome, complete genome	1812	1812	100%	0.0	98.08%	CP049986.1
Salmonella enterica subsp. enterica serovar Saintpaul strain CVM N40391 chromosome, complete genome	1812	1812	100%	0.0	98.08%	CP049983.1
Salmonella enterica subsp. enterica serovar Saintpaul strain CVM N52030 chromosome, complete genome	1812	1812	100%	0.0	98.08%	CP049981.1
Salmonella enterica subsp. enterica serovar Saintpaul strain NY-N14748 chromosome, complete genome	1812	1812	100%	0.0	98.08%	CP048926.1
Salmonella enterica subsp. enterica serovar Milwaukee str. SA19950795 chromosome, complete genome	1812	1812	100%	0.0	98.08%	CP030175.1
Salmonella enterica subsp. enterica serovar Typhimurium strain FORC58 chromosome, complete genome	1812	1812	100%	0.0	98.08%	CP020565.1
Salmonella enterica subsp. enterica serovar Saintpaul strain SGB23 chromosome, complete genome	1812	1812	100%	0.0	98.08%	CP023166.1
Salmonella enterica subsp. enterica serovar Saintpaul strain SA20031783 chromosome, complete genome	1812	1812	100%	0.0	98.08%	CP022491.1
Salmonella enterica subsp. enterica serovar Typhimurium strain FORC_020, complete genome	1812	1812	100%	0.0	98.08%	CP012144.1
Salmonella enterica strain GSJ/2016-Sal-016 chromosome, complete genome	1812	1812	100%	0.0	98.08%	CP069381.1
Salmonella enterica subsp. enterica serovar Saintpaul strain CVM N18S0175 chromosome, complete genome	1812	1812	100%	0.0	98.08%	CP083385.1
Salmonella enterica subsp. enterica serovar Saintpaul strain S25 chromosome, complete genome	1812	1812	100%	0.0	98.08%	CP085696.1
Salmonella enterica subsp. enterica serovar Newport strain CFSAN024417 chromosome, complete genome	1812	1812	100%	0.0	98.08%	CP074334.1
Salmonella enterica subsp. enterica serovar Newport strain CFSAN024415 chromosome, complete genome	1812	1812	100%	0.0	98.08%	CP074335.1
Salmonella enterica subsp. enterica strain CFSAN004079 chromosome, complete genome	1812	1812	100%	0.0	98.08%	CP074312.1
Salmonella enterica subsp. enterica strain CFSAN004114 chromosome, complete genome	1812	1812	100%	0.0	98.08%	CP074306.1

Description	Max Score	Total Score	Query Cover	E value	Per. Ident	Accession
Salmonella enterica subsp. enterica serovar Saintpaul str. SARA26 strain SGSC 2206 chromosome, complete genome	1812	1812	100%	0.0	98.08%	CP074213.1
Salmonella enterica subsp. enterica serovar Enteritidis strain AUSMDU00010527 chromosome, complete genome	1807	1807	100%	0.0	97.98%	CP045956.1
Salmonella enterica subsp. enterica serovar Infantis strain NCTC6703 genome assembly, chromosome: 1	1807	1807	100%	0.0	97.98%	LS483479.1
Salmonella enterica subsp. enterica serovar Apapa str. SA20060561, complete genome	1807	1807	100%	0.0	97.98%	CP019403.1
Salmonella enterica subsp. enterica serovar Stanleyville str. CFSAN000624 strain SARB61, complete genome	1807	1807	100%	0.0	97.98%	CP017723.1
Salmonella enterica strain CFSAN079104 chromosome, complete genome	1801	1801	100%	0.0	97.89%	CP042441.1
Salmonella enterica subsp. enterica serovar Waycross strain RSE24 chromosome, complete genome	1801	1801	100%	0.0	97.89%	CP034707.1
Salmonella enterica subsp. enterica serovar Saintpaul strain FDAARGOS_373 chromosome, complete genome	1801	1801	100%	0.0	97.89%	CP023512.1
Salmonella enterica subsp. enterica serovar Saintpaul strain CFSAN044909 chromosome, complete genome	1801	1801	100%	0.0	97.89%	CP075134.1
Salmonella enterica strain CFSAN044925 chromosome, complete genome	1801	1801	100%	0.0	97.89%	CP075127.1
Salmonella enterica strain CFSAN029855 chromosome, complete genome	1801	1801	100%	0.0	97.89%	CP074643.1
Salmonella enterica strain CFSAN073553 chromosome, complete genome	1801	1801	100%	0.0	97.89%	CP075103.1
Salmonella enterica strain FDAARGOS_688 chromosome, complete genome	1796	1796	100%	0.0	97.79%	CP046280.1
Salmonella enterica strain MAC15 chromosome, complete genome	1796	1796	100%	0.0	97.79%	CP030749.1
Salmonella enterica subsp. enterica serovar Havana strain NCTC6086 genome assembly, chromosome: 1	1796	1796	100%	0.0	97.79%	LR134187.1
Salmonella enterica subsp. enterica strain NCTC7404 genome assembly, chromosome: 1	1796	1796	100%	0.0	97.79%	LR134144.1
Salmonella enterica subsp. enterica serovar Bareilly strain RSE03 chromosome, complete genome	1796	1796	100%	0.0	97.79%	CP034721.1
Salmonella enterica subsp. enterica serovar 43:a:1,7 strain RSE18 chromosome, complete genome	1796	1796	100%	0.0	97.79%	CP034712.1
Salmonella enterica strain SA20043041 chromosome, complete genome	1796	1796	100%	0.0	97.79%	CP030231.1
Salmonella enterica subsp. enterica serovar Heidelberg strain NCTC5717 genome assembly, chromosome: 1	1796	1796	100%	0.0	97.79%	LS483494.1
Salmonella enterica subsp. enterica serovar Derby isolate 2014LSAL01779 chromosome, complete genome	1796	1796	100%	0.0	97.79%	CP026609.1
Salmonella enterica subsp. enterica serovar Mbandaka str. ATCC 51958 chromosome, complete genome	1796	1796	100%	0.0	97.79%	CP019183.1
Salmonella enterica strain LHICA_E3 chromosome, complete genome	1796	1796	100%	0.0	97.79%	CP079839.1
Salmonella enterica subsp. salamae strain LHICA_SA1 chromosome, complete genome	1796	1796	100%	0.0	97.79%	CP079836.1

Description	Max Score	Total Score	Query Cover	E value	Per. Ident	Accession
Salmonella enterica subsp. salamae strain LHICA_SA3 chromosome, complete genome	1796	1796	100%	0.0	97.79%	CP079838.1
Salmonella enterica subsp. salamae strain CFSAN001015 chromosome, complete genome	1796	1796	100%	0.0	97.79%	CP074596.1
Salmonella enterica subsp. enterica serovar Paratyphi B str. CFSAN000549 strain SGSC 2504 isolate SARB47 chromosome, complete genome	1796	1796	100%	0.0	97.79%	CP074221.1
Salmonella enterica strain 85-0120 chromosome, complete genome	1790	1790	100%	0.0	97.70%	CP054715.1
Salmonella enterica subsp. salamae serovar 58:c:z6 strain 2013K-0366 chromosome, complete genome	1790	1790	100%	0.0	97.69%	CP053326.1
Salmonella enterica subsp. enterica serovar Rissen strain GJ0703-2 chromosome, complete genome	1790	1790	100%	0.0	97.69%	CP043509.1
Salmonella enterica strain FDAARGOS_712 chromosome, complete genome	1790	1790	100%	0.0	97.69%	CP046291.1
Salmonella enterica subsp. enterica serovar Muenster strain PJM1 chromosome, complete genome	1790	1790	100%	0.0	97.70%	CP045038.1
Salmonella enterica subsp. enterica serovar Cubana strain AR-0402 chromosome, complete genome	1790	1790	100%	0.0	97.69%	CP044186.1
Salmonella enterica subsp. enterica strain NCTC7411 genome assembly, chromosome: 1	1790	1790	100%	0.0	97.69%	LR134143.1
Salmonella enterica subsp. enterica serovar 43:a:1,7 strain RSE20 chromosome, complete genome	1790	1790	100%	0.0	97.70%	CP034711.1
Salmonella enterica strain SA20031245 chromosome, complete genome	1790	1790	100%	0.0	97.69%	CP030235.1
Salmonella enterica strain SA20104250 chromosome, complete genome	1790	1790	100%	0.0	97.69%	CP030190.1
Salmonella enterica subsp. salamae serovar Greenside strain NCTC9936 genome assembly, chromosome: 1	1790	1790	100%	0.0	97.69%	LS483475.1
Salmonella enterica subsp. enterica serovar Muenster str. 420 strain CFSAN001301 chromosome, complete genome	1790	1790	100%	0.0	97.70%	CP019201.1
Salmonella enterica subsp. enterica serovar Hvittingfoss str. SA20014981 chromosome, complete genome	1790	1790	100%	0.0	97.69%	CP022503.1
Salmonella enterica subsp. enterica serovar Muenster str. 0315 strain 315, complete genome	1790	1790	100%	0.0	97.70%	CP019198.1
Salmonella enterica strain S1905 chromosome, complete genome	1790	1790	100%	0.0	97.69%	CP103771.1
Salmonella enterica strain SC2014107 chromosome, complete genome	1790	1790	100%	0.0	97.69%	CP101365.1
Salmonella enterica subsp. enterica strain SR33 chromosome, complete genome	1790	1790	100%	0.0	97.69%	CP092911.1
Salmonella enterica subsp. enterica serovar Uganda var. 15+ strain FSIS1607821 chromosome, complete genome	1790	1790	100%	0.0	97.69%	CP082490.1
Salmonella enterica subsp. enterica serovar Muenster strain FSIS1607861 chromosome, complete genome	1790	1790	100%	0.0	97.70%	CP082453.1
Salmonella enterica subsp. enterica serovar Muenster strain FSIS1605743 chromosome, complete genome	1790	1790	100%	0.0	97.70%	CP082486.1
Salmonella enterica strain SCSW714 chromosome, complete genome	1790	1790	100%	0.0	97.69%	CP051213.1

Description	Max Score	Total Score	Query Cover	E value	Per. Ident	Accession
Salmonella enterica subsp. enterica Serovar Cubana str. CFSAN002050, complete genome	1790	1790	100%	0.0	97.69%	CP006055.1
Salmonella enterica subsp. enterica serovar Stanleyville strain CFSAN059881 chromosome, complete genome	1790	1790	100%	0.0	97.69%	CP075116.1
Salmonella enterica strain CFSAN060807 chromosome, complete genome	1790	1790	100%	0.0	97.69%	CP075109.1
Salmonella enterica strain CFSAN024152 chromosome, complete genome	1790	1790	100%	0.0	97.69%	CP075016.1
Salmonella enterica strain CFSAN024184 chromosome, complete genome	1790	1790	100%	0.0	97.69%	CP075015.1
Salmonella enterica subsp. enterica serovar Miami str. 1923 strain SGSC 2485 isolate SARB28 chromosome, complete genome	1790	1790	100%	0.0	97.70%	CP074316.1
Salmonella enterica subsp. enterica serovar Typhimurium var. 5- str. CFSAN004345 chromosome, complete genome	1790	1790	100%	0.0	97.70%	CP074302.1
Salmonella enterica subsp. enterica serovar Uganda strain CFSAN006208 chromosome, complete genome	1790	1790	100%	0.0	97.69%	CP074287.1
Salmonella enterica subsp. enterica serovar Dublin str. CFSAN000517 strain SGSC 2470 isolate SARB13 chromosome, complete genome	1790	1790	100%	0.0	97.69%	CP074226.1
Salmonella enterica subsp. enterica serovar Wien str. CFSAN000656 strain SGSC 2528 isolate SARB71 chromosome, complete genome	1790	1790	100%	0.0	97.69%	CP074212.1
Salmonella enterica subsp. enterica serovar Cubana str. CFSAN001083 chromosome, complete genome	1790	1790	100%	0.0	97.69%	CP074202.1
Salmonella enterica strain Colony596 chromosome	1790	1790	100%	0.0	97.69%	CP070318.1
Salmonella enterica strain Colony589 chromosome	1790	1790	100%	0.0	97.69%	CP070315.1
Salmonella enterica strain Colony598 chromosome	1790	1790	100%	0.0	97.69%	CP070312.1
Salmonella enterica strain Colony568 chromosome	1790	1790	100%	0.0	97.69%	CP070309.1
Salmonella enterica strain Colony539 chromosome	1790	1790	100%	0.0	97.69%	CP070320.1
Salmonella enterica strain Colony564 chromosome	1790	1790	100%	0.0	97.69%	CP070313.1
Salmonella enterica subsp. enterica serovar Uganda strain CVM 20723 chromosome, complete genome	1790	1790	100%	0.0	97.69%	CP051425.1
Salmonella enterica subsp. enterica serovar Uganda strain CVM 22436 chromosome, complete genome	1790	1790	100%	0.0	97.69%	CP051398.1
Salmonella enterica subsp. enterica serovar Uganda strain CVM 22437 chromosome, complete genome	1790	1790	100%	0.0	97.69%	CP051396.1
Salmonella enterica subsp. enterica serovar Muenster strain CVM 22504 chromosome, complete genome	1790	1790	100%	0.0	97.70%	CP051391.1
Salmonella enterica subsp. enterica serovar Muenster strain CVM 34526 chromosome, complete genome	1790	1790	100%	0.0	97.70%	CP051320.1
Salmonella enterica subsp. enterica serovar Uganda strain CVM 35009 chromosome, complete genome	1790	1790	100%	0.0	97.69%	CP051315.1
Salmonella sp. SCFS4 chromosome, complete genome	1784	1784	100%	0.0	97.60%	CP051218.1

Description	Max Score	Total Score	Query Cover	E value	Per. Ident	Accession
Salmonella enterica subsp. enterica serovar Typhimurium isolate SV68221 genome assembly, chromosome: 18	1784	1784	100%	0.0	97.60%	LR792408.1
Salmonella enterica subsp. enterica serovar Indiana strain SI67 chromosome, complete genome	1784	1784	100%	0.0	97.60%	CP050783.1
Salmonella enterica subsp. enterica serovar Indiana strain SI85 chromosome, complete genome	1784	1784	100%	0.0	97.60%	CP050779.1
Salmonella enterica subsp. enterica serovar Indiana strain SI170 chromosome, complete genome	1784	1784	100%	0.0	97.60%	CP050760.1
Salmonella enterica subsp. enterica serovar Indiana strain SI43 chromosome, complete genome	1784	1784	100%	0.0	97.60%	CP050785.1
Salmonella enterica subsp. enterica serovar Indiana strain SI102 chromosome, complete genome	1784	1784	100%	0.0	97.60%	CP050771.1
Salmonella enterica subsp. enterica serovar Indiana strain SI108 chromosome, complete genome	1784	1784	100%	0.0	97.60%	CP050769.1
Salmonella enterica subsp. enterica serovar Indiana strain SI111 chromosome, complete genome	1784	1784	100%	0.0	97.60%	CP050764.1
Salmonella enterica subsp. enterica serovar Indiana strain SI115 chromosome, complete genome	1784	1784	100%	0.0	97.60%	CP050762.1
Salmonella enterica subsp. enterica serovar Indiana strain SI96 chromosome, complete genome	1784	1784	100%	0.0	97.60%	CP050777.1
Salmonella enterica subsp. enterica serovar Indiana strain SI173 chromosome, complete genome	1784	1784	100%	0.0	97.60%	CP050757.1
Salmonella enterica subsp. enterica serovar Indiana strain SI174 chromosome, complete genome	1784	1784	100%	0.0	97.60%	CP050756.1
Salmonella enterica strain FDAARGOS_708 chromosome, complete genome	1784	1784	100%	0.0	97.60%	CP046278.1
Salmonella enterica strain FDAARGOS_707 chromosome, complete genome	1784	1784	100%	0.0	97.60%	CP046279.1
Salmonella enterica subsp. enterica serovar Kentucky strain 161365 chromosome, complete genome	1784	1784	100%	0.0	97.60%	CP043664.1
Salmonella enterica subsp. enterica serovar Kentucky strain 162835 chromosome, complete genome	1784	1784	100%	0.0	97.60%	CP043667.1
Salmonella enterica strain CFSAN096147 chromosome, complete genome	1784	1784	100%	0.0	97.60%	CP044257.1
Salmonella enterica subsp. enterica serovar Indiana strain K16SI097 chromosome	1784	1784	100%	0.0	97.60%	CP043564.1
Salmonella enterica subsp. enterica serovar Indiana strain FJC33 chromosome, complete genome	1784	1784	100%	0.0	97.60%	CP041699.1
Salmonella enterica subsp. enterica serovar Indiana strain SJTUF13520v2 chromosome, complete genome	1784	1784	100%	0.0	97.60%	CP041181.1
Salmonella enterica subsp. enterica serovar Indiana strain SJTUF87912v2 chromosome, complete genome	1784	1784	100%	0.0	97.60%	CP041179.1
Salmonella enterica subsp. enterica serovar Goldcoast strain Sal-5364 chromosome, complete genome	1784	1784	100%	0.0	97.60%	CP039169.1
Salmonella enterica subsp. enterica strain CFSA1096 chromosome, complete genome	1784	1784	100%	0.0	97.60%	CP033348.2

Description	Max Score	Total Score	Query Cover	E value	Per. Ident	Accession
Salmonella enterica subsp. enterica strain CFSA664 chromosome, complete genome	1784	1784	100%	0.0	97.60%	CP033356.2
Salmonella enterica subsp. enterica serovar Indiana strain JT 01 chromosome, complete genome	1784	1784	100%	0.0	97.60%	CP028131.1
Salmonella enterica subsp. enterica serovar Typhimurium strain FORC88 chromosome, complete genome	1784	1784	100%	0.0	97.60%	CP029029.1
Salmonella enterica subsp. enterica serovar Oranienburg strain 12-0523 chromosome, complete genome	1784	1784	100%	0.0	97.60%	CP039277.1
Salmonella enterica subsp. enterica serovar Goldcoast strain R18.0877 chromosome, complete genome	1784	1784	100%	0.0	97.60%	CP037960.1
Salmonella enterica subsp. enterica serovar Kentucky strain K13SK002 chromosome	1784	1784	100%	0.0	97.60%	CP037917.1
Salmonella enterica strain FDA00009424 chromosome, complete genome	1784	1784	100%	0.0	97.60%	CP037891.1
Salmonella enterica subsp. enterica serovar Montevideo str. USDA-ARS-USMARC-1913 chromosome, complete genome	1784	1784	100%	0.0	97.60%	CP025278.1
Salmonella enterica subsp. enterica serovar Goldcoast strain NCTC13175 genome assembly, chromosome: 1	1784	1784	100%	0.0	97.60%	LR134158.1
Salmonella enterica subsp. enterica strain NCTC7831 genome assembly, chromosome: 1	1784	1784	100%	0.0	97.60%	LR134147.1
Salmonella enterica subsp. salamae strain NCTC5773 genome assembly, chromosome: 1	1784	1784	100%	0.0	97.60%	LR134141.1
Salmonella enterica subsp. enterica serovar Senftenberg strain ATCC 8400 chromosome, complete genome	1784	1784	100%	0.0	97.60%	CP034233.1
Salmonella enterica subsp. enterica serovar Mbandaka strain CFSAN076213 chromosome, complete genome	1784	1784	100%	0.0	97.60%	CP033343.1
Salmonella enterica subsp. enterica serovar Oranienburg strain CFSAN076211 chromosome, complete genome	1784	1784	100%	0.0	97.60%	CP033344.1
Salmonella enterica subsp. enterica serovar Lubbock strain 10TTU468x chromosome, complete genome	1784	1784	100%	0.0	97.60%	CP032814.1
Salmonella enterica subsp. enterica serovar Lubbock strain 11TTU1590 chromosome, complete genome	1784	1784	100%	0.0	97.60%	CP032817.1
Salmonella enterica subsp. enterica serovar Mbandaka strain 11TTU1615b chromosome, complete genome	1784	1784	100%	0.0	97.60%	CP032815.1
Salmonella enterica subsp. enterica serovar Dublin strain USMARC-69807 chromosome, complete genome	1784	1784	100%	0.0	97.60%	CP032379.1
Salmonella enterica subsp. enterica serovar Dublin strain USMARC-69840 chromosome, complete genome	1784	1784	100%	0.0	97.60%	CP032446.1
Salmonella enterica subsp. enterica serovar Dublin strain USMARC-69838 chromosome, complete genome	1784	1784	100%	0.0	97.60%	CP032449.1
Salmonella enterica subsp. enterica serovar Dublin strain CVM 22429 chromosome, complete genome	1784	1784	100%	0.0	97.60%	CP032396.1
Salmonella enterica subsp. enterica serovar Dublin strain CVM 34981 chromosome, complete genome	1784	1784	100%	0.0	97.60%	CP032390.1

Description	Max Score	Total Score	Query Cover	E value	Per. Ident	Accession
Salmonella enterica subsp. enterica serovar Dublin strain CVM 22453 chromosome, complete genome	1784	1784	100%	0.0	97.60%	CP032393.1
Salmonella enterica subsp. enterica serovar Dublin strain CVM N45955 chromosome, complete genome	1784	1784	100%	0.0	97.60%	CP032387.1
Salmonella enterica subsp. enterica serovar Dublin strain CVM N53043 chromosome, complete genome	1784	1784	100%	0.0	97.60%	CP032384.1
Salmonella enterica subsp. enterica strain BAA-1672 chromosome, complete genome	1784	1784	100%	0.0	97.60%	CP023470.1
Salmonella sp. SSDFZ69 chromosome, complete genome	1784	1784	100%	0.0	97.60%	CP034831.1
Salmonella enterica subsp. enterica serovar Moero strain RSE29 chromosome, complete genome	1784	1784	100%	0.0	97.60%	CP034705.1
Salmonella enterica subsp. enterica serovar Moero strain RSE08 chromosome, complete genome	1784	1784	100%	0.0	97.60%	CP034718.1
Salmonella enterica subsp. enterica serovar Mikawasima strain RSE15 chromosome, complete genome	1784	1784	100%	0.0	97.60%	CP034713.1
Salmonella enterica subsp. enterica serovar Stanleyville strain RSE10 chromosome, complete genome	1784	1784	100%	0.0	97.60%	CP034716.1
Salmonella enterica subsp. enterica serovar Mikawasima strain RSE13 chromosome, complete genome	1784	1784	100%	0.0	97.60%	CP034715.1
Salmonella enterica subsp. enterica serovar Moero strain RSE02 chromosome, complete genome	1784	1784	100%	0.0	97.60%	CP034722.1
Salmonella enterica subsp. enterica serovar Moero strain RSE28 chromosome, complete genome	1784	1784	100%	0.0	97.60%	CP034706.1
Salmonella enterica subsp. enterica serovar Gaminara str. SA20063285 chromosome, complete genome	1784	1784	100%	0.0	97.60%	CP030288.1
Salmonella enterica subsp. enterica serovar Ohio strain SA20120345 chromosome, complete genome	1784	1784	100%	0.0	97.60%	CP030024.1
Salmonella enterica strain SA20044414 chromosome, complete genome	1784	1784	100%	0.0	97.60%	CP030209.1
Salmonella enterica strain SA19992307 chromosome, complete genome	1784	1784	100%	0.0	97.60%	CP030207.1
Salmonella enterica strain SA20051401 chromosome, complete genome	1784	1784	100%	0.0	97.60%	CP030196.1
Salmonella enterica strain SA20030575 chromosome, complete genome	1784	1784	100%	0.0	97.60%	CP030181.1
Salmonella enterica subsp. salamae serovar 56:b:[1,5] strain SA20053897 chromosome, complete genome	1784	1784	100%	0.0	97.60%	CP029995.1
Salmonella enterica subsp. enterica serovar Poona strain NCTC4840 genome assembly, chromosome: 1	1784	1784	100%	0.0	97.60%	LS483489.1
Salmonella enterica subsp. salamae strain NCTC10310 genome assembly, chromosome: 1	1784	1784	100%	0.0	97.60%	LS483477.1
Salmonella enterica subsp. enterica serovar Senftenberg strain NCTC10080 genome assembly, chromosome: 1	1784	1784	100%	0.0	97.60%	LS483465.1
Salmonella enterica subsp. enterica serovar Kentucky strain 201001922 chromosome	1784	1784	100%	0.0	97.60%	CP028357.1
Salmonella enterica subsp. enterica serovar Kentucky strain PU131 chromosome, complete genome	1784	1784	100%	0.0	97.60%	CP026327.1

Description	Max Score	Total Score	Query Cover	E value	Per. Ident	Accession
Salmonella enterica subsp. enterica serovar Oranienburg str. 0250 strain CFSAN001285 chromosome, complete genome	1784	1784	100%	0.0	97.60%	CP019197.1
Salmonella enterica subsp. enterica serovar Poona str. ATCC BAA-1673 chromosome, complete genome	1784	1784	100%	0.0	97.60%	CP019189.1
Salmonella enterica subsp. enterica serovar Pomona str. ATCC 10729, complete sequence	1784	1784	100%	0.0	97.60%	CP019186.1
Salmonella enterica subsp. enterica serovar Paratyphi A strain FDAARGOS_368 chromosome, complete genome	1784	1784	100%	0.0	97.60%	CP023508.1
Salmonella enterica subsp. enterica serovar Mbandaka strain SA20026234 chromosome, complete genome	1784	1784	100%	0.0	97.60%	CP022489.1
Salmonella enterica subsp. enterica serovar Indiana strain D90, complete genome	1784	1784	100%	0.0	97.60%	CP022450.1
Salmonella enterica subsp. enterica serovar Minnesota strain CFSAN017963, complete genome	1784	1784	100%	0.0	97.60%	CP017720.1
Salmonella enterica subsp. enterica strain 2012K-0678, genome	1784	1784	100%	0.0	97.60%	CP020718.1
Salmonella enterica subsp. enterica serovar Manchester str. ST278, complete genome	1784	1784	100%	0.0	97.60%	CP019414.1
Salmonella enterica subsp. enterica serovar Paratyphi A str. ATCC 11511 chromosome, complete genome	1784	1784	100%	0.0	97.60%	CP019185.1
Salmonella enterica subsp. enterica serovar Rubislaw str. ATCC 10717, complete genome	1784	1784	100%	0.0	97.60%	CP019192.1
Salmonella enterica subsp. enterica serovar Minnesota str. ATCC 49284 chromosome, complete genome	1784	1784	100%	0.0	97.60%	CP019184.1
Salmonella enterica subsp. enterica serovar Dublin str. ATCC 39184 chromosome, complete genome	1784	1784	100%	0.0	97.60%	CP019179.1
Salmonella enterica subsp. enterica serovar Saintpaul str. SARA26, complete genome	1784	1784	100%	0.0	97.60%	CP017727.1
Salmonella enterica strain C629, complete genome	1784	1784	100%	0.0	97.60%	CP015724.1
Salmonella enterica subsp. enterica serovar Mbandaka strain SM_F28R chromosome, complete genome	1784	1784	100%	0.0	97.60%	CP117852.1
Salmonella enterica subsp. enterica serovar Mbandaka strain SM_B30R chromosome, complete genome	1784	1784	100%	0.0	97.60%	CP117851.1
Salmonella enterica subsp. enterica serovar Mbandaka strain SM_F22R chromosome, complete genome	1784	1784	100%	0.0	97.60%	CP117850.1
Salmonella enterica subsp. enterica serovar London strain L1 chromosome, complete genome	1784	1784	100%	0.0	97.60%	CP117698.1
Salmonella enterica subsp. enterica serovar London strain S1 chromosome, complete genome	1784	1784	100%	0.0	97.60%	CP117693.1
Salmonella enterica subsp. enterica serovar London strain F1 chromosome, complete genome	1784	1784	100%	0.0	97.60%	CP117696.1
Salmonella enterica subsp. enterica serovar Mbandaka strain SM-F22S chromosome, complete genome	1784	1784	100%	0.0	97.60%	CP117299.1
Salmonella enterica subsp. enterica serovar Mbandaka strain SM_F28S chromosome, complete genome	1784	1784	100%	0.0	97.60%	CP117181.1
Salmonella enterica subsp. enterica serovar Paratyphi A strain CMCC50093 genome	1784	1784	100%	0.0	97.60%	CP011967.1

Description	Max Score	Total Score	Query Cover	E value	Per. Ident	Accession
Salmonella enterica strain SSJHZ21-044 chromosome, complete genome	1784	1784	100%	0.0	97.60%	CP116018.1
Salmonella enterica subsp. enterica serovar Oranienburg 11548 DNA, complete genome	1784	1784	100%	0.0	97.60%	AP026680.1
Salmonella enterica strain 965 chromosome, complete genome	1784	1784	100%	0.0	97.60%	CP091640.1
Salmonella enterica strain 1063 chromosome, complete genome	1784	1784	100%	0.0	97.60%	CP091636.1
Salmonella enterica strain 1116 chromosome, complete genome	1784	1784	100%	0.0	97.60%	CP091634.1
Salmonella enterica strain 1147 chromosome, complete genome	1784	1784	100%	0.0	97.60%	CP091630.1
Salmonella enterica strain 1165 chromosome, complete genome	1784	1784	100%	0.0	97.60%	CP091626.1
Salmonella enterica strain 1382 chromosome, complete genome	1784	1784	100%	0.0	97.60%	CP091624.1
Salmonella enterica strain 1419 chromosome, complete genome	1784	1784	100%	0.0	97.60%	CP091622.1
Salmonella enterica strain 1473 chromosome, complete genome	1784	1784	100%	0.0	97.60%	CP091619.1
Salmonella enterica strain 1653 chromosome, complete genome	1784	1784	100%	0.0	97.60%	CP091615.1
Salmonella enterica strain 1795 chromosome, complete genome	1784	1784	100%	0.0	97.60%	CP091611.1
Salmonella enterica strain 1810 chromosome, complete genome	1784	1784	100%	0.0	97.60%	CP091607.1
Salmonella enterica SEOhiM2008 DNA, complete genome	1784	1784	100%	0.0	97.60%	AP024352.1
Salmonella enterica SEOhiM1960 DNA, complete genome	1784	1784	100%	0.0	97.60%	AP024349.1
Salmonella enterica SEOhiM1593 DNA, complete genome	1784	1784	100%	0.0	97.60%	AP024347.1
Salmonella enterica strain FFL chromosome, complete genome	1784	1784	100%	0.0	97.60%	CP113539.1
Salmonella enterica strain YZY chromosome, complete genome	1784	1784	100%	0.0	97.60%	CP113537.1
Salmonella enterica subsp. enterica serovar Typhimurium strain FORC_015, complete genome	1784	1784	100%	0.0	97.60%	CP011365.1
Salmonella enterica subsp. enterica strain KNP01 chromosome, complete genome	1784	1784	100%	0.0	97.60%	CP113364.1
Salmonella enterica strain 2012K-1655 chromosome 1, complete sequence	1784	1784	100%	0.0	97.60%	CP111091.1
Salmonella enterica subsp. enterica serovar London isolate N18-04674 chromosome, complete genome	1784	1784	100%	0.0	97.60%	CP082929.1
Salmonella sp. 3C chromosome, complete genome	1784	1784	100%	0.0	97.60%	CP104858.1
Salmonella enterica strain SalSpp_sample_08_No.4 chromosome, complete genome	1784	1784	100%	0.0	97.60%	CP104482.1
Salmonella enterica subsp. enterica serovar Paratyphi A strain CMCC 50973, complete genome	1784	1784	100%	0.0	97.60%	CP009049.1
Salmonella enterica subsp. enterica serovar Paratyphi A strain CMCC 50503, complete genome	1784	1784	100%	0.0	97.60%	CP009559.1
Salmonella enterica subsp. enterica serovar Kentucky strain Sal-FJ2064 chromosome, complete genome	1784	1784	100%	0.0	97.60%	CP104049.1

Description	Max Score	Total Score	Query Cover	E value	Per. Ident	Accession
Salmonella enterica subsp. enterica serovar Indiana strain XZ14C1328 chromosome, complete genome	1784	1784	100%	0.0	97.60%	CP102827.1
Salmonella enterica subsp. enterica serovar Kentucky isolate AH19MCS11 chromosome, complete genome	1784	1784	100%	0.0	97.60%	CP102756.1
Salmonella enterica subsp. enterica serovar Kentucky strain AH19MCS8 chromosome, complete genome	1784	1784	100%	0.0	97.60%	CP102739.1
Salmonella enterica subsp. enterica serovar Kentucky isolate AH19MCS1 chromosome, complete genome	1784	1784	100%	0.0	97.60%	CP102719.1
Salmonella enterica strain s15D023 chromosome, complete genome	1784	1784	100%	0.0	97.60%	CP101340.1
Salmonella enterica strain s12177 chromosome, complete genome	1784	1784	100%	0.0	97.60%	CP101348.1
Salmonella enterica strain s11011 chromosome, complete genome	1784	1784	100%	0.0	97.60%	CP101352.1
Salmonella enterica strain IndS104 chromosome, complete genome	1784	1784	100%	0.0	97.60%	CP101355.1
Salmonella enterica strain IndS102 chromosome, complete genome	1784	1784	100%	0.0	97.60%	CP101359.1
Salmonella enterica subsp. enterica serovar Kentucky strain BCID6 chromosome, complete genome	1784	1784	100%	0.0	97.60%	CP101648.1
Salmonella enterica subsp. enterica serovar Kentucky strain KCID6 chromosome, complete genome	1784	1784	100%	0.0	97.60%	CP101647.1
Salmonella enterica subsp. enterica serovar Mbandaka strain R17.4855 chromosome, complete genome	1784	1784	100%	0.0	97.60%	CP100722.1
Salmonella enterica subsp. enterica serovar London strain R18.1595 chromosome, complete genome	1784	1784	100%	0.0	97.60%	CP100693.1
Salmonella enterica subsp. enterica serovar Mbandaka strain R17.0904 chromosome, complete genome	1784	1784	100%	0.0	97.60%	CP100670.1
Salmonella enterica subsp. enterica serovar Goldcoast strain R18.0450 chromosome, complete genome	1784	1784	100%	0.0	97.60%	CP100682.1
Salmonella enterica subsp. enterica serovar Goldcoast strain R18.1297 chromosome, complete genome	1784	1784	100%	0.0	97.60%	CP100685.1
Salmonella enterica subsp. enterica serovar Indiana strain YZ20MCS14 chromosome, complete genome	1784	1784	100%	0.0	97.60%	CP098831.1
Salmonella enterica subsp. enterica serovar Indiana strain YZ20MCS6 chromosome, complete genome	1784	1784	100%	0.0	97.60%	CP098829.1
Salmonella enterica subsp. enterica serovar Indiana strain 22 chromosome, complete genome	1784	1784	100%	0.0	97.60%	CP096118.1
Salmonella enterica subsp. enterica serovar Kentucky strain N12-0259 chromosome, complete genome	1784	1784	100%	0.0	97.60%	CP092012.1
Salmonella enterica subsp. enterica serovar Kentucky strain N12-1542 chromosome, complete genome	1784	1784	100%	0.0	97.60%	CP092006.1
Salmonella enterica subsp. enterica serovar Kentucky strain N14-1660 chromosome, complete genome	1784	1784	100%	0.0	97.60%	CP092004.1
Salmonella enterica subsp. enterica serovar Kentucky strain N16-1393 chromosome, complete genome	1784	1784	100%	0.0	97.60%	CP091999.1

Description	Max Score	Total Score	Query Cover	E value	Per. Ident	Accession
Salmonella enterica subsp. enterica serovar Kentucky strain N18-2092 chromosome, complete genome	1784	1784	100%	0.0	97.60%	CP091998.1
Salmonella enterica subsp. enterica serovar Kentucky strain N20-2289 chromosome, complete genome	1784	1784	100%	0.0	97.60%	CP091997.1
Salmonella enterica subsp. enterica serovar Indiana strain LY67 chromosome, complete genome	1784	1784	100%	0.0	97.60%	CP077424.1
Salmonella enterica strain 453 chromosome, complete genome	1784	1784	100%	0.0	97.60%	CP060855.1
Salmonella enterica strain 467 chromosome, complete genome	1784	1784	100%	0.0	97.60%	CP060852.1
Salmonella enterica strain 724 chromosome, complete genome	1784	1784	100%	0.0	97.60%	CP060849.1
Salmonella enterica strain 733 chromosome, complete genome	1784	1784	100%	0.0	97.60%	CP060846.1
Salmonella enterica strain 770 chromosome, complete genome	1784	1784	100%	0.0	97.60%	CP060844.1
Salmonella enterica strain 805 chromosome, complete genome	1784	1784	100%	0.0	97.60%	CP060840.1
Salmonella enterica strain 852 chromosome, complete genome	1784	1784	100%	0.0	97.60%	CP060837.1
Salmonella enterica strain 1505 chromosome, complete genome	1784	1784	100%	0.0	97.60%	CP060834.1
Salmonella enterica subsp. enterica serovar Indiana strain 15 chromosome, complete genome	1784	1784	100%	0.0	97.60%	CP092258.1
Salmonella enterica subsp. enterica serovar Goldcoast strain 5ASAL05 chromosome, complete genome	1784	1784	100%	0.0	97.60%	CP090137.1
Salmonella enterica subsp. enterica serovar Goldcoast strain 5ASAL09 chromosome, complete genome	1784	1784	100%	0.0	97.60%	CP090141.1
Salmonella enterica subsp. enterica serovar Goldcoast strain 5ASAL07 chromosome, complete genome	1784	1784	100%	0.0	97.60%	CP090133.1
Salmonella enterica subsp. enterica serovar Kentucky strain ZTA19/00794 chromosome	1784	1784	100%	0.0	97.60%	CP089796.1
Salmonella enterica subsp. enterica serovar Kentucky strain ZTA19/00847 chromosome, complete genome	1784	1784	100%	0.0	97.60%	CP089788.1
Salmonella enterica subsp. enterica serovar Kentucky strain ZTA19/00814 chromosome, complete genome	1784	1784	100%	0.0	97.60%	CP089794.1
Salmonella enterica subsp. enterica serovar Kentucky strain ZTA19/00813 chromosome, complete genome	1784	1784	100%	0.0	97.60%	CP089795.1
Salmonella enterica subsp. enterica serovar Kentucky strain ZTA19/00816 chromosome, complete genome	1784	1784	100%	0.0	97.60%	CP089792.1
Salmonella enterica subsp. enterica serovar Kentucky strain ZTA19/00820 chromosome, complete genome	1784	1784	100%	0.0	97.60%	CP089791.1
Salmonella enterica subsp. enterica serovar Kentucky strain ZTA19/00830 chromosome, complete genome	1784	1784	100%	0.0	97.60%	CP089790.1
Salmonella enterica subsp. enterica serovar Kentucky strain ZTA19/00785 chromosome, complete genome	1784	1784	100%	0.0	97.60%	CP089799.1
Salmonella enterica subsp. enterica serovar Kentucky strain ZTA19/00789 chromosome, complete genome	1784	1784	100%	0.0	97.60%	CP089798.1

Description	Max Score	Total Score	Query Cover	E value	Per. Ident	Accession
Salmonella enterica subsp. enterica serovar Kentucky strain ZTA19/00831 chromosome, complete genome	1784	1784	100%	0.0	97.60%	CP089789.1
Salmonella enterica subsp. enterica serovar Kentucky strain ZTA19/00790 chromosome, complete genome	1784	1784	100%	0.0	97.60%	CP089797.1
Salmonella enterica subsp. enterica serovar Indiana strain YZ21MCS4 chromosome, complete genome	1784	1784	100%	0.0	97.60%	CP089313.1
Salmonella enterica subsp. enterica serovar Dublin genome assembly SC50_1, chromosome : I	1784	1784	100%	0.0	97.60%	LK931502.1
Salmonella enterica subsp. enterica serovar London strain CVM N17S347 chromosome, complete genome	1784	1784	100%	0.0	97.60%	CP082711.1
Salmonella sp. A29-2 chromosome, complete genome	1784	1784	100%	0.0	97.60%	CP083731.1
Salmonella enterica strain S90 chromosome, complete genome	1784	1784	100%	0.0	97.60%	CP077670.1
Salmonella enterica subsp. enterica serovar Kentucky strain CVM N18S0935 chromosome, complete genome	1784	1784	100%	0.0	97.60%	CP082582.1
Salmonella enterica subsp. enterica serovar London strain CVM N17S367 chromosome, complete genome	1784	1784	100%	0.0	97.60%	CP082709.1
Salmonella enterica subsp. enterica serovar Dublin strain FSIS11705856 chromosome, complete genome	1784	1784	100%	0.0	97.60%	CP082403.1
Salmonella enterica subsp. enterica serovar Ohio strain FSIS11705518 chromosome, complete genome	1784	1784	100%	0.0	97.60%	CP082406.1
Salmonella enterica subsp. enterica serovar Minnesota strain SA18578 chromosome, complete genome	1784	1784	100%	0.0	97.60%	CP080513.1
Salmonella enterica strain CFSAN057232 chromosome, complete genome	1784	1784	100%	0.0	97.60%	CP076096.1
Salmonella enterica subsp. enterica strain CFSAN058545 chromosome, complete genome	1784	1784	100%	0.0	97.60%	CP077706.1
Salmonella enterica subsp. enterica strain CFSAN058591 chromosome, complete genome	1784	1784	100%	0.0	97.60%	CP077699.1
Salmonella enterica subsp. enterica serovar Kentucky strain YZ20MCS16 chromosome, complete genome	1784	1784	100%	0.0	97.60%	CP077680.1
Salmonella enterica subsp. enterica serovar Paratyphi A strain 45157 chromosome, complete genome	1784	1784	100%	0.0	97.60%	CP076727.1
Salmonella enterica strain CFSAN044888 chromosome, complete genome	1784	1784	100%	0.0	97.60%	CP075135.1
Salmonella enterica strain CFSAN030601 chromosome, complete genome	1784	1784	100%	0.0	97.60%	CP075143.1
Salmonella enterica subsp. enterica serovar Rubislaw strain CFSAN044921 chromosome, complete genome	1784	1784	100%	0.0	97.60%	CP075129.1
Salmonella enterica strain CFSAN044945 chromosome, complete genome	1784	1784	100%	0.0	97.60%	CP075125.1
Salmonella enterica subsp. enterica serovar Eastbourne strain CFSAN059883 chromosome, complete genome	1784	1784	100%	0.0	97.60%	CP075115.1
Salmonella enterica subsp. enterica serovar Dublin strain CFSAN059898 chromosome, complete genome	1784	1784	100%	0.0	97.60%	CP075113.1
Salmonella enterica strain CFSAN073549 chromosome, complete genome	1784	1784	100%	0.0	97.60%	CP075104.1

Description	Max Score	Total Score	Query Cover	E value	Per. Ident	Accession
Salmonella enterica subsp. enterica serovar Mbandaka strain FDA0945290-S001-001 isolate CFSAN082805 chromosome, complete genome	1784	1784	100%	0.0	97.60%	CP075100.1
Salmonella enterica subsp. enterica serovar Dublin strain CFSAN022635 chromosome, complete genome	1784	1784	100%	0.0	97.60%	CP075021.1
Salmonella enterica subsp. salamae serovar 6,7:m,t:- strain CFSAN028548 chromosome, complete genome	1784	1784	100%	0.0	97.60%	CP074649.1
Salmonella enterica strain CFSAN029856 chromosome, complete genome	1784	1784	100%	0.0	97.60%	CP074653.1
Salmonella enterica strain CFSAN029865 chromosome, complete genome	1784	1784	100%	0.0	97.60%	CP074640.1
Salmonella enterica strain CFSAN029872 chromosome, complete genome	1784	1784	100%	0.0	97.60%	CP074633.1
Salmonella enterica subsp. enterica serovar Oranienburg strain CFSAN012497 chromosome, complete genome	1784	1784	100%	0.0	97.60%	CP075041.1
Salmonella enterica strain CFSAN029939 chromosome, complete genome	1784	1784	100%	0.0	97.60%	CP074623.1
Salmonella enterica subsp. enterica serovar Indiana strain SGSC 2482 chromosome, complete genome	1784	1784	100%	0.0	97.60%	CP074612.1
Salmonella enterica subsp. enterica serovar Paratyphi B str. CFSAN000540 strain SGSC 2238 isolate SARA58 chromosome, complete genome	1784	1784	100%	0.0	97.60%	CP074610.1
Salmonella enterica subsp. salamae strain CFSAN001011 chromosome, complete genome	1784	1784	100%	0.0	97.60%	CP074599.1
Salmonella enterica subsp. enterica serovar Rubislaw strain CFSAN006171 chromosome, complete genome	1784	1784	100%	0.0	97.60%	CP074294.1
Salmonella enterica subsp. enterica serovar Minnesota strain CFSAN006156 chromosome, complete genome	1784	1784	100%	0.0	97.60%	CP074298.1
Salmonella enterica subsp. enterica serovar Rubislaw strain CFSAN024580 chromosome, complete genome	1784	1784	100%	0.0	97.60%	CP074269.1
Salmonella enterica subsp. enterica serovar Livingstone strain CFSAN024717 chromosome, complete genome	1784	1784	100%	0.0	97.60%	CP074262.1
Salmonella enterica subsp. enterica serovar Rubislaw strain CFSAN027379 chromosome, complete genome	1784	1784	100%	0.0	97.60%	CP074251.1
Salmonella enterica subsp. enterica serovar Minnesota strain CFSAN024581 chromosome, complete genome	1784	1784	100%	0.0	97.60%	CP074268.1
Salmonella enterica subsp. enterica serovar Rubislaw strain CFSAN024587 chromosome, complete genome	1784	1784	100%	0.0	97.60%	CP074267.1
Salmonella enterica subsp. enterica serovar Dublin str. CFSAN000516 strain SGSC 2469 isolate SARB12 chromosome, complete genome	1784	1784	100%	0.0	97.60%	CP074229.1
Salmonella enterica subsp. enterica serovar London str. CFSAN001081 chromosome, complete genome	1784	1784	100%	0.0	97.60%	CP074204.1
Salmonella enterica subsp. enterica serovar Eastbourne str. CFSAN001084 chromosome, complete genome	1784	1784	100%	0.0	97.60%	CP074201.1
Salmonella enterica subsp. enterica strain Se29 chromosome, complete genome	1784	1784	100%	0.0	97.60%	CP070222.1

Description	Max Score	Total Score	Query Cover	E value	Per. Ident	Accession
Salmonella enterica subsp. enterica serovar London strain CVM 35153 chromosome, complete genome	1784	1784	100%	0.0	97.60%	CP051313.1
Salmonella enterica strain GX1006 chromosome, complete genome	1784	1784	100%	0.0	97.60%	CP060585.1
Salmonella enterica subsp. enterica serovar Indiana strain S530 chromosome, complete genome	1784	1784	100%	0.0	97.60%	CP061118.1
Salmonella sp. SJTUF14076 chromosome, complete genome	1784	1784	100%	0.0	97.60%	CP064674.1
Salmonella sp. SJTUF14146 chromosome, complete genome	1784	1784	100%	0.0	97.60%	CP064672.1
Salmonella sp. SJTUF14152 chromosome, complete genome	1784	1784	100%	0.0	97.60%	CP064671.1
Salmonella sp. SJTUF14154 chromosome, complete genome	1784	1784	100%	0.0	97.60%	CP064666.1
Salmonella sp. SJTUF14178 chromosome, complete genome	1784	1784	100%	0.0	97.60%	CP064661.1
Salmonella sp. SJTUF14170 chromosome, complete genome	1784	1784	100%	0.0	97.60%	CP064663.1
Salmonella enterica subsp. enterica serovar Dublin strain 18SC06VL09-S2 chromosome, complete genome	1784	1784	100%	0.0	97.60%	CP063756.1
Salmonella enterica subsp. enterica serovar Dublin strain 18SC12VL05-S4 chromosome, complete genome	1784	1784	100%	0.0	97.60%	CP063754.1
Salmonella enterica subsp. enterica serovar Goldcoast strain R18.1074 chromosome, complete genome	1784	1784	100%	0.0	97.60%	CP062225.1
Salmonella enterica subsp. enterica serovar Goldcoast strain R18.1656 chromosome, complete genome	1784	1784	100%	0.0	97.60%	CP062223.1
Salmonella enterica subsp. enterica serovar London strain HA1-SP5,complete sequence	1784	1784	100%	0.0	97.60%	CP060134.1
Salmonella enterica subsp. enterica serovar London strain HA3-IN1, complete sequence	1784	1784	100%	0.0	97.60%	CP060132.1
Salmonella enterica strain K_SA184 chromosome, complete genome	1784	1784	100%	0.0	97.60%	CP061159.1
Salmonella enterica strain SLR1_7697 chromosome, complete genome	1784	1784	100%	0.0	97.60%	CP060508.1
Salmonella enterica subsp. enterica serovar Indiana strain 222 chromosome, complete genome	1784	1784	100%	0.0	97.60%	CP031189.1
Salmonella enterica subsp. enterica strain CFSA1007 chromosome, complete genome	1784	1784	100%	0.0	97.60%	CP033387.2
Salmonella enterica subsp. enterica strain CFSA300 chromosome, complete genome	1784	1784	100%	0.0	97.60%	CP033384.2
Salmonella enterica subsp. enterica serovar Kentucky strain VNSEC013 chromosome, complete genome	1784	1784	100%	0.0	97.60%	CP039439.1
Salmonella enterica subsp. enterica serovar Dublin str. CT_02021853, complete genome	1784	1784	100%	0.0	97.60%	CP001144.1
Salmonella enterica subsp. enterica serovar Paratyphi A str. AKU_12601 complete genome, strain AKU_12601	1784	1784	100%	0.0	97.60%	FM200053.1
Salmonella enterica subsp. enterica serovar Paratyphi A str. ATCC 9150, complete genome	1784	1784	100%	0.0	97.60%	CP000026.1

Description	Max Score	Total Score	Query Cover	E value	Per. Ident	Accession
Salmonella enterica subsp. salamae strain NCTC8273 genome assembly, chromosome: 1	1783	1783	100%	0.0	97.60%	LR134154.1
Salmonella enterica subsp. enterica serovar Infantis strain CVM N17S1509 chromosome, complete genome	1779	1779	100%	0.0	97.50%	CP052817.1
Salmonella enterica subsp. enterica serovar Infantis strain CVM N16S024 chromosome, complete genome	1779	1779	100%	0.0	97.50%	CP052839.1
Salmonella enterica subsp. enterica serovar Infantis strain CVM N16S097 chromosome, complete genome	1779	1779	100%	0.0	97.50%	CP052837.1
Salmonella enterica subsp. enterica serovar Infantis strain CVM N16S103 chromosome, complete genome	1779	1779	100%	0.0	97.50%	CP052835.1
Salmonella enterica subsp. enterica serovar Infantis strain CVM N17S041 chromosome, complete genome	1779	1779	100%	0.0	97.50%	CP052833.1
Salmonella enterica subsp. enterica serovar Infantis strain CVM N17S1040 chromosome, complete genome	1779	1779	100%	0.0	97.50%	CP052831.1
Salmonella enterica subsp. enterica serovar Infantis strain CVM N17S1105 chromosome, complete genome	1779	1779	100%	0.0	97.50%	CP052829.1
Salmonella enterica subsp. enterica serovar Infantis strain CVM N17S1126 chromosome, complete genome	1779	1779	100%	0.0	97.50%	CP052827.1
Salmonella enterica subsp. enterica serovar Infantis strain CVM N17S1245 chromosome, complete genome	1779	1779	100%	0.0	97.50%	CP052825.1
Salmonella enterica subsp. enterica serovar Infantis strain CVM N17S1265 chromosome, complete genome	1779	1779	100%	0.0	97.50%	CP052823.1
Salmonella enterica subsp. enterica serovar Infantis strain CVM N17S1349 chromosome, complete genome	1779	1779	100%	0.0	97.50%	CP052821.1
Salmonella enterica subsp. enterica serovar Infantis strain CVM N17S1442 chromosome, complete genome	1779	1779	100%	0.0	97.50%	CP052819.1
Salmonella enterica subsp. enterica serovar Infantis strain CVM N17S1598 chromosome, complete genome	1779	1779	100%	0.0	97.50%	CP052815.1
Salmonella enterica subsp. enterica serovar Infantis strain CVM N17S349 chromosome, complete genome	1779	1779	100%	0.0	97.50%	CP052813.1
Salmonella enterica subsp. enterica serovar Infantis strain CVM N17S376 chromosome, complete genome	1779	1779	100%	0.0	97.50%	CP052811.1
Salmonella enterica subsp. enterica serovar Infantis strain CVM N17S535 chromosome, complete genome	1779	1779	100%	0.0	97.50%	CP052809.1
Salmonella enterica subsp. enterica serovar Infantis strain CVM N17S637 chromosome, complete genome	1779	1779	100%	0.0	97.50%	CP052807.1
Salmonella enterica subsp. enterica serovar Infantis strain CVM N17S816 chromosome, complete genome	1779	1779	100%	0.0	97.50%	CP052805.1
Salmonella enterica subsp. enterica serovar Infantis strain CVM N17S990 chromosome, complete genome	1779	1779	100%	0.0	97.50%	CP052798.1
Salmonella enterica subsp. enterica serovar Infantis strain CVM N18S2039 chromosome, complete genome	1779	1779	100%	0.0	97.50%	CP052796.1
Salmonella enterica subsp. enterica serovar Infantis strain CVM N19S0125 chromosome, complete genome	1779	1779	100%	0.0	97.50%	CP052794.1

Description	Max Score	Total Score	Query Cover	E value	Per. Ident	Accession
Salmonella enterica subsp. enterica serovar Infantis strain CVM N17S973 chromosome, complete genome	1779	1779	100%	0.0	97.50%	CP052803.1
Salmonella enterica subsp. enterica serovar Infantis strain CVM N17S976 chromosome, complete genome	1779	1779	100%	0.0	97.50%	CP052801.1
Salmonella enterica subsp. enterica serovar Infantis strain CVM N19S0388 chromosome, complete genome	1779	1779	100%	0.0	97.50%	CP052792.1
Salmonella enterica subsp. enterica serovar Infantis strain CVM N19S0552 chromosome, complete genome	1779	1779	100%	0.0	97.50%	CP052789.1
Salmonella enterica subsp. enterica serovar Infantis strain CVM N19S0611 chromosome, complete genome	1779	1779	100%	0.0	97.50%	CP052787.1
Salmonella enterica subsp. enterica serovar Infantis strain CVM N19S0641 chromosome, complete genome	1779	1779	100%	0.0	97.50%	CP052785.1
Salmonella enterica subsp. enterica serovar Infantis strain CVM N19S0679 chromosome, complete genome	1779	1779	100%	0.0	97.50%	CP052782.1
Salmonella enterica strain CVM N19S0949 chromosome, complete genome	1779	1779	100%	0.0	97.50%	CP052780.1
Salmonella enterica strain 19TN07GT06K-S chromosome, complete genome	1779	1779	100%	0.0	97.50%	CP052778.1
Salmonella enterica subsp. enterica serovar Infantis strain CVM N18S1246 chromosome, complete genome	1779	1779	100%	0.0	97.50%	CP052777.1
Salmonella enterica subsp. enterica serovar Infantis strain CVM N18S2085 chromosome, complete genome	1779	1779	100%	0.0	97.50%	CP052776.1
Salmonella enterica subsp. enterica serovar Infantis strain CVM N19S0145 chromosome, complete genome	1779	1779	100%	0.0	97.50%	CP052775.1
Salmonella enterica subsp. enterica serovar Infantis strain CVM N17S1234 chromosome, complete genome	1779	1779	100%	0.0	97.50%	CP051675.1
Salmonella enterica subsp. enterica serovar Kedougou strain Sal162 chromosome	1779	1779	100%	0.0	97.50%	CP053214.1
Salmonella enterica subsp. enterica serovar Schwarzengrund strain WAPHL_SAL-A00527 chromosome, complete genome	1779	1779	100%	0.0	97.50%	CP048297.1
Salmonella enterica subsp. enterica serovar Schwarzengrund strain WAPHL-SAL-A00375 chromosome, complete genome	1779	1779	100%	0.0	97.50%	CP048301.1
Salmonella enterica strain FDAARGOS_686 chromosome	1779	1779	100%	0.0	97.50%	CP050968.1
Salmonella enterica subsp. enterica serovar Infantis strain SPE55 chromosome	1779	1779	100%	0.0	97.50%	CP040065.1
Salmonella enterica subsp. enterica serovar Infantis strain SPE100 chromosome	1779	1779	100%	0.0	97.50%	CP040063.1
Salmonella enterica subsp. enterica serovar Infantis strain SPE102 chromosome	1779	1779	100%	0.0	97.50%	CP040164.1
Salmonella enterica subsp. enterica serovar Infantis strain SBO17 chromosome	1779	1779	100%	0.0	97.50%	CP040163.1
Salmonella enterica subsp. enterica serovar Infantis strain SBO53 chromosome	1779	1779	100%	0.0	97.50%	CP040061.1
Salmonella enterica strain 2010K-2370 chromosome, complete genome	1779	1779	100%	0.0	97.50%	CP035639.1
Salmonella enterica subsp. enterica serovar Infantis strain 119944 chromosome, complete genome	1779	1779	100%	0.0	97.50%	CP047881.1
Salmonella enterica subsp. enterica serovar Bareilly str. CFSAN000189 chromosome, complete genome	1779	1779	100%	0.0	97.50%	CP034177.1

Description	Max Score	Total Score	Query Cover	E value	Per. Ident	Accession
Salmonella enterica subsp. enterica serovar Muenchen strain LG26 chromosome, complete genome	1779	1779	100%	0.0	97.50%	CP045063.1
Salmonella enterica subsp. enterica serovar Muenchen strain LG25 chromosome, complete genome	1779	1779	100%	0.0	97.50%	CP045059.1
Salmonella enterica subsp. enterica serovar Muenchen strain LG24 chromosome, complete genome	1779	1779	100%	0.0	97.50%	CP045056.1
Salmonella enterica subsp. enterica serovar Bareilly str. CFSAN000661 chromosome, complete genome	1779	1779	100%	0.0	97.50%	CP045759.1
Salmonella enterica subsp. enterica serovar Bareilly str. CFSAN000189 chromosome, complete genome	1779	1779	100%	0.0	97.50%	CP045753.1
Salmonella enterica subsp. enterica serovar Bareilly str. CFSAN000669 chromosome, complete genome	1779	1779	100%	0.0	97.50%	CP045757.1
Salmonella enterica subsp. enterica serovar Bareilly str. CFSAN000752 chromosome, complete genome	1779	1779	100%	0.0	97.50%	CP045754.1
Salmonella enterica subsp. enterica serovar Schwarzengrund strain 12888 chromosome, complete genome	1779	1779	100%	0.0	97.50%	CP045447.1
Salmonella enterica subsp. enterica serovar Schwarzengrund strain 9355 chromosome, complete genome	1779	1779	100%	0.0	97.50%	CP045444.1
Salmonella enterica subsp. enterica serovar Bredeney strain SA20114778WT chromosome, complete genome	1779	1779	100%	0.0	97.50%	CP043222.1
Salmonella enterica subsp. enterica serovar Infantis strain FARPER-219 chromosome, complete genome	1779	1779	100%	0.0	97.50%	CP038507.1
Salmonella enterica subsp. enterica serovar Senftenberg SL180013 DNA, complete genome	1779	1779	100%	0.0	97.50%	AP019692.1
Salmonella enterica subsp. enterica serovar Infantis strain TR01 chromosome	1779	1779	100%	0.0	97.50%	CP040600.1
Salmonella enterica subsp. enterica serovar Infantis strain TR01 Mutant chromosome	1779	1779	100%	0.0	97.50%	CP040601.1
Salmonella enterica subsp. enterica serovar Bareilly str. CFSAN000191 chromosome, complete genome	1779	1779	100%	0.0	97.50%	CP032622.2
Salmonella enterica subsp. enterica serovar Bareilly str. CFSAN000211 chromosome, complete genome	1779	1779	100%	0.0	97.50%	CP039500.1
Salmonella enterica subsp. enterica serovar Bareilly str. CFSAN000212 chromosome, complete genome	1779	1779	100%	0.0	97.50%	CP039499.1
Salmonella enterica subsp. enterica serovar Bareilly str. CFSAN000228 chromosome, complete genome	1779	1779	100%	0.0	97.50%	CP039498.1
Salmonella enterica subsp. enterica serovar Bareilly str. CFSAN000661 chromosome, complete genome	1779	1779	100%	0.0	97.50%	CP039496.1
Salmonella enterica subsp. enterica serovar Bareilly str. CFSAN000189 chromosome, complete genome	1779	1779	100%	0.0	97.50%	CP039502.1
Salmonella enterica subsp. enterica serovar Bareilly str. CFSAN000669 chromosome, complete genome	1779	1779	100%	0.0	97.50%	CP039493.1
Salmonella enterica subsp. enterica serovar Bareilly str. CFSAN000968 chromosome, complete genome	1779	1779	100%	0.0	97.50%	CP039473.1

Description	Max Score	Total Score	Query Cover	E value	Per. Ident	Accession
Salmonella enterica subsp. enterica serovar Bareilly str. CFSAN000958 chromosome, complete genome	1779	1779	100%	0.0	97.50%	CP039480.1
Salmonella enterica subsp. enterica serovar Bareilly str. CFSAN000700 chromosome, complete genome	1779	1779	100%	0.0	97.50%	CP039491.1
Salmonella enterica subsp. enterica serovar Bareilly str. CFSAN000753 chromosome, complete genome	1779	1779	100%	0.0	97.50%	CP039487.1
Salmonella enterica subsp. enterica serovar Bareilly str. CFSAN000752 chromosome, complete genome	1779	1779	100%	0.0	97.50%	CP039489.1
Salmonella enterica subsp. enterica serovar Bareilly str. CFSAN000963 chromosome, complete genome	1779	1779	100%	0.0	97.50%	CP039476.1
Salmonella enterica subsp. enterica serovar Bareilly str. CFSAN000970 chromosome, complete genome	1779	1779	100%	0.0	97.50%	CP039471.1
Salmonella enterica subsp. enterica serovar Bareilly str. CFSAN000952 chromosome, complete genome	1779	1779	100%	0.0	97.50%	CP039484.1
Salmonella enterica subsp. enterica serovar Bareilly str. CFSAN000961 chromosome, complete genome	1779	1779	100%	0.0	97.50%	CP039477.1
Salmonella enterica subsp. enterica serovar Bareilly str. CFSAN000954 chromosome, complete genome	1779	1779	100%	0.0	97.50%	CP039482.1
Salmonella enterica subsp. enterica serovar Bareilly str. CFSAN000960 chromosome, complete genome	1779	1779	100%	0.0	97.50%	CP039478.1
Salmonella enterica subsp. enterica serovar Bareilly str. CFSAN001112 chromosome, complete genome	1779	1779	100%	0.0	97.50%	CP039469.1
Salmonella enterica subsp. enterica serovar Bareilly str. CFSAN000951 chromosome, complete genome	1779	1779	100%	0.0	97.50%	CP039486.1
Salmonella enterica subsp. enterica serovar Bareilly str. CFSAN001115 chromosome, complete genome	1779	1779	100%	0.0	97.50%	CP039467.1
Salmonella enterica subsp. enterica serovar Bareilly str. CFSAN001118 chromosome, complete genome	1779	1779	100%	0.0	97.50%	CP039465.1
Salmonella enterica subsp. enterica serovar Bareilly str. CFSAN001140 chromosome, complete genome	1779	1779	100%	0.0	97.50%	CP039463.1
Salmonella enterica subsp. enterica serovar Senftenberg strain CFIAFB20170119 chromosome, complete genome	1779	1779	100%	0.0	97.50%	CP039279.1
Salmonella enterica subsp. enterica serovar Senftenberg strain GTA-FD-2016-MI-02533-3 chromosome, complete genome	1779	1779	100%	0.0	97.50%	CP038608.1
Salmonella enterica subsp. enterica serovar Senftenberg strain GTA-FD-2016-MI-02533-2 chromosome, complete genome	1779	1779	100%	0.0	97.50%	CP038604.1
Salmonella enterica subsp. enterica serovar Infantis strain L41 chromosome	1779	1779	100%	0.0	97.50%	CP038516.1
Salmonella enterica strain FSW0104 chromosome, complete genome	1779	1779	100%	0.0	97.50%	CP037894.1
Salmonella enterica subsp. enterica strain CFSAN087304 chromosome, complete genome	1779	1779	100%	0.0	97.50%	CP037892.1
Salmonella enterica strain SA20041606 chromosome	1779	1779	100%	0.0	97.50%	CP030236.1
Salmonella enterica subsp. enterica serovar Berta strain SA20141895 chromosome, complete genome	1779	1779	100%	0.0	97.50%	CP030005.1

Description	Max Score	Total Score	Query Cover	E value	Per. Ident	Accession
Salmonella enterica subsp. enterica serovar Napoli strain LC0541/17 chromosome, complete genome	1779	1779	100%	0.0	97.50%	CP030838.1
Salmonella enterica subsp. enterica serovar Bredeney strain NCTC6026 genome assembly, chromosome: 1	1779	1779	100%	0.0	97.50%	LS483481.1
Salmonella enterica subsp. enterica strain 15-SA01028 chromosome, complete genome	1779	1779	100%	0.0	97.50%	CP026660.1
Salmonella enterica subsp. enterica serovar Infantis strain CFSAN003307 chromosome, complete genome	1779	1779	100%	0.0	97.50%	CP019202.1
Salmonella enterica subsp. enterica serovar Give strain CFSAN024229 chromosome, complete genome	1779	1779	100%	0.0	97.50%	CP019174.1
Salmonella enterica subsp. enterica strain RM11060 chromosome, complete genome	1779	1779	100%	0.0	97.50%	CP022658.1
Salmonella enterica subsp. enterica serovar Kentucky str. SA20030505 chromosome, complete genome	1779	1779	100%	0.0	97.50%	CP022500.1
Salmonella enterica subsp. salamae serovar 57:z29:z42 strain ST114, complete genome	1779	1779	100%	0.0	97.50%	CP022467.1
Salmonella enterica subsp. enterica serovar Quebec str. S-1267, complete genome	1779	1779	100%	0.0	97.50%	CP022019.1
Salmonella enterica subsp. enterica serovar Onderstepoort str. SA20060086, complete genome	1779	1779	100%	0.0	97.50%	CP022034.1
Salmonella enterica subsp. enterica serovar Krefeld str. SA20030536, complete genome	1779	1779	100%	0.0	97.50%	CP019413.1
Salmonella enterica subsp. enterica serovar Senftenberg strain 775W, complete genome	1779	1779	100%	0.0	97.50%	CP016837.1
Salmonella enterica subsp. enterica serovar Infantis strain N55391, complete genome	1779	1779	100%	0.0	97.50%	CP016410.1
Salmonella enterica subsp. enterica serovar Infantis strain FSIS1502916, complete genome	1779	1779	100%	0.0	97.50%	CP016408.1
Salmonella enterica subsp. enterica serovar Infantis strain FSIS1502169, complete genome	1779	1779	100%	0.0	97.50%	CP016406.1
Salmonella enterica subsp. enterica serovar Infantis strain CVM44454, complete genome	1779	1779	100%	0.0	97.50%	CP016412.1
Salmonella enterica subsp. enterica serovar Infantis strain 152369 chromosome, complete genome	1779	1779	100%	0.0	97.50%	CP113983.1
Salmonella enterica subsp. enterica serovar Infantis strain 160771 chromosome, complete genome	1779	1779	100%	0.0	97.50%	CP113986.1
Salmonella enterica subsp. enterica serovar Infantis strain 557658 chromosome, complete genome	1779	1779	100%	0.0	97.50%	CP113987.1
Salmonella enterica subsp. enterica serovar Infantis strain 1038175 chromosome, complete genome	1779	1779	100%	0.0	97.50%	CP113990.1
Salmonella enterica subsp. enterica serovar Infantis strain 1042326 chromosome, complete genome	1779	1779	100%	0.0	97.50%	CP113992.1
Salmonella enterica subsp. enterica serovar Infantis strain 1044958 chromosome, complete genome	1779	1779	100%	0.0	97.50%	CP113994.1

Description	Max Score	Total Score	Query Cover	E value	Per. Ident	Accession
Salmonella enterica subsp. enterica serovar Infantis strain 1045028 chromosome, complete genome	1779	1779	100%	0.0	97.50%	CP113996.1
Salmonella enterica subsp. enterica serovar Infantis strain 1048349 chromosome, complete genome	1779	1779	100%	0.0	97.50%	CP113999.1
Salmonella enterica subsp. enterica serovar Infantis strain 1048341 chromosome, complete genome	1779	1779	100%	0.0	97.50%	CP114000.1
Salmonella enterica subsp. enterica serovar Infantis strain 1059469 chromosome, complete genome	1779	1779	100%	0.0	97.50%	CP114002.1
Salmonella enterica subsp. enterica serovar Infantis strain 1375619 chromosome, complete genome	1779	1779	100%	0.0	97.50%	CP114005.1
Salmonella enterica strain 1902 chromosome, complete genome	1779	1779	100%	0.0	97.50%	CP091604.1
Salmonella enterica SE20-C72-2 DNA, complete genome	1779	1779	100%	0.0	97.50%	AP026948.1
Salmonella enterica strain SalSpp_sample_09_No.5 chromosome, complete genome	1779	1779	100%	0.0	97.50%	CP104479.1
Salmonella enterica strain SalSpp_sample_10_No.2 chromosome, complete genome	1779	1779	100%	0.0	97.50%	CP104488.1
Salmonella enterica subsp. enterica serovar Infantis strain MRS17_00712 chromosome, complete genome	1779	1779	100%	0.0	97.50%	CP103791.1
Salmonella enterica subsp. enterica serovar Infantis genome assembly SINFA, chromosome : 1	1779	1779	100%	0.0	97.50%	LN649235.1
Salmonella enterica subsp. enterica serovar Infantis strain 423_13 chromosome, complete genome	1779	1779	100%	0.0	97.50%	CP093373.1
Salmonella enterica subsp. enterica serovar Kentucky strain N12-0931 chromosome, complete genome	1779	1779	100%	0.0	97.50%	CP092009.1
Salmonella enterica strain MS114 chromosome, complete genome	1779	1779	100%	0.0	97.50%	CP058671.1
Salmonella enterica subsp. enterica serovar Infantis strain R21.1147 chromosome, complete genome	1779	1779	100%	0.0	97.50%	CP093400.1
Salmonella enterica subsp. enterica serovar Muenchen strain 180135033 chromosome, complete genome	1779	1779	100%	0.0	97.50%	CP088901.1
Salmonella enterica strain PNUSAS047891 chromosome, complete genome	1779	1779	100%	0.0	97.50%	CP092301.1
Salmonella enterica strain PNUSAS041145 chromosome, complete genome	1779	1779	100%	0.0	97.50%	CP092297.1
Salmonella enterica strain PNUSAS070160 chromosome, complete genome	1779	1779	100%	0.0	97.50%	CP092294.1
Salmonella enterica subsp. enterica serovar Infantis strain S19 chromosome, complete genome	1779	1779	100%	0.0	97.50%	CP092040.1
Salmonella enterica subsp. enterica strain 679 chromosome, complete genome	1779	1779	100%	0.0	97.50%	CP067082.1
Salmonella enterica strain SZL 30 chromosome, complete genome	1779	1779	100%	0.0	97.50%	CP085981.1
Salmonella enterica strain SZL 31 chromosome, complete genome	1779	1779	100%	0.0	97.50%	CP085983.1
Salmonella enterica subsp. enterica serovar Schwarzengrund strain Moltres chromosome, complete genome	1779	1779	100%	0.0	97.50%	CP085810.1

Description	Max Score	Total Score	Query Cover	E value	Per. Ident	Accession
Salmonella enterica subsp. enterica serovar Schwarzengrund strain Articuno chromosome, complete genome	1779	1779	100%	0.0	97.50%	CP085812.1
Salmonella enterica subsp. enterica serovar Schwarzengrund strain Zapdos chromosome, complete genome	1779	1779	100%	0.0	97.50%	CP085811.1
Salmonella sp. JXY0409-18 chromosome, complete genome	1779	1779	100%	0.0	97.50%	CP084216.1
Salmonella enterica subsp. enterica serovar Bredeney str. CFSAN001080, complete genome	1779	1779	100%	0.0	97.50%	CP007533.1
Salmonella enterica subsp. enterica serovar Infantis strain CVM N18S2198 chromosome, complete genome	1779	1779	100%	0.0	97.50%	CP082521.1
Salmonella enterica subsp. enterica serovar Kentucky strain CVM N18S2145 chromosome, complete genome	1779	1779	100%	0.0	97.50%	CP082535.1
Salmonella enterica subsp. enterica serovar Infantis strain CVM N18S2042 chromosome, complete genome	1779	1779	100%	0.0	97.50%	CP082536.1
Salmonella enterica subsp. enterica serovar Schwarzengrund strain CVM N18S1602 chromosome, complete genome	1779	1779	100%	0.0	97.50%	CP082554.1
Salmonella enterica subsp. enterica serovar Kentucky strain CVM N18S1563 chromosome, complete genome	1779	1779	100%	0.0	97.50%	CP082565.1
Salmonella enterica subsp. enterica serovar Kentucky strain CVM N18S1260 chromosome, complete genome	1779	1779	100%	0.0	97.50%	CP082570.1
Salmonella enterica subsp. enterica serovar Bredeney strain CVM N18S0406 chromosome, complete genome	1779	1779	100%	0.0	97.50%	CP082691.1
Salmonella enterica subsp. enterica serovar Kentucky strain CVM N18S0236 chromosome, complete genome	1779	1779	100%	0.0	97.50%	CP082695.1
Salmonella enterica subsp. enterica serovar Kentucky strain CVM N18S0133 chromosome, complete genome	1779	1779	100%	0.0	97.50%	CP082698.1
Salmonella enterica subsp. enterica serovar Infantis strain CVM N17S1592 chromosome, complete genome	1779	1779	100%	0.0	97.50%	CP082617.1
Salmonella enterica subsp. enterica serovar Kentucky strain CVM N17S1545 chromosome, complete genome	1779	1779	100%	0.0	97.50%	CP082619.1
Salmonella enterica subsp. enterica serovar Berta strain CVM N17S334 chromosome, complete genome	1779	1779	100%	0.0	97.50%	CP082714.1
Salmonella enterica subsp. enterica serovar Berta strain CVM N17S266 chromosome, complete genome	1779	1779	100%	0.0	97.50%	CP082723.1
Salmonella enterica subsp. enterica serovar Kentucky strain CVM N16S104 chromosome, complete genome	1779	1779	100%	0.0	97.50%	CP082654.1
Salmonella enterica subsp. enterica serovar Infantis strain FSIS1703084 chromosome, complete genome	1779	1779	100%	0.0	97.50%	CP082372.1
Salmonella enterica subsp. enterica serovar Kentucky strain CVM N16S065 chromosome, complete genome	1779	1779	100%	0.0	97.50%	CP082747.1
Salmonella enterica subsp. enterica serovar Infantis strain FSIS1700566 chromosome, complete genome	1779	1779	100%	0.0	97.50%	CP082389.1
Salmonella enterica subsp. enterica serovar Ohio strain FSIS1606132 chromosome	1779	1779	100%	0.0	97.50%	CP082511.1

Description	Max Score	Total Score	Query Cover	E value	Per. Ident	Accession
Salmonella enterica subsp. enterica serovar Kentucky strain CVM N17S323 isolate 17PA04CB05-S2 chromosome, complete genome	1779	1779	100%	0.0	97.50%	CP082716.1
Salmonella enterica subsp. enterica serovar Kentucky strain CVM N17S914 isolate 17GA09CB08-S2 chromosome, complete genome	1779	1779	100%	0.0	97.50%	CP082602.1
Salmonella enterica subsp. enterica serovar Schwarzengrund strain CVM N17S1304 isolate 17GA11GT12-S2 chromosome, complete genome	1779	1779	100%	0.0	97.50%	CP082636.1
Salmonella enterica subsp. enterica serovar Infantis strain CVM N18S1988 isolate 18TX11CB22-S1 chromosome, complete genome	1779	1779	100%	0.0	97.50%	CP082538.1
Salmonella enterica subsp. enterica serovar Schwarzengrund strain S16 chromosome, complete genome	1779	1779	100%	0.0	97.50%	CP081858.1
Salmonella enterica subsp. salamae strain LHICA_SA2 chromosome, complete genome	1779	1779	100%	0.0	97.50%	CP079837.1
Salmonella enterica subsp. enterica strain CFSAN058598 chromosome, complete genome	1779	1779	100%	0.0	97.50%	CP077696.1
Salmonella enterica subsp. enterica strain CFSAN058605 chromosome, complete genome	1779	1779	100%	0.0	97.50%	CP077693.1
Salmonella enterica subsp. enterica strain CFSAN058620 chromosome, complete genome	1779	1779	100%	0.0	97.50%	CP077691.1
Salmonella enterica strain CFSAN060804 chromosome, complete genome	1779	1779	100%	0.0	97.50%	CP075110.1
Salmonella enterica strain CFSAN060809 chromosome, complete genome	1779	1779	100%	0.0	97.50%	CP075107.1
Salmonella enterica strain CFSAN073555 chromosome, complete genome	1779	1779	100%	0.0	97.50%	CP075101.1
Salmonella enterica subsp. enterica serovar Muenchen strain CFSAN008798 chromosome, complete genome	1779	1779	100%	0.0	97.50%	CP075048.1
Salmonella enterica subsp. enterica serovar Infantis strain CFSAN012496 chromosome, complete genome	1779	1779	100%	0.0	97.50%	CP075042.1
Salmonella enterica strain CFSAN024144 chromosome, complete genome	1779	1779	100%	0.0	97.50%	CP075017.1
Salmonella enterica strain CFSAN024230 chromosome, complete genome	1779	1779	100%	0.0	97.50%	CP075011.1
Salmonella enterica strain FDA00008277 chromosome, complete genome	1779	1779	100%	0.0	97.50%	CP074650.1
Salmonella enterica strain CFSAN029871 chromosome, complete genome	1779	1779	100%	0.0	97.50%	CP074636.1
Salmonella enterica strain CFSAN029894 chromosome, complete genome	1779	1779	100%	0.0	97.50%	CP074627.1
Salmonella enterica subsp. enterica serovar Senftenberg strain CFSAN001066 chromosome, complete genome	1779	1779	100%	0.0	97.50%	CP074591.1
Salmonella enterica subsp. salamae strain CFSAN001013 chromosome, complete genome	1779	1779	100%	0.0	97.50%	CP074598.1
Salmonella enterica subsp. enterica serovar Infantis strain CFSAN059939 chromosome, complete genome	1779	1779	100%	0.0	97.50%	CP074343.1
Salmonella enterica subsp. enterica serovar Schwarzengrund strain CFSAN008848 chromosome, complete genome	1779	1779	100%	0.0	97.50%	CP074340.1

Description	Max Score	Total Score	Query Cover	E value	Per. Ident	Accession
Salmonella enterica subsp. enterica serovar Infantis strain CFSAN059940 chromosome, complete genome	1779	1779	100%	0.0	97.50%	CP074341.1
Salmonella enterica subsp. enterica serovar Kentucky strain CFSAN011778 chromosome, complete genome	1779	1779	100%	0.0	97.50%	CP074339.1
Salmonella enterica subsp. enterica serovar Muenchen strain CFSAN024517 chromosome, complete genome	1779	1779	100%	0.0	97.50%	CP074332.1
Salmonella enterica strain CFSAN024522 chromosome, complete genome	1779	1779	100%	0.0	97.50%	CP074331.1
Salmonella enterica subsp. enterica serovar Soerenga str. 695 chromosome, complete genome	1779	1779	100%	0.0	97.50%	CP074317.1
Salmonella enterica subsp. enterica serovar Buzu strain CFSAN008300 chromosome, complete genome	1779	1779	100%	0.0	97.50%	CP074275.1
Salmonella enterica subsp. enterica serovar Infantis strain CFSAN024778 chromosome, complete genome	1779	1779	100%	0.0	97.50%	CP074257.1
Salmonella enterica subsp. enterica serovar Infantis strain CFSAN024781 chromosome, complete genome	1779	1779	100%	0.0	97.50%	CP074256.1
Salmonella enterica subsp. enterica serovar Kentucky strain CFSAN027385 chromosome, complete genome	1779	1779	100%	0.0	97.50%	CP074247.1
Salmonella enterica subsp. enterica serovar Kentucky strain CFSAN027395 chromosome, complete genome	1779	1779	100%	0.0	97.50%	CP074246.1
Salmonella enterica subsp. enterica serovar Kentucky strain CFSAN028527 chromosome, complete genome	1779	1779	100%	0.0	97.50%	CP074242.1
Salmonella enterica subsp. enterica serovar Choleraesuis str. CFSAN000514 strain SGSC 2462 isolate SARB5 chromosome, complete genome	1779	1779	100%	0.0	97.50%	CP074231.1
Salmonella enterica subsp. enterica serovar Bareilly str. CFSAN000755 chromosome, complete genome	1779	1779	100%	0.0	97.50%	CP074208.1
Salmonella enterica strain CFSAN072779 isolate 8 chromosome, complete genome	1779	1779	100%	0.0	97.50%	CP073107.1
Salmonella enterica subsp. enterica serovar Infantis strain 114061 chromosome, complete genome	1779	1779	100%	0.0	97.50%	CP070302.1
Salmonella enterica subsp. enterica serovar Infantis strain 91264 chromosome, complete genome	1779	1779	100%	0.0	97.50%	CP070301.1
Salmonella enterica subsp. enterica serovar Napoli strain 16-174478 chromosome, complete genome	1779	1779	100%	0.0	97.50%	CP063140.1
Salmonella enterica strain UWI_L96 isolate CFSAN103828 chromosome, complete genome	1779	1779	100%	0.0	97.50%	CP066318.1
Salmonella enterica strain UWI_PP98 isolate CFSAN103862 chromosome, complete genome	1779	1779	100%	0.0	97.50%	CP066321.1
Salmonella enterica strain UWI_PP31 isolate CFSAN103816 chromosome	1779	1779	100%	0.0	97.50%	CP066324.1
Salmonella enterica subsp. enterica serovar Infantis strain CFSAN103796 chromosome, complete genome	1779	1779	100%	0.0	97.50%	CP066335.1
Salmonella enterica subsp. enterica serovar Krefeld strain CVM 20739 chromosome	1779	1779	100%	0.0	97.50%	CP051455.1
Salmonella enterica subsp. enterica serovar Bredeney strain CVM 24358 chromosome	1779	1779	100%	0.0	97.50%	CP051441.1

Description	Max Score	Total Score	Query Cover	E value	Per. Ident	Accession
Salmonella enterica subsp. enterica serovar Schwarzengrund strain CVM 30168 chromosome, complete genome	1779	1779	100%	0.0	97.50%	CP051350.1
Salmonella enterica subsp. enterica serovar Kentucky strain CVM 30177 chromosome, complete genome	1779	1779	100%	0.0	97.50%	CP051346.1
Salmonella enterica strain SLR1_7966 chromosome, complete genome	1779	1779	100%	0.0	97.50%	CP060512.1
Salmonella enterica subsp. enterica serovar Infantis strain VNSEC001 chromosome, complete genome	1779	1779	100%	0.0	97.50%	CP039445.1
Salmonella enterica subsp. enterica serovar Infantis strain VNSEC002 chromosome, complete genome	1779	1779	100%	0.0	97.50%	CP039443.1
Salmonella enterica subsp. enterica serovar Infantis strain VNSEC003 chromosome, complete genome	1779	1779	100%	0.0	97.50%	CP039441.1
Salmonella enterica subsp. enterica serovar Schwarzengrund str. CVM19633, complete genome	1779	1779	100%	0.0	97.50%	CP001127.1
Salmonella enterica subsp. enterica serovar Mbandaka strain SMEH chromosome, complete genome	1777	1777	100%	0.0	97.50%	CP101689.1
Salmonella enterica subsp. enterica serovar Kentucky strain ZTA19/00815 chromosome, complete genome	1777	1777	100%	0.0	97.50%	CP089793.1
Salmonella enterica strain LHICA_E1 chromosome, complete genome	1775	1775	100%	0.0	97.41%	CP065419.1
Salmonella enterica subsp. enterica serovar Worthington strain OLF-FSR1_WB_Quail_SW-70 chromosome, complete genome	1773	1773	100%	0.0	97.41%	CP051270.1
Salmonella enterica subsp. enterica serovar Heidelberg strain CVM N16S321 chromosome, complete genome	1773	1773	100%	0.0	97.41%	CP049313.1
Salmonella enterica subsp. enterica serovar Heidelberg strain CVM N53023 chromosome, complete genome	1773	1773	100%	0.0	97.41%	CP049310.1
Salmonella enterica subsp. enterica serovar Heidelberg strain CVM N58631 chromosome, complete genome	1773	1773	100%	0.0	97.41%	CP049306.1
Salmonella enterica subsp. enterica serovar Agona strain SG17-135 chromosome, complete genome	1773	1773	100%	0.0	97.41%	CP048775.1
Salmonella enterica subsp. enterica serovar Heidelberg str. 41578 chromosome, complete genome	1773	1773	100%	0.0	97.41%	CP045762.1
Salmonella enterica subsp. enterica serovar Heidelberg strain AR-0404 chromosome, complete genome	1773	1773	100%	0.0	97.41%	CP044181.1
Salmonella enterica subsp. enterica serovar Heidelberg strain SL-312 chromosome, complete genome	1773	1773	100%	0.0	97.41%	CP043214.1
Salmonella enterica subsp. enterica serovar Agona str. 392869-2 chromosome, complete genome	1773	1773	100%	0.0	97.41%	CP015024.2
Salmonella enterica subsp. enterica serovar Agona str. 460004 2-1, complete genome	1773	1773	100%	0.0	97.41%	CP011259.2
Salmonella enterica subsp. enterica serovar Agona strain USDA-ARS-USMARC-76334 chromosome, complete genome	1773	1773	100%	0.0	97.41%	CP025454.1
Salmonella enterica subsp. enterica serovar Agona strain USDA-ARS-USMARC-76341 chromosome, complete genome	1773	1773	100%	0.0	97.41%	CP025453.1

Description	Max Score	Total Score	Query Cover	E value	Per. Ident	Accession
Salmonella enterica subsp. enterica serovar Agona strain USDA-ARS-USMARC-76340 chromosome, complete genome	1773	1773	100%	0.0	97.41%	CP025452.1
Salmonella enterica subsp. enterica serovar Agona strain USDA-ARS-USMARC-76339 chromosome, complete genome	1773	1773	100%	0.0	97.41%	CP025451.1
Salmonella enterica subsp. enterica serovar Agona strain USDA-ARS-USMARC-76338 chromosome, complete genome	1773	1773	100%	0.0	97.41%	CP025450.1
Salmonella enterica subsp. enterica serovar Agona strain USDA-ARS-USMARC-76337 chromosome, complete genome	1773	1773	100%	0.0	97.41%	CP025449.1
Salmonella enterica subsp. enterica serovar Agona strain USDA-ARS-USMARC-76336 chromosome, complete genome	1773	1773	100%	0.0	97.41%	CP025448.1
Salmonella enterica subsp. enterica serovar Agona strain USDA-ARS-USMARC-76335 chromosome, complete genome	1773	1773	100%	0.0	97.41%	CP025447.1
Salmonella enterica subsp. enterica serovar Agona strain USDA-ARS-USMARC-76333 chromosome, complete genome	1773	1773	100%	0.0	97.41%	CP025446.1
Salmonella enterica subsp. enterica serovar Agona strain USDA-ARS-USMARC-76332 chromosome, complete genome	1773	1773	100%	0.0	97.41%	CP025445.1
Salmonella enterica strain CFSAN005645 chromosome, complete genome	1773	1773	100%	0.0	97.41%	CP040380.1
Salmonella enterica subsp. enterica serovar Montevideo strain FCC0123 chromosome, complete genome	1773	1773	100%	0.0	97.41%	CP040379.1
Salmonella enterica subsp. enterica serovar Worthington strain 7102.58 chromosome, complete genome	1773	1773	100%	0.0	97.41%	CP039509.1
Salmonella enterica subsp. enterica serovar Worthington strain 7101.67 chromosome, complete genome	1773	1773	100%	0.0	97.41%	CP039503.1
Salmonella enterica subsp. enterica serovar Heidelberg strain UFPRLABMOR1 chromosome, complete genome	1773	1773	100%	0.0	97.41%	CP020101.1
Salmonella enterica subsp. enterica serovar Montevideo str. 42N chromosome, complete genome	1773	1773	100%	0.0	97.41%	CP037893.1
Salmonella enterica subsp. enterica strain NCTC6017 genome assembly, chromosome: 1	1773	1773	100%	0.0	97.41%	LR134145.1
Salmonella enterica subsp. enterica serovar Sanjuan strain NCTC7406 genome assembly, chromosome: 1	1773	1773	100%	0.0	97.41%	LR134142.1
Salmonella enterica subsp. enterica serovar Montevideo strain 11TTUC-046 chromosome, complete genome	1773	1773	100%	0.0	97.41%	CP032816.1
Salmonella enterica subsp. enterica serovar Heidelberg strain CFSAN067218 chromosome, complete genome	1773	1773	100%	0.0	97.41%	CP028311.1
Salmonella enterica subsp. enterica serovar Heidelberg strain 5 chromosome, complete genome	1773	1773	100%	0.0	97.41%	CP031359.1
Salmonella enterica strain SA20083039 chromosome, complete genome	1773	1773	100%	0.0	97.41%	CP030223.1
Salmonella enterica subsp. enterica serovar Montevideo str. CDC 2013K-0218 chromosome, complete genome	1773	1773	100%	0.0	97.41%	CP017978.1
Salmonella enterica subsp. enterica serovar Montevideo str. CDC 2012K-1544 chromosome, complete genome	1773	1773	100%	0.0	97.41%	CP017977.1

Description	Max Score	Total Score	Query Cover	E value	Per. Ident	Accession
Salmonella enterica subsp. enterica serovar Montevideo str. CDC 2011K-1674 chromosome, complete genome	1773	1773	100%	0.0	97.41%	CP017976.1
Salmonella enterica subsp. enterica serovar Montevideo str. CDC 08-1942 chromosome, complete genome	1773	1773	100%	0.0	97.41%	CP017975.1
Salmonella enterica subsp. enterica serovar Montevideo str. CDC 07-0954 chromosome, complete genome	1773	1773	100%	0.0	97.41%	CP017974.1
Salmonella enterica subsp. enterica serovar Montevideo str. USDA-ARS-USMARC-1912 chromosome, complete genome	1773	1773	100%	0.0	97.41%	CP017973.1
Salmonella enterica subsp. enterica serovar Montevideo str. USDA-ARS-USMARC-1904 chromosome, complete genome	1773	1773	100%	0.0	97.41%	CP017972.1
Salmonella enterica subsp. enterica serovar Montevideo str. USDA-ARS-USMARC-1901 chromosome, complete genome	1773	1773	100%	0.0	97.41%	CP017971.1
Salmonella enterica subsp. enterica serovar Montevideo str. USDA-ARS-USMARC-1900 chromosome, complete genome	1773	1773	100%	0.0	97.41%	CP017970.1
Salmonella enterica subsp. enterica serovar Montevideo strain CFSAN051296 chromosome, complete genome	1773	1773	100%	0.0	97.41%	CP029336.1
Salmonella enterica subsp. enterica serovar Worthington strain CFSAN051295 chromosome, complete genome	1773	1773	100%	0.0	97.41%	CP029041.1
Salmonella enterica subsp. enterica serovar Montevideo strain CFSAN045764 chromosome, complete genome	1773	1773	100%	0.0	97.41%	CP029039.1
Salmonella enterica subsp. enterica serovar Montevideo str. 531954 chromosome, complete genome	1773	1773	100%	0.0	97.41%	CP029035.1
Salmonella enterica subsp. enterica serovar Montevideo str. CDC 2010K-0257 chromosome, complete genome	1773	1773	100%	0.0	97.41%	CP020912.1
Salmonella enterica subsp. enterica serovar Montevideo str. CDC 2009K-0792 chromosome, complete genome	1773	1773	100%	0.0	97.41%	CP020752.1
Salmonella enterica subsp. enterica serovar Heidelberg strain FDAARGOS_319 chromosome, complete genome	1773	1773	100%	0.0	97.41%	CP027412.1
Salmonella enterica subsp. enterica serovar Heidelberg strain FDAARGOS_54 chromosome, complete genome	1773	1773	100%	0.0	97.41%	CP026976.1
Salmonella enterica subsp. enterica strain RM11065 chromosome, complete genome	1773	1773	100%	0.0	97.41%	CP022663.1
Salmonella enterica subsp. enterica serovar Derby strain SA20035215 chromosome, complete genome	1773	1773	100%	0.0	97.41%	CP022494.1
Salmonella enterica subsp. enterica serovar Montevideo str. USDA-ARS-USMARC-1921, complete genome	1773	1773	100%	0.0	97.41%	CP007540.2
Salmonella enterica subsp. enterica serovar Nitra strain S-1687, complete genome	1773	1773	100%	0.0	97.41%	CP019416.1
Salmonella enterica subsp. enterica serovar Crossness str. 1422-74, complete genome	1773	1773	100%	0.0	97.41%	CP019408.1
Salmonella enterica subsp. enterica serovar Borreze str. SA20041063, complete genome	1773	1773	100%	0.0	97.41%	CP019407.1
Salmonella enterica subsp. enterica serovar Heidelberg str. SARA35 chromosome, complete genome	1773	1773	100%	0.0	97.41%	CP019176.1

Description	Max Score	Total Score	Query Cover	E value	Per. Ident	Accession
Salmonella enterica subsp. enterica serovar Heidelberg strain SH13-004 chromosome, complete genome	1773	1773	100%	0.0	97.41%	CP016586.1
Salmonella enterica subsp. enterica serovar Heidelberg strain SH14-009 chromosome, complete genome	1773	1773	100%	0.0	97.41%	CP016581.1
Salmonella enterica subsp. enterica serovar Heidelberg strain SH13-006 chromosome, complete genome	1773	1773	100%	0.0	97.41%	CP016579.1
Salmonella enterica subsp. enterica serovar Heidelberg strain AMR588-04-00437 chromosome, complete genome	1773	1773	100%	0.0	97.41%	CP016576.1
Salmonella enterica subsp. enterica serovar Heidelberg strain AMR588-04-00435 chromosome, complete genome	1773	1773	100%	0.0	97.41%	CP016573.1
Salmonella enterica subsp. enterica serovar Heidelberg strain AMR588-04-00320 chromosome, complete genome	1773	1773	100%	0.0	97.41%	CP016569.1
Salmonella enterica subsp. enterica serovar Heidelberg strain AMR588-04-00318 chromosome, complete genome	1773	1773	100%	0.0	97.41%	CP016565.1
Salmonella enterica subsp. enterica serovar Heidelberg strain A3EZ223 chromosome, complete genome	1773	1773	100%	0.0	97.41%	CP016563.1
Salmonella enterica subsp. enterica serovar Heidelberg strain A3ES40 chromosome, complete genome	1773	1773	100%	0.0	97.41%	CP016561.1
Salmonella enterica subsp. enterica serovar Heidelberg strain 09-036813-1A chromosome, complete genome	1773	1773	100%	0.0	97.41%	CP016525.1
Salmonella enterica subsp. enterica serovar Heidelberg strain SA01AB09084001 chromosome, complete genome	1773	1773	100%	0.0	97.41%	CP016530.1
Salmonella enterica subsp. enterica serovar Heidelberg strain SA02DT09004001 chromosome, complete genome	1773	1773	100%	0.0	97.41%	CP016521.1
Salmonella enterica subsp. enterica serovar Heidelberg strain CE-R2-11-0435 chromosome, complete genome	1773	1773	100%	0.0	97.41%	CP016517.1
Salmonella enterica subsp. enterica serovar Heidelberg strain 11-004736-1-7 chromosome, complete genome	1773	1773	100%	0.0	97.41%	CP016514.1
Salmonella enterica subsp. enterica serovar Heidelberg strain SH14-028 chromosome, complete genome	1773	1773	100%	0.0	97.41%	CP016510.1
Salmonella enterica subsp. enterica serovar Heidelberg strain SH12-003 chromosome, complete genome	1773	1773	100%	0.0	97.41%	CP016507.1
Salmonella enterica subsp. enterica serovar Heidelberg strain SH12-007 chromosome, complete genome	1773	1773	100%	0.0	97.41%	CP016504.1
Salmonella enterica subsp. enterica serovar Heidelberg strain N13-01290, complete genome	1773	1773	100%	0.0	97.41%	CP012930.1
Salmonella enterica subsp. enterica serovar Heidelberg strain 12-4374, complete genome	1773	1773	100%	0.0	97.41%	CP012924.1
Salmonella enterica subsp. enterica serovar Heidelberg strain SA02DT10168701, complete genome	1773	1773	100%	0.0	97.41%	CP012921.1
Salmonella enterica subsp. enterica serovar Montevideo str. USDA-ARS-USMARC-1903, complete genome	1773	1773	100%	0.0	97.41%	CP007222.1
Salmonella enterica subsp. enterica serovar Agona strain R18.1477 chromosome, complete genome	1773	1773	100%	0.0	97.41%	CP100736.1

Description	Max Score	Total Score	Query Cover	E value	Per. Ident	Accession
Salmonella enterica subsp. enterica serovar Agona strain R18.2256 chromosome, complete genome	1773	1773	100%	0.0	97.41%	CP100698.1
Salmonella enterica subsp. enterica serovar Montevideo strain R17.4849 chromosome, complete genome	1773	1773	100%	0.0	97.41%	CP100747.1
Salmonella enterica subsp. enterica serovar Agona strain R21.1368 chromosome, complete genome	1773	1773	100%	0.0	97.41%	CP093405.1
Salmonella enterica subsp. enterica serovar Agona strain R18.0246 chromosome, complete genome	1773	1773	100%	0.0	97.41%	CP093411.1
Salmonella enterica subsp. enterica serovar Agona strain R21.1436 chromosome, complete genome	1773	1773	100%	0.0	97.41%	CP093403.1
Salmonella enterica subsp. enterica serovar Agona strain R19.0144 chromosome, complete genome	1773	1773	100%	0.0	97.41%	CP093407.1
Salmonella enterica subsp. enterica serovar Agona strain R21.0464 chromosome, complete genome	1773	1773	100%	0.0	97.41%	CP093402.1
Salmonella enterica subsp. enterica serovar Agona strain 18-SA00377-0 chromosome, complete genome	1773	1773	100%	0.0	97.41%	CP071388.1
Salmonella enterica subsp. enterica serovar Agona strain R21.2430 chromosome, complete genome	1773	1773	100%	0.0	97.41%	CP090930.1
Salmonella enterica subsp. enterica serovar Agona strain R21.2429 chromosome, complete genome	1773	1773	100%	0.0	97.41%	CP090929.1
Salmonella enterica subsp. enterica serovar Abony str. 0014, complete genome	1773	1773	100%	0.0	97.41%	CP007534.1
Salmonella enterica subsp. enterica serovar Montevideo str. 507440-20, complete genome	1773	1773	100%	0.0	97.41%	CP007530.1
Salmonella enterica subsp. enterica serovar 4,[5],12:i:- strain CVM N18S0736 chromosome, complete genome	1773	1773	100%	0.0	97.41%	CP082588.1
Salmonella enterica subsp. enterica serovar Agona strain CVM N18S0722 chromosome, complete genome	1773	1773	100%	0.0	97.41%	CP082595.1
Salmonella enterica subsp. enterica serovar Muenchen strain CVM N18S0476 chromosome, complete genome	1773	1773	100%	0.0	97.41%	CP082684.1
Salmonella enterica subsp. enterica serovar Agona strain CVM N18S0017 chromosome, complete genome	1773	1773	100%	0.0	97.41%	CP082615.1
Salmonella enterica subsp. enterica serovar Heidelberg strain CVM N17S1352 chromosome, complete genome	1773	1773	100%	0.0	97.41%	CP082629.1
Salmonella enterica subsp. enterica serovar 4,[5],12:i:- strain CVM N17S166 chromosome, complete genome	1773	1773	100%	0.0	97.41%	CP082668.1
Salmonella enterica subsp. enterica serovar Heidelberg strain CVM N16S098 chromosome, complete genome	1773	1773	100%	0.0	97.41%	CP082658.1
Salmonella enterica subsp. enterica serovar Agona strain FSIS1702507 chromosome, complete genome	1773	1773	100%	0.0	97.41%	CP082488.1
Salmonella enterica subsp. enterica serovar Heidelberg strain CVM N16S074 chromosome, complete genome	1773	1773	100%	0.0	97.41%	CP082736.1
Salmonella enterica subsp. enterica serovar Heidelberg strain FSIS11706780 chromosome, complete genome	1773	1773	100%	0.0	97.41%	CP082394.1

Description	Max Score	Total Score	Query Cover	E value	Per. Ident	Accession
Salmonella enterica subsp. enterica serovar Agona strain FSIS1608547 chromosome, complete genome	1773	1773	100%	0.0	97.41%	CP082431.1
Salmonella enterica subsp. enterica serovar Heidelberg strain FSIS11704575 chromosome, complete genome	1773	1773	100%	0.0	97.41%	CP082415.1
Salmonella enterica subsp. enterica serovar Agona strain FSIS11705512 chromosome, complete genome	1773	1773	100%	0.0	97.41%	CP082409.1
Salmonella enterica subsp. enterica serovar Montevideo strain FSIS1608368 chromosome, complete genome	1773	1773	100%	0.0	97.41%	CP082436.1
Salmonella enterica subsp. enterica serovar Montevideo strain FSIS1607968 chromosome, complete genome	1773	1773	100%	0.0	97.41%	CP082449.1
Salmonella enterica subsp. enterica serovar Montevideo strain FSIS1607862 chromosome, complete genome	1773	1773	100%	0.0	97.41%	CP082451.1
Salmonella enterica subsp. enterica serovar Agona strain FSIS1607113 chromosome, complete genome	1773	1773	100%	0.0	97.41%	CP082482.1
Salmonella enterica subsp. enterica serovar Agona strain FSIS1607563 chromosome, complete genome	1773	1773	100%	0.0	97.41%	CP082456.1
Salmonella enterica subsp. enterica serovar Montevideo strain FSIS1607386 chromosome, complete genome	1773	1773	100%	0.0	97.41%	CP082500.1
Salmonella enterica subsp. enterica serovar Heidelberg strain FSIS1606640 chromosome, complete genome	1773	1773	100%	0.0	97.41%	CP082503.1
Salmonella enterica subsp. enterica serovar Heidelberg str. CFSAN002064, complete genome	1773	1773	100%	0.0	97.41%	CP005995.1
Salmonella enterica subsp. enterica serovar Agona str. 24249, complete genome	1773	1773	100%	0.0	97.41%	CP006876.1
Salmonella enterica subsp. enterica serovar Heidelberg str. CFSAN002069, complete genome	1773	1773	100%	0.0	97.41%	CP005390.2
Salmonella enterica subsp. enterica serovar Heidelberg strain SH-2813-Parental 2 chromosome, complete genome	1773	1773	100%	0.0	97.41%	CP066851.1
Salmonella enterica subsp. enterica serovar Borreze strain Colony528 chromosome	1773	1773	100%	0.0	97.41%	CP078534.1
Salmonella enterica subsp. enterica serovar Heidelberg str. 41578, complete genome	1773	1773	100%	0.0	97.41%	CP004086.1
Salmonella enterica strain CFSAN012373 chromosome, complete genome	1773	1773	100%	0.0	97.41%	CP075043.1
Salmonella enterica subsp. enterica serovar Montevideo strain CFSAN008715 chromosome, complete genome	1773	1773	100%	0.0	97.41%	CP074666.1
Salmonella enterica subsp. enterica strain CFSAN029662 chromosome, complete genome	1773	1773	100%	0.0	97.41%	CP074647.1
Salmonella enterica subsp. enterica serovar Montevideo strain CFSAN001068 chromosome, complete genome	1773	1773	100%	0.0	97.41%	CP074589.1
Salmonella enterica subsp. enterica serovar Montevideo strain CFSAN012296 chromosome, complete genome	1773	1773	100%	0.0	97.41%	CP074338.1
Salmonella enterica subsp. enterica serovar Montevideo str. 4441 H chromosome, complete genome	1773	1773	100%	0.0	97.41%	CP074322.1
Salmonella enterica subsp. enterica serovar Braenderup strain CFSAN006217 chromosome, complete genome	1773	1773	100%	0.0	97.41%	CP074284.1

Description	Max Score	Total Score	Query Cover	E value	Per. Ident	Accession
Salmonella enterica subsp. enterica serovar Agona strain CFSAN006264 chromosome, complete genome	1773	1773	100%	0.0	97.41%	CP074278.1
Salmonella enterica subsp. enterica serovar Braenderup strain CFSAN006197 chromosome, complete genome	1773	1773	100%	0.0	97.41%	CP074290.1
Salmonella enterica subsp. enterica serovar Muenster str. CFSAN004344 chromosome, complete genome	1773	1773	100%	0.0	97.41%	CP074304.1
Salmonella enterica subsp. enterica serovar Braenderup strain CFSAN027384 chromosome, complete genome	1773	1773	100%	0.0	97.41%	CP074249.1
Salmonella enterica subsp. enterica serovar Montevideo strain CFSAN028508 chromosome, complete genome	1773	1773	100%	0.0	97.41%	CP074244.1
Salmonella enterica subsp. enterica serovar Derby str. CFSAN000565 strain SGSC 2468 isolate SARB11 chromosome, complete genome	1773	1773	100%	0.0	97.41%	CP074219.1
Salmonella enterica subsp. enterica serovar Heidelberg str. SARA37 strain SGSC 2217 chromosome, complete genome	1773	1773	100%	0.0	97.41%	CP074234.1
Salmonella enterica strain FDAARGOS_1271 chromosome, complete genome	1773	1773	100%	0.0	97.41%	CP069518.1
Salmonella enterica subsp. enterica serovar Weltevreden strain colony_81 chromosome	1773	1773	100%	0.0	97.41%	CP067979.1
Salmonella enterica subsp. enterica serovar Heidelberg strain CVM 20760 chromosome, complete genome	1773	1773	100%	0.0	97.41%	CP051410.1
Salmonella enterica subsp. enterica serovar Agona strain CVM 21974 chromosome, complete genome	1773	1773	100%	0.0	97.41%	CP051402.1
Salmonella enterica subsp. enterica serovar Heidelberg strain CVM 24359 chromosome, complete genome	1773	1773	100%	0.0	97.41%	CP051381.1
Salmonella enterica subsp. enterica serovar Heidelberg strain CVM 28322 chromosome, complete genome	1773	1773	100%	0.0	97.41%	CP051358.1
Salmonella enterica subsp. enterica serovar Agona strain CVM 28336 chromosome, complete genome	1773	1773	100%	0.0	97.41%	CP051356.1
Salmonella enterica subsp. enterica serovar Worthington strain CVM 30148 chromosome, complete genome	1773	1773	100%	0.0	97.41%	CP051353.1
Salmonella enterica subsp. enterica serovar Derby strain CVM 30155 chromosome, complete genome	1773	1773	100%	0.0	97.41%	CP053048.1
Salmonella enterica subsp. enterica serovar Worthington strain CVM 33836 chromosome, complete genome	1773	1773	100%	0.0	97.41%	CP051338.1
Salmonella enterica subsp. enterica serovar Heidelberg strain CVM 35161 chromosome, complete genome	1773	1773	100%	0.0	97.41%	CP051310.1
Salmonella enterica subsp. enterica serovar Heidelberg str. B182, complete genome	1773	1773	100%	0.0	97.41%	CP003416.1
Salmonella enterica subsp. enterica serovar Agona str. SL483, complete genome	1773	1773	100%	0.0	97.41%	CP001138.1
Salmonella enterica subsp. enterica serovar Heidelberg str. SL476, complete genome	1773	1773	100%	0.0	97.41%	CP001120.1
Salmonella enterica subsp. enterica serovar Senftenberg strain N17-509 chromosome, complete genome	1772	1772	100%	0.0	97.41%	CP026379.1

Description	Max Score	Total Score	Query Cover	E value	Per. Ident	Accession
Salmonella enterica subsp. enterica serovar Senftenberg str. ATCC 43845, complete genome	1772	1772	100%	0.0	97.41%	CP019194.1
Salmonella enterica subsp. enterica serovar Typhi strain CMCST_CEPR_1 chromosome, complete genome	1768	1768	100%	0.0	97.31%	CP053702.1
Salmonella enterica strain FDAARGOS_718 chromosome	1768	1768	100%	0.0	97.31%	CP054901.1
Salmonella enterica subsp. enterica strain LHST_2018 chromosome, complete genome	1768	1768	100%	0.0	97.31%	CP052767.1
Salmonella enterica subsp. enterica serovar Typhi strain R19.2839 chromosome, complete genome	1768	1768	100%	0.0	97.31%	CP046429.1
Salmonella enterica subsp. enterica serovar Typhi strain 2018K-0756 chromosome, complete genome	1768	1768	100%	0.0	97.31%	CP044007.1
Salmonella enterica subsp. enterica serovar Braenderup strain FORC93 chromosome, complete genome	1768	1768	100%	0.0	97.31%	CP032304.1
Salmonella enterica subsp. enterica serovar Javiana strain FARPER-220 chromosome, complete genome	1768	1768	100%	0.0	97.31%	CP038233.1
Salmonella enterica subsp. enterica serovar Typhi strain WGS1146 chromosome, complete genome	1768	1768	100%	0.0	97.31%	CP040575.1
Salmonella enterica subsp. enterica serovar Typhi str. Ty2 strain 4316STDY6559672 genome assembly, chromosome: 1	1768	1768	100%	0.0	97.31%	LR590082.1
Salmonella enterica subsp. enterica serovar Typhi str. Ty2 strain 4316STDY6559669 genome assembly, chromosome: 1	1768	1768	100%	0.0	97.31%	LR590081.1
Salmonella enterica subsp. enterica serovar Typhi strain 311189_217186 chromosome, complete genome	1768	1768	100%	0.0	97.31%	CP029646.1
Salmonella enterica subsp. enterica serovar Typhi strain 311189_201186 chromosome, complete genome	1768	1768	100%	0.0	97.31%	CP029958.1
Salmonella enterica subsp. enterica serovar Typhi strain 311189_218186 chromosome, complete genome	1768	1768	100%	0.0	97.31%	CP029925.1
Salmonella enterica subsp. enterica serovar Typhi strain 343078_273110 chromosome, complete genome	1768	1768	100%	0.0	97.31%	CP029846.1
Salmonella enterica subsp. enterica serovar Typhi strain 343078_256191 chromosome, complete genome	1768	1768	100%	0.0	97.31%	CP029959.1
Salmonella enterica subsp. enterica serovar Typhi strain 343078_251131 chromosome, complete genome	1768	1768	100%	0.0	97.31%	CP029960.1
Salmonella enterica subsp. enterica serovar Typhi strain 343078_228140 chromosome, complete genome	1768	1768	100%	0.0	97.31%	CP029962.1
Salmonella enterica subsp. enterica serovar Typhi strain 343078_223175 chromosome, complete genome	1768	1768	100%	0.0	97.31%	CP029964.1
Salmonella enterica subsp. enterica serovar Typhi strain 343078_211126 chromosome, complete genome	1768	1768	100%	0.0	97.31%	CP029848.1
Salmonella enterica subsp. enterica serovar Typhi strain 343078_203125 chromosome, complete genome	1768	1768	100%	0.0	97.31%	CP029850.1
Salmonella enterica subsp. enterica serovar Typhi strain 343078_201101 chromosome, complete genome	1768	1768	100%	0.0	97.31%	CP029852.1

Description	Max Score	Total Score	Query Cover	E value	Per. Ident	Accession
Salmonella enterica subsp. enterica serovar Typhi strain 343077_292148 chromosome, complete genome	1768	1768	100%	0.0	97.31%	CP029855.1
Salmonella enterica subsp. enterica serovar Typhi strain 343077_286126 chromosome, complete genome	1768	1768	100%	0.0	97.31%	CP029856.1
Salmonella enterica subsp. enterica serovar Typhi strain 343077_285138 chromosome, complete genome	1768	1768	100%	0.0	97.31%	CP029858.1
Salmonella enterica subsp. enterica serovar Typhi strain 343077_281186 chromosome, complete genome	1768	1768	100%	0.0	97.31%	CP029853.1
Salmonella enterica subsp. enterica serovar Typhi strain 343077_278127 chromosome, complete genome	1768	1768	100%	0.0	97.31%	CP029863.1
Salmonella enterica subsp. enterica serovar Typhi strain 343077_267164 chromosome, complete genome	1768	1768	100%	0.0	97.31%	CP029906.1
Salmonella enterica subsp. enterica serovar Typhi strain 343077_260153 chromosome, complete genome	1768	1768	100%	0.0	97.31%	CP029861.1
Salmonella enterica subsp. enterica serovar Typhi strain 343077_255118 chromosome, complete genome	1768	1768	100%	0.0	97.31%	CP029907.1
Salmonella enterica subsp. enterica serovar Typhi strain 343077_228157 chromosome, complete genome	1768	1768	100%	0.0	97.31%	CP029864.1
Salmonella enterica subsp. enterica serovar Typhi strain 343077_228140 chromosome, complete genome	1768	1768	100%	0.0	97.31%	CP029866.1
Salmonella enterica subsp. enterica serovar Typhi strain 343077_215174 chromosome, complete genome	1768	1768	100%	0.0	97.31%	CP029868.1
Salmonella enterica subsp. enterica serovar Typhi strain 343077_214162 chromosome, complete genome	1768	1768	100%	0.0	97.31%	CP029862.1
Salmonella enterica subsp. enterica serovar Typhi strain 343077_214135 chromosome, complete genome	1768	1768	100%	0.0	97.31%	CP029872.1
Salmonella enterica subsp. enterica serovar Typhi strain 343077_213147 chromosome, complete genome	1768	1768	100%	0.0	97.31%	CP029897.1
Salmonella enterica subsp. enterica serovar Typhi strain 343077_212159 chromosome, complete genome	1768	1768	100%	0.0	97.31%	CP029870.1
Salmonella enterica subsp. enterica serovar Typhi strain 343077_212138 chromosome, complete genome	1768	1768	100%	0.0	97.31%	CP029919.1
Salmonella enterica subsp. enterica serovar Typhi strain 343077_211171 chromosome, complete genome	1768	1768	100%	0.0	97.31%	CP029873.1
Salmonella enterica subsp. enterica serovar Typhi strain 343076_294172 chromosome, complete genome	1768	1768	100%	0.0	97.31%	CP029888.1
Salmonella enterica subsp. enterica serovar Typhi strain 343076_269157 chromosome, complete genome	1768	1768	100%	0.0	97.31%	CP029881.1
Salmonella enterica subsp. enterica serovar Typhi strain 343076_253155 chromosome, complete genome	1768	1768	100%	0.0	97.31%	CP029890.1
Salmonella enterica subsp. enterica serovar Typhi strain 343076_252143 chromosome, complete genome	1768	1768	100%	0.0	97.31%	CP029892.1
Salmonella enterica subsp. enterica serovar Typhi strain 343076_249107 chromosome, complete genome	1768	1768	100%	0.0	97.31%	CP029913.1

Description	Max Score	Total Score	Query Cover	E value	Per. Ident	Accession
Salmonella enterica subsp. enterica serovar Typhi strain 343076_248190 chromosome, complete genome	1768	1768	100%	0.0	97.31%	CP029882.1
Salmonella enterica subsp. enterica serovar Typhi strain 343076_241106 chromosome, complete genome	1768	1768	100%	0.0	97.31%	CP029899.1
Salmonella enterica subsp. enterica serovar Typhi strain 343076_232188 chromosome, complete genome	1768	1768	100%	0.0	97.31%	CP029900.1
Salmonella enterica subsp. enterica serovar Typhi strain 343076_227128 chromosome, complete genome	1768	1768	100%	0.0	97.31%	CP029875.1
Salmonella enterica subsp. enterica serovar Typhi strain 311189_282186 chromosome, complete genome	1768	1768	100%	0.0	97.31%	CP029920.1
Salmonella enterica subsp. enterica serovar Typhi strain 343076_217103 chromosome, complete genome	1768	1768	100%	0.0	97.31%	CP029914.1
Salmonella enterica subsp. enterica serovar Typhi strain 343076_202113 chromosome, complete genome	1768	1768	100%	0.0	97.31%	CP029915.1
Salmonella enterica subsp. enterica serovar Typhi strain 311189_291186 chromosome, complete genome	1768	1768	100%	0.0	97.31%	CP029894.1
Salmonella enterica subsp. enterica serovar Typhi strain 311189_269186 chromosome, complete genome	1768	1768	100%	0.0	97.31%	CP029922.1
Salmonella enterica subsp. enterica serovar Typhi strain 311189_268186 chromosome, complete genome	1768	1768	100%	0.0	97.31%	CP029883.1
Salmonella enterica subsp. enterica serovar Typhi strain 311189_268103 chromosome, complete genome	1768	1768	100%	0.0	97.31%	CP029902.1
Salmonella enterica subsp. enterica serovar Typhi strain 311189_256186 chromosome, complete genome	1768	1768	100%	0.0	97.31%	CP029917.1
Salmonella enterica subsp. enterica serovar Typhi strain 311189_255186 chromosome, complete genome	1768	1768	100%	0.0	97.31%	CP029885.1
Salmonella enterica subsp. enterica serovar Typhi strain 311189_252186 chromosome, complete genome	1768	1768	100%	0.0	97.31%	CP029896.1
Salmonella enterica subsp. enterica serovar Typhi strain 311189_239103 chromosome, complete genome	1768	1768	100%	0.0	97.31%	CP029908.1
Salmonella enterica subsp. enterica serovar Typhi strain 311189_232103 chromosome, complete genome	1768	1768	100%	0.0	97.31%	CP029918.1
Salmonella enterica subsp. enterica serovar Typhi strain 311189_231186 chromosome, complete genome	1768	1768	100%	0.0	97.31%	CP029904.1
Salmonella enterica subsp. enterica serovar Typhi strain 311189_224186 chromosome, complete genome	1768	1768	100%	0.0	97.31%	CP029878.1
Salmonella enterica subsp. enterica serovar Typhi strain 311189_223186 chromosome, complete genome	1768	1768	100%	0.0	97.31%	CP029880.1
Salmonella enterica subsp. enterica serovar Typhi strain 311189_222186 chromosome, complete genome	1768	1768	100%	0.0	97.31%	CP029909.1
Salmonella enterica subsp. enterica serovar Typhi strain 311189_221186 chromosome, complete genome	1768	1768	100%	0.0	97.31%	CP029923.1
Salmonella enterica subsp. enterica serovar Typhi strain 311189_220186 chromosome, complete genome	1768	1768	100%	0.0	97.31%	CP029886.1

Description	Max Score	Total Score	Query Cover	E value	Per. Ident	Accession
Salmonella enterica subsp. enterica serovar Typhi strain 311189_219186 chromosome, complete genome	1768	1768	100%	0.0	97.31%	CP029911.1
Salmonella enterica subsp. enterica serovar Typhi strain 311189_217103 chromosome, complete genome	1768	1768	100%	0.0	97.31%	CP029927.1
Salmonella enterica subsp. enterica serovar Typhi strain 311189_216103 chromosome, complete genome	1768	1768	100%	0.0	97.31%	CP029928.1
Salmonella enterica subsp. enterica serovar Typhi strain 311189_215186 chromosome, complete genome	1768	1768	100%	0.0	97.31%	CP029930.1
Salmonella enterica subsp. enterica serovar Typhi strain 311189_214186 chromosome, complete genome	1768	1768	100%	0.0	97.31%	CP029933.1
Salmonella enterica subsp. enterica serovar Typhi strain 311189_213186 chromosome, complete genome	1768	1768	100%	0.0	97.31%	CP029936.1
Salmonella enterica subsp. enterica serovar Typhi strain 311189_212186 chromosome, complete genome	1768	1768	100%	0.0	97.31%	CP029944.1
Salmonella enterica subsp. enterica serovar Typhi strain 311189_211186 chromosome, complete genome	1768	1768	100%	0.0	97.31%	CP029945.1
Salmonella enterica subsp. enterica serovar Typhi strain 311189_210186 chromosome, complete genome	1768	1768	100%	0.0	97.31%	CP029946.1
Salmonella enterica subsp. enterica serovar Typhi strain 311189_209186 chromosome, complete genome	1768	1768	100%	0.0	97.31%	CP029952.1
Salmonella enterica subsp. enterica serovar Typhi strain 311189_208186 chromosome, complete genome	1768	1768	100%	0.0	97.31%	CP029949.1
Salmonella enterica subsp. enterica serovar Typhi strain 311189_208103 chromosome, complete genome	1768	1768	100%	0.0	97.31%	CP029932.1
Salmonella enterica subsp. enterica serovar Typhi strain 311189_207186 chromosome, complete genome	1768	1768	100%	0.0	97.31%	CP029938.1
Salmonella enterica subsp. enterica serovar Typhi strain 311189_206186 chromosome, complete genome	1768	1768	100%	0.0	97.31%	CP029940.1
Salmonella enterica subsp. enterica serovar Typhi strain 311189_205186 chromosome, complete genome	1768	1768	100%	0.0	97.31%	CP029950.1
Salmonella enterica subsp. enterica serovar Typhi strain 311189_204186 chromosome, complete genome	1768	1768	100%	0.0	97.31%	CP029954.1
Salmonella enterica subsp. enterica serovar Typhi strain 311189_203186 chromosome, complete genome	1768	1768	100%	0.0	97.31%	CP029942.1
Salmonella enterica subsp. enterica serovar Typhi strain 311189_202186 chromosome, complete genome	1768	1768	100%	0.0	97.31%	CP029956.1
Salmonella enterica subsp. enterica serovar Fresno strain USMARC-69835 chromosome, complete genome	1768	1768	100%	0.0	97.31%	CP032444.1
Salmonella enterica subsp. enterica serovar Typhi strain Ty21a chromosome, complete genome	1768	1768	100%	0.0	97.31%	CP023975.1
Salmonella enterica subsp. enterica serovar Typhi strain LXYSH chromosome, complete genome	1768	1768	100%	0.0	97.31%	CP030936.1
Salmonella enterica subsp. enterica serovar Braenderup str. ATCC BAA-664 chromosome, complete genome	1768	1768	100%	0.0	97.31%	CP034773.1

Description	Max Score	Total Score	Query Cover	E value	Per. Ident	Accession
Salmonella enterica subsp. salamae serovar 42:r:- strain RSE42 chromosome, complete genome	1768	1768	100%	0.0	97.31%	CP034697.1
Salmonella enterica subsp. salamae serovar 42:r:- strain RSE09 chromosome, complete genome	1768	1768	100%	0.0	97.31%	CP034717.1
Salmonella enterica subsp. salamae serovar 42:r:- strain RSE36 chromosome, complete genome	1768	1768	100%	0.0	97.31%	CP034702.1
Salmonella enterica strain SA20083530 chromosome, complete genome	1768	1768	100%	0.0	97.31%	CP030203.1
Salmonella enterica strain NCTC9948 genome assembly, chromosome: 1	1768	1768	100%	0.0	97.31%	LS483495.1
Salmonella enterica subsp. enterica strain NCTC6480 genome assembly, chromosome: 1	1768	1768	100%	0.0	97.31%	LS483454.1
Salmonella enterica subsp. enterica serovar Gaminara strain CFSAN070644 chromosome, complete genome	1768	1768	100%	0.0	97.31%	CP024165.1
Salmonella enterica subsp. enterica serovar Typhi strain OVG_041 genome assembly, chromosome: 2	1768	1768	100%	0.0	97.31%	LT906560.1
Salmonella enterica subsp. enterica serovar Typhi strain SGB90 genome assembly, chromosome: 1	1768	1768	100%	0.0	97.31%	LT904870.2
Salmonella enterica subsp. enterica serovar Typhi isolate ISP_03_07467_SGB110-sc-1979083 genome assembly, chromosome: 1	1768	1768	100%	0.0	97.31%	LT905060.2
Salmonella enterica subsp. enterica serovar Typhi strain 1036491 genome assembly, chromosome: 1	1768	1768	100%	0.0	97.31%	LT906495.1
Salmonella enterica subsp. enterica serovar Typhi strain ERL024120 genome assembly, chromosome: 1	1768	1768	100%	0.0	97.31%	LT906494.1
Salmonella enterica subsp. enterica serovar Typhi isolate 1554-sc-2165329 genome assembly, chromosome: 1	1768	1768	100%	0.0	97.31%	LT906493.1
Salmonella enterica subsp. enterica serovar Typhi isolate E98_3139-sc-1927833 genome assembly, chromosome: 1	1768	1768	100%	0.0	97.31%	LT905143.1
Salmonella enterica subsp. enterica serovar Typhi strain H12ESR00755-001A genome assembly, chromosome: 1	1768	1768	100%	0.0	97.31%	LT905142.1
Salmonella enterica subsp. enterica serovar Typhi strain OVG_041 genome assembly, chromosome: 1	1768	1768	100%	0.0	97.31%	LT905141.1
Salmonella enterica subsp. enterica serovar Typhi strain ERL082356 genome assembly, chromosome: 1	1768	1768	100%	0.0	97.31%	LT905140.1
Salmonella enterica subsp. enterica serovar Typhi strain 2010-007898 genome assembly, chromosome: 1	1768	1768	100%	0.0	97.31%	LT905139.1
Salmonella enterica subsp. enterica serovar Typhi strain ty3-243 genome assembly, chromosome: 2	1768	1768	100%	0.0	97.31%	LT905090.1
Salmonella enterica subsp. enterica serovar Typhi strain ERL024120 genome assembly, chromosome: 1	1768	1768	100%	0.0	97.31%	LT905088.1
Salmonella enterica subsp. enterica serovar Typhi isolate 1554-sc-2165329 genome assembly, chromosome: 1	1768	1768	100%	0.0	97.31%	LT905064.1
Salmonella enterica subsp. enterica serovar Typhi isolate lupe_GEN0059-sc-1979081 genome assembly, chromosome: 1	1768	1768	100%	0.0	97.31%	LT905063.1

Description	Max Score	Total Score	Query Cover	E value	Per. Ident	Accession
Salmonella enterica subsp. enterica serovar Typhi isolate 403Ty-sc-1979084 genome assembly, chromosome: 1	1768	1768	100%	0.0	97.31%	LT905062.1
Salmonella enterica subsp. enterica serovar Typhi strain ERL12960 genome assembly, chromosome: 1	1768	1768	100%	0.0	97.31%	LT904894.1
Salmonella enterica subsp. enterica serovar Typhi strain 1016889 genome assembly, chromosome: 1	1768	1768	100%	0.0	97.31%	LT904893.1
Salmonella enterica subsp. enterica serovar Typhi strain 80-2002 genome assembly, chromosome: 1	1768	1768	100%	0.0	97.31%	LT904891.1
Salmonella enterica subsp. enterica serovar Typhi strain H12ESR00394-001A genome assembly, chromosome: 1	1768	1768	100%	0.0	97.31%	LT904890.1
Salmonella enterica subsp. enterica serovar Typhi strain 129-0238-M genome assembly, chromosome: 1	1768	1768	100%	0.0	97.31%	LT904888.1
Salmonella enterica subsp. enterica serovar Typhi strain 76-1292 genome assembly, chromosome: 1	1768	1768	100%	0.0	97.31%	LT904887.1
Salmonella enterica subsp. enterica serovar Typhi strain SGB82 genome assembly, chromosome: 1	1768	1768	100%	0.0	97.31%	LT904886.1
Salmonella enterica subsp. enterica serovar Typhi strain 1036491 genome assembly, chromosome: 1	1768	1768	100%	0.0	97.31%	LT904885.1
Salmonella enterica subsp. enterica serovar Typhi strain ERL041834 genome assembly, chromosome: 1	1768	1768	100%	0.0	97.31%	LT904884.1
Salmonella enterica subsp. enterica serovar Typhi strain ERL103914 genome assembly, chromosome: 1	1768	1768	100%	0.0	97.31%	LT904883.1
Salmonella enterica subsp. enterica serovar Typhi strain SGB89 genome assembly, chromosome: 1	1768	1768	100%	0.0	97.31%	LT904882.1
Salmonella enterica subsp. enterica serovar Typhi strain UI2120 genome assembly, chromosome: 1	1768	1768	100%	0.0	97.31%	LT904881.1
Salmonella enterica subsp. enterica serovar Typhi strain ty3-193 genome assembly, chromosome: 1	1768	1768	100%	0.0	97.31%	LT904878.1
Salmonella enterica subsp. enterica serovar Typhi strain SGB92 genome assembly, chromosome: 1	1768	1768	100%	0.0	97.31%	LT904877.1
Salmonella enterica subsp. enterica serovar Typhi strain ERL114000 genome assembly, chromosome: 1	1768	1768	100%	0.0	97.31%	LT904876.1
Salmonella enterica subsp. enterica serovar Typhi strain ERL024919 genome assembly, chromosome: 1	1768	1768	100%	0.0	97.31%	LT904875.1
Salmonella enterica subsp. enterica serovar Typhi strain ERL11909 genome assembly, chromosome: 1	1768	1768	100%	0.0	97.31%	LT904872.1
Salmonella enterica subsp. enterica serovar Typhi strain 1553 genome assembly, chromosome: 1	1768	1768	100%	0.0	97.31%	LT904871.1
Salmonella enterica subsp. enterica serovar Typhi strain ERL052042 genome assembly, chromosome: 1	1768	1768	100%	0.0	97.31%	LT904869.1
Salmonella enterica subsp. enterica serovar Typhi strain H12ESR04734-001A genome assembly, chromosome: 1	1768	1768	100%	0.0	97.31%	LT904868.1
Salmonella enterica subsp. enterica serovar Typhi strain ERL034151 genome assembly, chromosome: 1	1768	1768	100%	0.0	97.31%	LT904867.1

Description	Max Score	Total Score	Query Cover	E value	Per. Ident	Accession
Salmonella enterica subsp. enterica serovar Typhi strain M223 genome assembly, chromosome: 1	1768	1768	100%	0.0	97.31%	LT904854.1
Salmonella enterica subsp. enterica serovar Typhi strain TY585 genome assembly, chromosome: 1	1768	1768	100%	0.0	97.31%	LT904852.1
Salmonella enterica subsp. enterica serovar Braenderup strain SA20026289 chromosome, complete genome	1768	1768	100%	0.0	97.31%	CP022490.1
Salmonella enterica subsp. enterica serovar Waycross strain SA20041608, complete genome	1768	1768	100%	0.0	97.31%	CP022138.1
Salmonella enterica subsp. enterica serovar Typhi strain ERL12148 genome assembly, chromosome: 1	1768	1768	100%	0.0	97.31%	LT883153.1
Salmonella enterica subsp. enterica serovar Typhi genome assembly, chromosome: 1	1768	1768	100%	0.0	97.31%	LT882486.1
Salmonella enterica subsp. enterica serovar Hillingdon str. N1529-D3, complete genome	1768	1768	100%	0.0	97.31%	CP019410.1
Salmonella enterica subsp. enterica serovar Djakarta str. S-1087, complete genome	1768	1768	100%	0.0	97.31%	CP019409.1
Salmonella enterica subsp. enterica serovar Typhi strain B/SF/13/03/195, complete genome	1768	1768	100%	0.0	97.31%	CP012151.1
Salmonella enterica subsp. enterica serovar Typhi strain PM016/13, complete genome	1768	1768	100%	0.0	97.31%	CP012091.1
Salmonella enterica subsp. enterica serovar Typhi strain S1 chromosome, complete genome	1768	1768	100%	0.0	97.31%	CP117294.1
Salmonella enterica subsp. enterica serovar Typhi strain S2 chromosome, complete genome	1768	1768	100%	0.0	97.31%	CP117293.1
Salmonella enterica subsp. enterica serovar Typhi strain S4 chromosome, complete genome	1768	1768	100%	0.0	97.31%	CP117292.1
Salmonella enterica subsp. enterica serovar Typhi strain S5 chromosome, complete genome	1768	1768	100%	0.0	97.31%	CP117291.1
Salmonella enterica subsp. enterica serovar Typhi strain S6 chromosome, complete genome	1768	1768	100%	0.0	97.31%	CP117290.1
Salmonella enterica strain 2011K-0222 chromosome, complete genome	1768	1768	100%	0.0	97.31%	CP114279.1
Salmonella enterica strain ZYX chromosome, complete genome	1768	1768	100%	0.0	97.31%	CP113534.1
Salmonella enterica subsp. enterica serovar Typhi strain BRD948 genome assembly, chromosome: 1	1768	1768	100%	0.0	97.31%	OW707734.1
Salmonella enterica subsp. enterica serovar Typhi strain BRD948 genome assembly, chromosome: 1	1768	1768	100%	0.0	97.31%	OW706512.1
Salmonella enterica subsp. enterica serovar Typhi strain BRD948 genome assembly, chromosome: 1	1768	1768	100%	0.0	97.31%	OW704627.1
Salmonella enterica subsp. enterica serovar Typhi strain BRD948 genome assembly, chromosome: 1	1768	1768	100%	0.0	97.31%	OW704602.1
Salmonella enterica subsp. enterica serovar Typhi strain BRD948 genome assembly, chromosome: 1	1768	1768	100%	0.0	97.31%	OW704291.1
Salmonella enterica strain P201982 chromosome, complete genome	1768	1768	100%	0.0	97.31%	CP093392.1
Salmonella enterica strain P287611 chromosome, complete genome	1768	1768	100%	0.0	97.31%	CP093391.1

Description	Max Score	Total Score	Query Cover	E value	Per. Ident	Accession
Salmonella enterica strain P289597 chromosome, complete genome	1768	1768	100%	0.0	97.31%	CP093390.1
Salmonella enterica strain P281972 chromosome, complete genome	1768	1768	100%	0.0	97.31%	CP092075.1
Salmonella enterica subsp. enterica strain CFSAN023357 chromosome, complete genome	1768	1768	100%	0.0	97.31%	CP086351.1
Salmonella enterica subsp. enterica strain CFSAN023356 chromosome, complete genome	1768	1768	100%	0.0	97.31%	CP086353.1
Salmonella enterica subsp. enterica strain CFSAN008899 chromosome, complete genome	1768	1768	100%	0.0	97.31%	CP085054.1
Salmonella enterica subsp. enterica serovar Javiana strain FDA1048408-C002-006 chromosome, complete genome	1768	1768	100%	0.0	97.31%	CP085052.1
Salmonella enterica subsp. enterica serovar Typhi strain Entei chromosome, complete genome	1768	1768	100%	0.0	97.31%	CP085808.1
Salmonella enterica subsp. enterica serovar Typhi strain Suicune chromosome, complete genome	1768	1768	100%	0.0	97.31%	CP085807.1
Salmonella enterica subsp. enterica serovar Typhimurium strain Raikou chromosome, complete genome	1768	1768	100%	0.0	97.31%	CP085809.1
Salmonella enterica subsp. enterica serovar Typhi strain MDUST348 genome assembly, chromosome: 1	1768	1768	100%	0.0	97.31%	OU943338.1
Salmonella enterica subsp. enterica serovar Typhi strain MDUST255 genome assembly, chromosome: 1	1768	1768	100%	0.0	97.31%	OU943337.1
Salmonella enterica subsp. enterica serovar Typhi strain MDUST305 genome assembly, chromosome: 1	1768	1768	100%	0.0	97.31%	OU943336.1
Salmonella enterica subsp. enterica serovar Typhi strain 681355 chromosome, complete genome	1768	1768	100%	0.0	97.31%	CP083411.1
Salmonella enterica subsp. enterica serovar Bredeney strain FSIS1702037 chromosome, complete genome	1768	1768	100%	0.0	97.31%	CP082381.1
Salmonella enterica subsp. enterica serovar Typhi strain ISP2825 chromosome, complete genome	1768	1768	100%	0.0	97.31%	CP080960.1
Salmonella enterica strain CFSAN038591 chromosome, complete genome	1768	1768	100%	0.0	97.31%	CP076329.1
Salmonella enterica strain CFSAN055400 chromosome, complete genome	1768	1768	100%	0.0	97.31%	CP076098.1
Salmonella enterica strain CFSAN038580 chromosome, complete genome	1768	1768	100%	0.0	97.31%	CP076097.1
Salmonella enterica subsp. enterica strain CFSAN058588 chromosome, complete genome	1768	1768	100%	0.0	97.31%	CP077702.1
Salmonella enterica subsp. enterica serovar Typhi strain ERL072973 genome assembly, chromosome: 1	1768	1768	100%	0.0	97.31%	LT904777.2
Salmonella enterica strain CFSAN048049 chromosome, complete genome	1768	1768	100%	0.0	97.31%	CP076327.1
Salmonella enterica strain CFSAN073546 chromosome, complete genome	1768	1768	100%	0.0	97.31%	CP075105.1
Salmonella enterica strain CFSAN073554 chromosome, complete genome	1768	1768	100%	0.0	97.31%	CP075102.1
Salmonella enterica subsp. enterica serovar Tennessee strain CFSAN024441 chromosome, complete genome	1768	1768	100%	0.0	97.31%	CP075010.1

Description	Max Score	Total Score	Query Cover	E value	Per. Ident	Accession
Salmonella enterica strain CFSAN029843 chromosome, complete genome	1768	1768	100%	0.0	97.31%	CP074644.1
Salmonella enterica strain CFSAN029878 chromosome, complete genome	1768	1768	100%	0.0	97.31%	CP074632.1
Salmonella enterica strain CFSAN029882 chromosome, complete genome	1768	1768	100%	0.0	97.31%	CP074652.1
Salmonella enterica strain CFSAN029945 chromosome, complete genome	1768	1768	100%	0.0	97.31%	CP074651.1
Salmonella enterica subsp. enterica serovar Typhi strain CFSAN001004 chromosome, complete genome	1768	1768	100%	0.0	97.31%	CP074603.1
Salmonella enterica subsp. salamae strain CFSAN001016 chromosome, complete genome	1768	1768	100%	0.0	97.31%	CP074594.1
Salmonella enterica subsp. enterica serovar Typhi str. CT18, complete chromosome	1768	1768	100%	0.0	97.31%	AL513382.1
Salmonella enterica subsp. enterica serovar Typhi str. Ty21a, complete genome	1768	1768	100%	0.0	97.31%	CP002099.1
Salmonella enterica subsp. enterica serovar Javiana strain CFSAN006238 chromosome, complete genome	1768	1768	100%	0.0	97.31%	CP074281.1
Salmonella enterica subsp. enterica serovar Javiana strain CFSAN006269 chromosome, complete genome	1768	1768	100%	0.0	97.31%	CP074276.1
Salmonella enterica subsp. enterica serovar Javiana strain CFSAN006218 chromosome, complete genome	1768	1768	100%	0.0	97.31%	CP074283.1
Salmonella enterica subsp. enterica serovar Irumu strain CFSAN024579 chromosome, complete genome	1768	1768	100%	0.0	97.31%	CP074270.1
Salmonella enterica subsp. enterica serovar Itami strain CFSAN024577 chromosome, complete genome	1768	1768	100%	0.0	97.31%	CP074272.1
Salmonella enterica subsp. enterica serovar Potsdam strain CFSAN029622 chromosome, complete genome	1768	1768	100%	0.0	97.31%	CP074241.1
Salmonella enterica subsp. enterica serovar Javiana str. CFSAN000905 chromosome, complete genome	1768	1768	100%	0.0	97.31%	CP074206.1
Salmonella enterica subsp. enterica serovar Havana str. CFSAN001082 chromosome, complete genome	1768	1768	100%	0.0	97.31%	CP074203.1
Salmonella enterica subsp. enterica serovar Javiana strain CFSAN001070 chromosome, complete genome	1768	1768	100%	0.0	97.31%	CP074205.1
Salmonella enterica strain Colony14 chromosome	1768	1768	100%	0.0	97.31%	CP070325.1
Salmonella enterica subsp. enterica serovar Javiana str. CFSAN001992, complete genome	1768	1768	100%	0.0	97.31%	CP004027.1
Salmonella enterica strain colony_14 chromosome	1768	1768	100%	0.0	97.31%	CP067978.1
Salmonella enterica strain UWI_PP122 isolate CFSAN103840 chromosome, complete genome	1768	1768	100%	0.0	97.31%	CP066325.1
Salmonella enterica subsp. enterica serovar Typhi str. P-stx-12, complete genome	1768	1768	100%	0.0	97.31%	CP003278.1
Salmonella enterica subsp. enterica serovar Typhi Ty2, complete genome	1768	1768	100%	0.0	97.31%	AE014613.1
Salmonella enterica subsp. enterica serovar Johannesburg strain CVM N58011 chromosome, complete genome	1762	1762	100%	0.0	97.21%	CP049308.1
Salmonella enterica strain CFSAN079101 chromosome, complete genome	1762	1762	100%	0.0	97.22%	CP042442.1

Description	Max Score	Total Score	Query Cover	E value	Per. Ident	Accession
Salmonella enterica strain CFSAN079094 chromosome, complete genome	1762	1762	100%	0.0	97.21%	CP042438.1
Salmonella enterica subsp. enterica serovar Weltevreden strain 3 isolate CFSAN047349 chromosome, complete genome	1762	1762	100%	0.0	97.21%	CP040701.1
Salmonella enterica subsp. enterica serovar Johannesburg strain USDA-ARS-USMARC-60984 chromosome, complete genome	1762	1762	100%	0.0	97.21%	CP025276.1
Salmonella enterica subsp. enterica strain NCTC129 genome assembly, chromosome: 1	1762	1762	100%	0.0	97.21%	LR134140.1
Salmonella enterica subsp. enterica strain BAA-1586 chromosome, complete genome	1762	1762	100%	0.0	97.21%	CP023468.1
Salmonella enterica subsp. enterica serovar Newport str. USDA-ARS-USMARC-1925 chromosome, complete genome	1762	1762	100%	0.0	97.21%	CP025232.1
Salmonella enterica subsp. salamae serovar 56:z10:e,n,x strain SA20011914 chromosome, complete genome	1762	1762	100%	0.0	97.21%	CP029992.1
Salmonella enterica subsp. enterica strain NCTC9787 genome assembly, chromosome: 1	1762	1762	100%	0.0	97.21%	LS483457.1
Salmonella enterica subsp. salamae strain NCTC9930 genome assembly, chromosome: 1	1762	1762	100%	0.0	97.21%	LS483456.1
Salmonella enterica subsp. enterica strain NCTC9872 genome assembly, chromosome: 1	1762	1762	100%	0.0	97.21%	LS483455.1
Salmonella enterica subsp. enterica serovar Newport str. CDC 2010K-2159, complete genome	1762	1762	100%	0.0	97.21%	CP007559.2
Salmonella enterica subsp. enterica serovar Johannesburg str. ST203, complete genome	1762	1762	100%	0.0	97.21%	CP019411.1
Salmonella enterica subsp. enterica serovar Weltevreden str. 1655 chromosome, complete genome	1762	1762	100%	0.0	97.21%	CP014996.1
Salmonella enterica subsp. enterica serovar Weltevreden genome assembly 99_3134, chromosome : 1	1762	1762	100%	0.0	97.21%	LN890524.1
Salmonella enterica subsp. enterica serovar Weltevreden genome assembly 98_11262, chromosome : 1	1762	1762	100%	0.0	97.21%	LN890522.1
Salmonella enterica subsp. enterica serovar Weltevreden genome assembly C2346, chromosome : 1	1762	1762	100%	0.0	97.21%	LN890520.1
Salmonella enterica subsp. enterica serovar Weltevreden genome assembly 10259, chromosome : 1	1762	1762	100%	0.0	97.21%	LN890518.1
Salmonella enterica subsp. enterica serovar Sloterdijk str. ATCC 15791, complete genome	1762	1762	100%	0.0	97.21%	CP012349.1
Salmonella enterica strain PNUSAS036471 chromosome, complete genome	1762	1762	100%	0.0	97.21%	CP104677.1
Salmonella enterica subsp. enterica serovar Weltevreden strain R17.4942 chromosome, complete genome	1762	1762	100%	0.0	97.21%	CP100718.1
Salmonella enterica subsp. enterica serovar Weltevreden strain R18.0830 chromosome, complete genome	1762	1762	100%	0.0	97.21%	CP100695.1
Salmonella enterica subsp. enterica serovar Newport strain R18.0287 chromosome, complete genome	1762	1762	100%	0.0	97.21%	CP100689.1

Description	Max Score	Total Score	Query Cover	E value	Per. Ident	Accession
Salmonella enterica subsp. enterica serovar Newport strain R18.0234 chromosome, complete genome	1762	1762	100%	0.0	97.21%	CP100744.1
Salmonella enterica strain FDA1033927-S001-001 chromosome, complete genome	1762	1762	100%	0.0	97.22%	CP085053.1
Salmonella enterica subsp. enterica serovar Abaetetuba str. ATCC 35640, complete genome	1762	1762	100%	0.0	97.21%	CP007532.1
Salmonella enterica subsp. enterica serovar Johannesburg strain FSIS1608038 chromosome, complete genome	1762	1762	100%	0.0	97.21%	CP082447.1
Salmonella enterica subsp. enterica serovar Weltevreden strain Colony70 chromosome	1762	1762	100%	0.0	97.21%	CP078529.1
Salmonella enterica subsp. enterica serovar Weltevreden strain Colony65 chromosome	1762	1762	100%	0.0	97.21%	CP078530.1
Salmonella enterica subsp. enterica serovar Derby strain FDA161736 chromosome, complete genome	1762	1762	100%	0.0	97.21%	CP075036.1
Salmonella enterica subsp. enterica strain CFSAN001999 chromosome, complete genome	1762	1762	100%	0.0	97.22%	CP074675.1
Salmonella enterica subsp. enterica serovar Weltevreden strain CFSAN028546 chromosome, complete genome	1762	1762	100%	0.0	97.21%	CP074657.1
Salmonella enterica subsp. salamae strain CFSAN001010 chromosome, complete genome	1762	1762	100%	0.0	97.21%	CP074601.1
Salmonella enterica subsp. enterica serovar Johannesburg strain CFSAN024562 chromosome, complete genome	1762	1762	100%	0.0	97.21%	CP074325.1
Salmonella enterica subsp. enterica serovar Weltevreden strain CFSAN024549 chromosome, complete genome	1762	1762	100%	0.0	97.21%	CP074328.1
Salmonella enterica subsp. enterica serovar Newport str. WA_14882 chromosome, complete genome	1762	1762	100%	0.0	97.21%	CP074207.1
Salmonella enterica subsp. enterica serovar Weltevreden strain Colony63 chromosome	1762	1762	100%	0.0	97.21%	CP070308.1
Salmonella enterica subsp. enterica serovar Weltevreden strain colony_72 chromosome	1762	1762	100%	0.0	97.21%	CP067986.1
Salmonella enterica subsp. enterica serovar Weltevreden strain colony_74 chromosome	1762	1762	100%	0.0	97.21%	CP067985.1
Salmonella enterica subsp. enterica serovar Weltevreden strain colony_64 chromosome	1762	1762	100%	0.0	97.21%	CP067990.1
Salmonella enterica subsp. enterica serovar Weltevreden strain colony_66 chromosome	1762	1762	100%	0.0	97.21%	CP067989.1
Salmonella enterica subsp. enterica serovar Weltevreden strain colony_68 chromosome	1762	1762	100%	0.0	97.21%	CP067988.1
Salmonella enterica subsp. enterica serovar Weltevreden strain colony_69 chromosome	1762	1762	100%	0.0	97.21%	CP067987.1
Salmonella enterica subsp. enterica serovar Weltevreden strain colony_78 chromosome	1762	1762	100%	0.0	97.21%	CP067981.1
Salmonella enterica subsp. enterica serovar Weltevreden strain colony_77 chromosome	1762	1762	100%	0.0	97.21%	CP067982.1

Description	Max Score	Total Score	Query Cover	E value	Per. Ident	Accession
Salmonella enterica subsp. enterica serovar Weltevreden strain colony_75 chromosome	1762	1762	100%	0.0	97.21%	CP067984.1
Salmonella enterica subsp. enterica serovar Weltevreden strain colony_80 chromosome	1762	1762	100%	0.0	97.21%	CP067980.1
Salmonella enterica subsp. enterica serovar Weltevreden strain colony_76 chromosome	1762	1762	100%	0.0	97.21%	CP067983.1
Salmonella enterica subsp. enterica serovar Typhimurium strain NCTC 74 chromosome, complete genome	1762	1762	100%	0.0	97.22%	CP064709.1
Salmonella enterica subsp. enterica serovar Weltevreden str. 2007-60-3289-1 complete genome, contig 53	1762	1762	100%	0.0	97.21%	FR775240.1
Salmonella enterica subsp. enterica serovar Typhi strain BRD948 genome assembly, chromosome: 1	1760	1760	100%	0.0	97.21%	OW706648.1
Salmonella enterica subsp. enterica serovar Kottbus strain Kharkiv chromosome, complete genome	1760	1760	100%	0.0	97.21%	CP062220.1
Salmonella enterica subsp. enterica serovar Adjame strain 389598 chromosome, complete genome	1757	1757	100%	0.0	97.12%	CP054827.1
Salmonella enterica strain 2011K-1440 chromosome, complete genome	1757	1757	100%	0.0	97.12%	CP053585.1
Salmonella enterica subsp. enterica serovar Albany strain CVM N18S2238 chromosome, complete genome	1757	1757	100%	0.0	97.12%	CP049312.1
Salmonella enterica strain FDAARGOS_709 chromosome	1757	1757	100%	0.0	97.12%	CP050990.1
Salmonella enterica subsp. enterica serovar Adjame strain 381330 chromosome, complete genome	1757	1757	100%	0.0	97.12%	CP049880.1
Salmonella enterica subsp. enterica serovar Adjame strain 387507 chromosome, complete genome	1757	1757	100%	0.0	97.12%	CP049883.1
Salmonella enterica subsp. enterica serovar Adjame strain 434304 chromosome, complete genome	1757	1757	100%	0.0	97.12%	CP049882.1
Salmonella enterica subsp. enterica serovar Adjame strain 416020 chromosome, complete genome	1757	1757	100%	0.0	97.12%	CP049884.1
Salmonella enterica subsp. enterica serovar Adjame strain 388789 chromosome, complete genome	1757	1757	100%	0.0	97.12%	CP049875.1
Salmonella enterica subsp. enterica serovar Adjame strain 400321 chromosome, complete genome	1757	1757	100%	0.0	97.12%	CP049877.1
Salmonella enterica subsp. enterica serovar Adjame strain 435414 chromosome, complete genome	1757	1757	100%	0.0	97.12%	CP049881.1
Salmonella enterica subsp. enterica serovar Adjame strain 409960 chromosome, complete genome	1757	1757	100%	0.0	97.12%	CP049879.1
Salmonella enterica strain FDAARGOS_710 chromosome, complete genome	1757	1757	100%	0.0	97.12%	CP046277.1
Salmonella enterica subsp. enterica serovar Enteritidis strain AUSMDU00010528 chromosome, complete genome	1757	1757	100%	0.0	97.12%	CP045955.1
Salmonella enterica subsp. enterica serovar Newport strain SAP18-8729 chromosome, complete genome	1757	1757	100%	0.0	97.12%	CP041208.1
Salmonella enterica subsp. enterica serovar Newport str. USDA-ARS-USMARC-1923 chromosome, complete genome	1757	1757	100%	0.0	97.12%	CP025273.1

Description	Max Score	Total Score	Query Cover	E value	Per. Ident	Accession
Salmonella enterica subsp. enterica serovar Albany strain sg_wt5 chromosome, complete genome	1757	1757	100%	0.0	97.12%	CP036165.1
Salmonella enterica subsp. enterica serovar Newport str. CDC 2012K-0663 chromosome, complete genome	1757	1757	100%	0.0	97.12%	CP025243.1
Salmonella enterica subsp. enterica serovar Newport str. USDA-ARS-USMARC-1929 chromosome, complete genome	1757	1757	100%	0.0	97.12%	CP025241.1
Salmonella enterica subsp. enterica serovar Newport str. USDA-ARS-USMARC-1928 chromosome, complete genome	1757	1757	100%	0.0	97.12%	CP025237.1
Salmonella enterica subsp. enterica serovar Newport str. USDA-ARS-USMARC-1926 chromosome, complete genome	1757	1757	100%	0.0	97.12%	CP025234.1
Salmonella enterica subsp. enterica serovar Newport str. USDA-ARS-USMARC-1924 chromosome, complete genome	1757	1757	100%	0.0	97.12%	CP025230.1
Salmonella enterica strain SA20075157 chromosome, complete genome	1757	1757	100%	0.0	97.12%	CP030217.1
Salmonella enterica subsp. enterica serovar Give strain NCTC5778 genome assembly, chromosome: 1	1757	1757	100%	0.0	97.12%	LS483463.1
Salmonella enterica subsp. enterica serovar Bergen str. ST350, complete genome	1757	1757	100%	0.0	97.12%	CP019405.1
Salmonella enterica subsp. enterica serovar Antsalova str. S01-0511, complete genome	1757	1757	100%	0.0	97.12%	CP019116.1
Salmonella enterica subsp. enterica serovar Chester str. ATCC 11997 chromosome, complete genome	1757	1757	100%	0.0	97.12%	CP019178.1
Salmonella enterica subsp. enterica serovar Albany str. ATCC 51960 chromosome, complete genome	1757	1757	100%	0.0	97.12%	CP019177.1
Salmonella enterica subsp. enterica serovar Newport strain 0007-33 chromosome, complete genome	1757	1757	100%	0.0	97.12%	CP013685.1
Salmonella enterica subsp. enterica serovar Newport strain CFSAN003890, complete genome	1757	1757	100%	0.0	97.12%	CP016012.1
Salmonella enterica subsp. enterica serovar Newport str. WA_14882 chromosome, complete genome	1757	1757	100%	0.0	97.12%	CP016357.1
Salmonella enterica subsp. enterica serovar Newport strain 0307-213, complete genome	1757	1757	100%	0.0	97.12%	CP012599.1
Salmonella enterica subsp. enterica serovar Newport strain 0211-109, complete genome	1757	1757	100%	0.0	97.12%	CP012598.1
Salmonella enterica subsp. enterica serovar Newport strain 0112-791, complete genome	1757	1757	100%	0.0	97.12%	CP012597.1
Salmonella enterica strain 2012K-1379 chromosome 1, complete sequence	1757	1757	100%	0.0	97.12%	CP111088.1
Salmonella enterica subsp. enterica serovar Newport str. CVM 21550, complete genome	1757	1757	100%	0.0	97.12%	CP010283.1
Salmonella enterica subsp. enterica serovar Newport str. CVM 21538, complete genome	1757	1757	100%	0.0	97.12%	CP010282.1
Salmonella enterica subsp. enterica serovar Newport str. CVM 22513, complete genome	1757	1757	100%	0.0	97.12%	CP010281.1
Salmonella enterica subsp. enterica serovar Newport str. CVM 22425, complete genome	1757	1757	100%	0.0	97.12%	CP010279.1

Description	Max Score	Total Score	Query Cover	E value	Per. Ident	Accession
Salmonella enterica subsp. enterica serovar Newport str. CVM N1543, complete genome	1757	1757	100%	0.0	97.12%	CP010284.1
Salmonella enterica strain PNUSAS118466 chromosome, complete genome	1757	1757	100%	0.0	97.12%	CP104638.1
Salmonella enterica subsp. enterica serovar Newport str. CVM 22462, complete genome	1757	1757	100%	0.0	97.12%	CP010280.1
Salmonella enterica subsp. enterica serovar Newport str. CVM N18486, complete genome	1757	1757	100%	0.0	97.12%	CP009561.1
Salmonella enterica strain PNUSAS020177 chromosome, complete genome	1757	1757	100%	0.0	97.12%	CP094293.1
Salmonella enterica subsp. enterica serovar Reading strain PNUSAS003019 chromosome, complete genome	1757	1757	100%	0.0	97.12%	CP093134.1
Salmonella enterica subsp. enterica serovar Reading strain PNUSAS010176 chromosome, complete genome	1757	1757	100%	0.0	97.12%	CP093132.1
Salmonella enterica subsp. enterica serovar Reading strain PNUSAS039653 chromosome, complete genome	1757	1757	100%	0.0	97.12%	CP093129.1
Salmonella enterica strain PNUSAS060563 chromosome, complete genome	1757	1757	100%	0.0	97.12%	CP093147.1
Salmonella enterica subsp. enterica serovar Newport strain CVM N16S224 chromosome, complete genome	1757	1757	100%	0.0	97.12%	CP082641.1
Salmonella sp. A7 chromosome, complete genome	1757	1757	100%	0.0	97.12%	CP084001.1
Salmonella enterica subsp. enterica serovar Newport strain CVM N18S1655 chromosome, complete genome	1757	1757	100%	0.0	97.12%	CP082547.1
Salmonella enterica subsp. enterica serovar Albany strain CVM N18S1350 chromosome, complete genome	1757	1757	100%	0.0	97.12%	CP082666.1
Salmonella enterica subsp. enterica serovar Reading strain CVM N18S0035 chromosome, complete genome	1757	1757	100%	0.0	97.12%	CP082611.1
Salmonella enterica subsp. enterica serovar Newport strain CVM N17S1196 chromosome, complete genome	1757	1757	100%	0.0	97.12%	CP082598.1
Salmonella enterica subsp. enterica serovar Reading strain CVM N17S546 chromosome, complete genome	1757	1757	100%	0.0	97.12%	CP082701.1
Salmonella enterica subsp. enterica serovar Reading strain FSIS1701482 chromosome, complete genome	1757	1757	100%	0.0	97.12%	CP082386.1
Salmonella enterica subsp. enterica serovar Reading strain CVM N16S084 chromosome, complete genome	1757	1757	100%	0.0	97.12%	CP082733.1
Salmonella enterica subsp. enterica serovar Newport strain FSIS1608364 chromosome, complete genome	1757	1757	100%	0.0	97.12%	CP082439.1
Salmonella enterica subsp. enterica serovar Newport strain FSIS1607290 chromosome, complete genome	1757	1757	100%	0.0	97.12%	CP082478.1
Salmonella enterica subsp. enterica serovar Reading strain CVM N17S288 isolate 17NY04GT13-S3 chromosome, complete genome	1757	1757	100%	0.0	97.12%	CP082721.1
Salmonella enterica subsp. enterica serovar Reading strain CVM N18S1429 isolate 18MN08GT11-3 chromosome, complete genome	1757	1757	100%	0.0	97.12%	CP082566.1

Description	Max Score	Total Score	Query Cover	E value	Per. Ident	Accession
Salmonella enterica subsp. enterica serovar Newport str. USMARC-S3124.1, complete genome	1757	1757	100%	0.0	97.12%	CP006631.1
Salmonella enterica subsp. enterica serovar Sandiego strain CFSAN012509 chromosome, complete genome	1757	1757	100%	0.0	97.12%	CP075039.1
Salmonella enterica subsp. enterica serovar Newport strain CFSAN022633 chromosome, complete genome	1757	1757	100%	0.0	97.12%	CP075022.1
Salmonella enterica subsp. enterica serovar Sandiego strain CFSAN012498 chromosome, complete genome	1757	1757	100%	0.0	97.12%	CP075040.1
Salmonella enterica subsp. enterica serovar Give strain CFSAN012622 chromosome, complete genome	1757	1757	100%	0.0	97.12%	CP075037.1
Salmonella enterica subsp. enterica serovar Newport strain CFSAN024515 chromosome, complete genome	1757	1757	100%	0.0	97.12%	CP074333.1
Salmonella enterica subsp. enterica serovar Newport strain CFSAN024541 chromosome, complete genome	1757	1757	100%	0.0	97.12%	CP074330.1
Salmonella enterica subsp. enterica serovar Newport strain CFSAN024414 chromosome, complete genome	1757	1757	100%	0.0	97.12%	CP074337.1
Salmonella enterica subsp. enterica serovar Newport strain CFSAN024555 chromosome, complete genome	1757	1757	100%	0.0	97.12%	CP074327.1
Salmonella enterica subsp. enterica strain CFSAN004111 chromosome, complete genome	1757	1757	100%	0.0	97.12%	CP074308.1
Salmonella enterica subsp. enterica serovar Sandiego strain CFSAN024590 chromosome, complete genome	1757	1757	100%	0.0	97.12%	CP074265.1
Salmonella enterica subsp. enterica serovar Newport strain CFSAN024608 chromosome, complete genome	1757	1757	100%	0.0	97.12%	CP074263.1
Salmonella enterica subsp. enterica serovar Newport strain CFSAN024599 chromosome, complete genome	1757	1757	100%	0.0	97.12%	CP074264.1
Salmonella enterica subsp. enterica serovar Newport str. CFSAN000598 strain SGSC 2494 isolate SARB37 chromosome, complete genome	1757	1757	100%	0.0	97.12%	CP074217.1
Salmonella enterica subsp. enterica serovar Abaetetuba str. ATCC 35640 chromosome, complete genome	1757	1757	100%	0.0	97.12%	CP074211.1
Salmonella enterica subsp. enterica serovar Albany strain R16.0556 chromosome, complete genome	1757	1757	100%	0.0	97.12%	CP061929.2
Salmonella enterica subsp. enterica serovar Albany strain R16.0431 chromosome, complete genome	1757	1757	100%	0.0	97.12%	CP062004.2
Salmonella enterica subsp. enterica serovar Albany strain R17.5974 chromosome, complete genome	1757	1757	100%	0.0	97.12%	CP060730.2
Salmonella enterica subsp. enterica serovar Albany strain CFSAN103854 chromosome, complete genome	1757	1757	100%	0.0	97.12%	CP066333.1
Salmonella enterica subsp. enterica serovar Newport strain CVM 24374 chromosome, complete genome	1757	1757	100%	0.0	97.12%	CP051372.1
Salmonella enterica subsp. enterica serovar Newport strain CVM 34530 chromosome, complete genome	1757	1757	100%	0.0	97.12%	CP051317.1
Salmonella enterica subsp. enterica serovar Albany strain R16.1424 chromosome, complete genome	1757	1757	100%	0.0	97.12%	CP065572.1

Description	Max Score	Total Score	Query Cover	E value	Per. Ident	Accession
Salmonella enterica subsp. enterica serovar Albany strain R13.1544 chromosome, complete genome	1757	1757	100%	0.0	97.12%	CP065566.1
Salmonella enterica subsp. enterica serovar Albany strain R15.2267 chromosome, complete genome	1757	1757	100%	0.0	97.12%	CP065564.1
Salmonella enterica subsp. enterica serovar Albany strain R17.4301 chromosome, complete genome	1757	1757	100%	0.0	97.12%	CP062795.1
Salmonella enterica subsp. enterica serovar Albany strain R17.2117 chromosome, complete genome	1757	1757	100%	0.0	97.12%	CP063330.1
Salmonella enterica subsp. enterica serovar Newport str. SL254, complete genome	1757	1757	100%	0.0	97.12%	CP001113.1
Salmonella enterica subsp. enterica serovar Adjame strain 353868 chromosome, complete genome	1751	1751	100%	0.0	97.02%	CP049878.1
Salmonella enterica subsp. enterica serovar Adjame strain 374589 chromosome, complete genome	1751	1751	100%	0.0	97.02%	CP049874.1
Salmonella enterica subsp. enterica serovar Daytona strain NCTC7102 genome assembly, chromosome: 1	1751	1751	100%	0.0	97.02%	LR133909.1
Salmonella enterica subsp. enterica serovar India str. SA20085604, complete genome	1751	1751	100%	0.0	97.02%	CP022015.1
Salmonella enterica subsp. enterica serovar Koessen str. S-1501, complete genome	1751	1751	100%	0.0	97.02%	CP019412.1
Salmonella enterica subsp. enterica serovar Panama str. ATCC 7378, complete genome	1751	1751	100%	0.0	97.02%	CP012346.1
Salmonella sp. 2018103 chromosome, complete genome	1751	1751	100%	0.0	97.02%	CP111029.1
Salmonella enterica subsp. enterica serovar Shamba strain CFSAN029516 chromosome, complete genome	1751	1751	100%	0.0	97.02%	CP074648.1
Salmonella enterica strain Colony626 chromosome	1751	1751	100%	0.0	97.02%	CP070317.1
Salmonella enterica subsp. enterica serovar Adjame strain 367320 chromosome, complete genome	1746	1746	100%	0.0	96.93%	CP049873.1
Salmonella enterica subsp. enterica serovar Stanleyville strain RSE01 chromosome, complete genome	1746	1746	100%	0.0	96.93%	CP034723.1
Salmonella enterica subsp. enterica serovar Stanleyville strain RSE30 chromosome, complete genome	1746	1746	100%	0.0	96.93%	CP034703.1
Salmonella enterica subsp. enterica serovar Stanleyville strain RSE39 chromosome, complete genome	1746	1746	100%	0.0	96.93%	CP034700.1
Salmonella enterica subsp. enterica serovar Panama str. ATCC 7378 chromosome, complete genome	1746	1746	100%	0.0	96.93%	CP074210.1
Salmonella enterica subsp. enterica serovar Newport str. CVM 21554, complete genome	1744	1744	100%	0.0	96.93%	CP009565.1
Salmonella enterica subsp. enterica serovar Brandenburg strain USDA-ARS-USMARC-60983 chromosome, complete genome	1740	1740	100%	0.0	96.83%	CP025280.1
Salmonella enterica subsp. enterica strain NCTC8272 genome assembly, chromosome: 1	1740	1740	100%	0.0	96.84%	LR134149.1
Salmonella enterica subsp. enterica serovar Brandenburg strain SA20064858 chromosome, complete genome	1740	1740	100%	0.0	96.83%	CP030002.1

Description	Max Score	Total Score	Query Cover	E value	Per. Ident	Accession
Salmonella enterica subsp. enterica serovar Brandenburg strain SA20113174 chromosome, complete genome	1740	1740	100%	0.0	96.83%	CP029999.1
Salmonella enterica subsp. enterica serovar Brandenburg strain FSIS1607501 chromosome, complete genome	1740	1740	100%	0.0	96.83%	CP082464.1
Salmonella enterica subsp. enterica serovar Brandenburg strain CFSAN001008 chromosome, complete genome	1740	1740	100%	0.0	96.83%	CP074614.1
Salmonella enterica subsp. enterica serovar Brandenburg strain CFSAN024765 chromosome, complete genome	1740	1740	100%	0.0	96.83%	CP074258.1
Salmonella enterica subsp. enterica serovar Brandenburg strain CFSAN027396 chromosome, complete genome	1740	1740	100%	0.0	96.83%	CP074245.1
Salmonella sp. WGH-01 chromosome, complete genome	1696	1696	100%	0.0	96.16%	CP096171.1
Salmonella enterica subsp. enterica serovar Kentucky strain NTS-1328 chromosome	1696	1696	100%	0.0	96.25%	CP069813.1
Salmonella enterica strain 94-0093 chromosome, complete genome	1690	1690	100%	0.0	95.97%	CP053581.1
Salmonella enterica subsp. enterica serovar Infantis strain NTS-573 chromosome	1688	1688	100%	0.0	96.06%	CP069814.1
Salmonella enterica strain 08-0466 chromosome, complete genome	1663	1663	100%	0.0	95.49%	CP053411.1
Salmonella enterica subsp. salamae serovar 60:z10:z39 strain 2011K-1889 chromosome, complete genome	1663	1663	100%	0.0	95.49%	CP053330.1
Salmonella enterica subsp. diarizonae serovar 50:k:z str. MZ0080, complete genome	1652	1652	100%	0.0	95.29%	CP022142.1
Salmonella enterica subsp. arizonae strain S499 chromosome, complete genome	1652	1652	100%	0.0	95.29%	CP082954.1
Salmonella enterica subsp. diarizonae strain CFSAN030538 chromosome, complete genome	1652	1652	100%	0.0	95.29%	CP075144.1
Salmonella enterica subsp. diarizonae serovar b,50:-:- strain XXB1403 chromosome, complete genome	1652	1652	100%	0.0	95.29%	CP059886.1
Salmonella enterica subsp. diarizonae serovar 61:k:1,5,(7) strain 14-SA00836-0 chromosome, complete genome	1646	1646	100%	0.0	95.20%	CP054422.1
Salmonella enterica strain 72-4606 chromosome, complete genome	1646	1646	100%	0.0	95.20%	CP053413.1
Salmonella enterica subsp. diarizonae serovar 47:k:z35 strain 2015K-1072 chromosome, complete genome	1646	1646	100%	0.0	95.20%	CP053583.1
Salmonella enterica subsp. diarizonae serovar 47:k:z35 strain 2009K1094 chromosome, complete genome	1646	1646	100%	0.0	95.20%	CP053335.1
Salmonella enterica subsp. diarizonae serovar 65:z10:e,n,x,z15 strain 2015K-0019 chromosome, complete genome	1646	1646	100%	0.0	95.20%	CP053323.1
Salmonella enterica subsp. diarizonae serovar 60:r:z strain 2016K-0056 chromosome, complete genome	1646	1646	100%	0.0	95.20%	CP053319.1
Salmonella enterica subsp. arizonae strain NCTC10047 genome assembly, chromosome: 1	1646	1646	100%	0.0	95.20%	LR134156.1
Salmonella enterica subsp. diarizonae serovar 61:k:1,5,(7) strain 16-SA00356 chromosome, complete genome	1646	1646	100%	0.0	95.20%	CP034074.1
Salmonella enterica strain SA20021456 chromosome, complete genome	1646	1646	100%	0.0	95.20%	CP030219.1

Description	Max Score	Total Score	Query Cover	E value	Per. Ident	Accession
Salmonella enterica strain SA20100201 chromosome, complete genome	1646	1646	100%	0.0	95.20%	CP030180.1
Salmonella enterica subsp. diarizonae serovar 59:z10:- strain SA20051472 chromosome	1646	1646	100%	0.0	95.20%	CP030026.1
Salmonella enterica subsp. diarizonae strain NCTC10381 genome assembly, chromosome: 1	1646	1646	100%	0.0	95.20%	LS483474.1
Salmonella enterica strain NCTC10436 genome assembly, chromosome: 1	1646	1646	100%	0.0	95.20%	LS483428.1
Salmonella enterica subsp. diarizonae strain HZS154 chromosome, complete genome	1646	1646	100%	0.0	95.20%	CP023345.1
Salmonella enterica subsp. diarizonae serovar 65:c:z str. SA20044251, complete genome	1646	1646	100%	0.0	95.20%	CP022135.1
Salmonella enterica subsp. diarizonae strain 11-01853 chromosome, complete genome	1646	1646	100%	0.0	95.20%	CP011289.1
Salmonella enterica subsp. diarizonae strain 11-01855 chromosome, complete genome	1646	1646	100%	0.0	95.20%	CP011288.1
Salmonella enterica subsp. enterica strain CFSAN058540 chromosome, complete genome	1646	1646	100%	0.0	95.20%	CP077710.1
Salmonella enterica strain CFSAN044865 chromosome, complete genome	1646	1646	100%	0.0	95.20%	CP075140.1
Salmonella enterica subsp. diarizonae serovar 60:r:e,n,x,z15 strain CFSAN044923 chromosome, complete genome	1646	1646	100%	0.0	95.20%	CP075128.1
Salmonella enterica subsp. salamae serovar 48:z81:z39 strain 2015K-0023 chromosome, complete genome	1644	1644	100%	0.0	95.20%	CP053322.1
Salmonella enterica strain 68-4603 chromosome, complete genome	1640	1640	100%	0.0	95.10%	CP053406.1
Salmonella enterica strain 87-0091 chromosome, complete genome	1640	1640	100%	0.0	95.10%	CP053408.1
Salmonella enterica subsp. diarizonae serovar 48:i:z strain SA20121591 chromosome, complete genome	1640	1640	100%	0.0	95.10%	CP029989.1
Salmonella enterica subsp. enterica serovar Macclesfield str. S-1643, complete genome	1640	1640	100%	0.0	95.10%	CP022117.1
Salmonella enterica subsp. diarizonae strain LHICA_D1 chromosome, complete genome	1640	1640	100%	0.0	95.10%	CP078142.1
Salmonella enterica subsp. salamae serovar 55:k:z39 str. 1315K, complete genome	1635	1635	100%	0.0	95.00%	CP022139.1
Salmonella enterica subsp. enterica serovar Pullorum strain QJ-2D-Sal chromosome, complete genome	1631	1631	87%	0.0	98.70%	CP022963.1
Salmonella enterica subsp. enterica serovar Gallinarum/Pullorum strain R51 chromosome, complete genome	1631	1631	87%	0.0	98.70%	CP068386.1
Salmonella enterica subsp. enterica serovar Pullorum genome assembly S44987_1, chromosome : I	1631	1631	87%	0.0	98.70%	LK931482.1
Salmonella enterica subsp. enterica serovar Pullorum str. S06004, complete genome	1631	1631	87%	0.0	98.70%	CP006575.1
Salmonella enterica subsp. enterica serovar Pullorum strain CFSAN022627 chromosome, complete genome	1631	1631	87%	0.0	98.70%	CP075028.1
Salmonella enterica subsp. enterica serovar Pullorum str. CFSAN000606 strain SGSC 2508 isolate SARB51 chromosome, complete genome	1631	1631	87%	0.0	98.70%	CP074215.1

Description	Max Score	Total Score	Query Cover	E value	Per. Ident	Accession
Salmonella enterica subsp. salamae serovar 6,8:a:z52 strain 62-3163 chromosome, complete genome	1629	1629	100%	0.0	94.91%	CP053318.1
Salmonella enterica subsp. salamae serovar 40:c:e,n,z15 strain 2013K-0524 chromosome, complete genome	1628	1628	100%	0.0	94.91%	CP053325.1
Salmonella bongori serovar 40:z35:- strain CFSAN001045 chromosome, complete genome	1607	1607	100%	0.0	94.52%	CP074592.1
Salmonella enterica subsp. enterica serovar Gallinarum/pullorum str. RKS5078, complete genome	1596	1596	87%	0.0	97.86%	CP003047.1
Salmonella enterica subsp. enterica serovar Pullorum str. ATCC 9120, complete genome	1589	1589	87%	0.0	97.75%	CP012347.1
Salmonella enterica subsp. enterica serovar Gallinarum/Pullorum str. CDC1983-67 chromosome, complete genome	1589	1589	87%	0.0	97.75%	CP003786.1
Salmonella enterica subsp. enterica serovar Pullorum strain CFSAN022642 chromosome, complete genome	1589	1589	87%	0.0	97.75%	CP075018.1
Salmonella enterica strain 2014K-0203 chromosome, complete genome	1496	1496	100%	0.0	92.60%	CP053409.1
Salmonella enterica subsp. arizonae serovar 53:-:- str. SA20100345 chromosome, complete genome	1496	1496	100%	0.0	92.60%	CP022504.1
Salmonella enterica subsp. arizonae serovar 62:z36:- str. RKS2983, complete genome	1496	1496	100%	0.0	92.60%	CP006693.1
Salmonella enterica subsp. arizonae strain LHICA_AZ23 chromosome, complete genome	1496	1496	100%	0.0	92.60%	CP079713.1
Salmonella enterica subsp. enterica strain Se13 chromosome, complete genome	1496	1496	100%	0.0	92.60%	CP067337.1
Salmonella enterica subsp. arizonae serovar 13:g,z51:- strain 2009K0967 chromosome, complete genome	1485	1485	100%	0.0	92.41%	CP053401.1
Salmonella enterica strain 2014K-1020 chromosome, complete genome	1485	1485	100%	0.0	92.41%	CP053584.1
Salmonella enterica subsp. arizonae serovar 41:z4,z23:- strain 2016K-0011 chromosome, complete genome	1485	1485	100%	0.0	92.41%	CP053321.1
Salmonella enterica subsp. arizonae serovar 63:g,z51:- strain SA19981204 chromosome, complete genome	1485	1485	100%	0.0	92.41%	CP029991.1
Salmonella enterica subsp. arizonae serovar 41:z4,z23:- str. 01-0089 chromosome, complete genome	1485	1485	100%	0.0	92.41%	CP074593.1
Salmonella enterica subsp. arizonae serovar 62:z4,z23:- strain RSK2980 chromosome, complete genome	1485	1485	100%	0.0	92.41%	CP000880.1
Salmonella enterica subsp. arizonae strain NCTC7307 genome assembly, chromosome: 1	1483	1483	100%	0.0	92.41%	LS483466.1
Salmonella enterica strain FDAARGOS_765 chromosome, complete genome	1480	1480	100%	0.0	92.32%	CP041011.1
Salmonella enterica subsp. arizonae strain PartD-Sarizonae-RM8376 chromosome, complete genome	1480	1480	100%	0.0	92.32%	CP064363.1
Salmonella enterica subsp. arizonae strain NCTC7306 genome assembly, chromosome: 1	1478	1478	100%	0.0	92.32%	LR134150.1
Salmonella enterica subsp. arizonae strain NCTC7300 genome assembly, chromosome: 1	1472	1472	100%	0.0	92.22%	LR133910.1
Salmonella bongori strain 92-0238 chromosome, complete genome	1397	1397	100%	0.0	90.88%	CP053417.1

Description	Max Score	Total Score	Query Cover	E value	Per. Ident	Accession
Salmonella enterica subsp. VII serovar 1,40:g,z51:-- strain 2439-64 chromosome, complete genome	1391	1391	100%	0.0	90.78%	CP053582.1
Salmonella enterica subsp. VII str. CFSAN000554 strain SGSC 3121 isolate SARC16 chromosome, complete genome	1391	1391	100%	0.0	90.78%	CP074220.1
Salmonella enterica subsp. houtenae serovar 44:z4,z32:- strain 2009K1701 chromosome, complete genome	1363	1363	100%	0.0	90.30%	CP053332.1
Salmonella enterica subsp. houtenae str. CFSAN000552 isolate SARC10 chromosome, complete genome	1363	1363	100%	0.0	90.30%	CP045761.1
Salmonella enterica strain SA20055162 chromosome, complete genome	1363	1363	100%	0.0	90.30%	CP030238.1
Salmonella enterica strain 2012K-0845 chromosome, complete genome	1358	1358	100%	0.0	90.20%	CP053579.1
Salmonella enterica strain SA20080453 chromosome, complete genome	1358	1358	100%	0.0	90.20%	CP030194.1
Salmonella enterica subsp. houtenae serovar Houten strain NCTC10401 genome assembly, chromosome: 1	1358	1358	100%	0.0	90.20%	LS483478.1
Salmonella enterica subsp. houtenae serovar 45:g,z51:- strain 20-369 chromosome, complete genome	1358	1358	100%	0.0	90.20%	CP075174.1
Salmonella enterica subsp. enterica strain C68 chromosome, complete genome	1358	1358	99%	0.0	90.45%	CP073333.1
Salmonella enterica subsp. houtenae serovar 43:z4 strain CVM 24399 chromosome, complete genome	1358	1358	100%	0.0	90.20%	CP051368.1
Salmonella enterica strain 2011K-0868 chromosome, complete genome	1352	1352	100%	0.0	90.11%	CP053414.1
Salmonella enterica subsp. houtenae strain NCTC7318 genome assembly, chromosome: 1	1352	1352	100%	0.0	90.11%	LR134159.1
Salmonella enterica strain CFSAN057252 chromosome, complete genome	1352	1352	100%	0.0	90.11%	CP076088.1
Salmonella enterica strain CFSAN057226 chromosome, complete genome	1352	1352	100%	0.0	90.11%	CP076093.1
Salmonella bongori strain 85-0051 chromosome, complete genome	1330	1330	100%	0.0	89.72%	CP053416.1
Salmonella bongori strain 04-0440 chromosome, complete genome	1330	1330	100%	0.0	89.72%	CP035676.1
Salmonella enterica subsp. houtenae strain 04-0440 chromosome, complete genome	1330	1330	100%	0.0	89.72%	CP045914.1
Salmonella bongori serovar 48:z41:-- str. RKS3044, complete genome	1323	1323	99%	0.0	89.62%	CP006692.1
Salmonella bongori serovar 48:z81:- strain 08-0158 chromosome, complete genome	1290	1290	99%	0.0	89.04%	CP053336.1
Salmonella bongori N268-08, complete genome	1290	1290	99%	0.0	89.04%	CP006608.1
Salmonella enterica subsp. enterica strain C83 chromosome, complete genome	1275	1275	100%	0.0	89.21%	CP073335.1
Salmonella enterica subsp. enterica strain H89 chromosome, complete genome	1275	1275	100%	0.0	88.96%	CP073321.1
Salmonella bongori CFSAN000510 chromosome, complete genome	1273	1273	99%	0.0	88.75%	CP045766.1
Salmonella bongori serovar 66:z41:- str. SA19983605, complete genome	1273	1273	99%	0.0	88.75%	CP022120.1
Salmonella bongori CFSAN000510 strain SGSC 3100 chromosome, complete genome	1273	1273	99%	0.0	88.75%	CP074233.1
Salmonella bongori strain Se40 chromosome, complete genome	1273	1273	99%	0.0	88.75%	CP067369.1

Description	Max Score	Total Score	Query Cover	E value	Per. Ident	Accession
Salmonella bongori NCTC 12419, culture collection SGSC SARC11, complete genome	1273	1273	99%	0.0	88.75%	FR877557.1
Salmonella enterica subsp. enterica strain P29 chromosome, complete genome	1271	1271	100%	0.0	88.85%	CP073328.1
Salmonella enterica subsp. enterica strain B35 chromosome, complete genome	1249	1249	100%	0.0	88.87%	CP073338.1
Salmonella enterica subsp. enterica strain B50 chromosome, complete genome	1232	1232	99%	0.0	88.69%	CP073323.1
Salmonella bongori strain NCTC12419 genome assembly, chromosome: 1	1210	1210	99%	0.0	87.69%	LR134137.1
Salmonella enterica subsp. enterica strain H36 chromosome, complete genome	1188	1188	97%	0.0	88.41%	CP073331.1
Salmonella enterica subsp. enterica serovar Typhimurium strain YU07-18 chromosome, complete genome	1177	1797	96%	0.0	98.64%	CP035547.1
Salmonella enterica subsp. enterica strain P22 chromosome, complete genome	1147	1147	99%	0.0	87.32%	CP073326.1
Salmonella enterica subsp. enterica strain C110 chromosome, complete genome	1127	1127	100%	0.0	86.60%	CP073336.1
Salmonella enterica subsp. enterica serovar Weltevreden strain H2 chromosome	363	363	35%	5e-95	85.12%	CP073319.1

Alignments

Salmonella enterica subsp. enterica serovar Gallinarum str. 9184 chromosome, complete genome
Sequence ID: **CP019035.1** Length: 4609911 Number of Matches: 1
Range 1: 18652 to 19692

Score	Expect	Identities	Gaps	Strand	Frame
1923 bits(1041)	0.0()	1041/1041(100%)	0/1041(0%)	Plus/Plus	

Features:

```
Query  1      ATGCGCGCGCTTATTTCGTTCttttttttGATAATCATCGTCTCGAATGTAGAAACAGCG  60
              ||||||||||||||||||||||||||||||||||||||||||||||||||||||||||||
Sbjct  18652  ATGCGCGCGCTTATTTCGTTCTTTTTTTTGATAATCATCGTCTCGAATGTAGAAACAGCG  18711
Query  61     TCAGCCGAAACCGGACTGCTACACTGGACGCGCGCCGATCGGGCGATTCCCTGGCGGCAA  120
              ||||||||||||||||||||||||||||||||||||||||||||||||||||||||||||
Sbjct  18712  TCAGCCGAAACCGGACTGCTACACTGGACGCGCGCCGATCGGGCGATTCCCTGGCGGCAA  18771
Query  121    ACGTCAGTCAATGCGAGTAAACCCTGGAAACTGTGCGCATTATACCCCAGCCTGAAAGAT  180
              ||||||||||||||||||||||||||||||||||||||||||||||||||||||||||||
Sbjct  18772  ACGTCAGTCAATGCGAGTAAACCCTGGAAACTGTGCGCATTATACCCCAGCCTGAAAGAT  18831
Query  181    TCTTACTGGTTGTCCGTCAACTACGGGATGCAAAAAGCGGCCAAATTGTATGGCGTTGAT  240
              ||||||||||||||||||||||||||||||||||||||||||||||||||||||||||||
Sbjct  18832  TCTTACTGGTTGTCCGTCAACTACGGGATGCAAAAAGCGGCCAAATTGTATGGCGTTGAT  18891
Query  241    TTAAAAGTGCTGGAAGCGAATGGCTATCGGCAACTGGCGACGCAACAACAGCAAATGACG  300
              ||||||||||||||||||||||||||||||||||||||||||||||||||||||||||||
Sbjct  18892  TTAAAAGTGCTGGAAGCGAATGGCTATCGGCAACTGGCGACGCAACAACAGCAAATGACG  18951
Query  301    CAGTGCCGGGAGTGGGGCGCCGACGCTATTCTGCTTGGCAGCAGTACCATTCGTTTCCCG  360
              ||||||||||||||||||||||||||||||||||||||||||||||||||||||||||||
Sbjct  18952  CAGTGCCGGGAGTGGGGCGCCGACGCTATTCTGCTTGGCAGCAGTACCATTCGTTTCCCG  19011
Query  361    GAACTGGAACGGTATGCCGGTAATGTGCCGGTTATTGAAGTGGCGAACGTGATACGCGAC  420
              ||||||||||||||||||||||||||||||||||||||||||||||||||||||||||||
Sbjct  19012  GAACTGGAACGGTATGCCGGTAATGTGCCGGTTATTGAAGTGGCGAACGTGATACGCGAC  19071
Query  421    GCCAGCGTGGCAACTCACGTCGGTCTGCCGTGGTTTCAGATGGGTTATCTGCCGGGACGT  480
              ||||||||||||||||||||||||||||||||||||||||||||||||||||||||||||
Sbjct  19072  GCCAGCGTGGCAACTCACGTCGGTCTGCCGTGGTTTCAGATGGGTTATCTGCCGGGACGT  19131
Query  481    TTCCTGGTGCAGTGGAGCAAAGGAAAAACGCTTAACGTTTTACTCTTCCCGGGGCCGGAG  540
              ||||||||||||||||||||||||||||||||||||||||||||||||||||||||||||
Sbjct  19132  TTCCTGGTGCAGTGGAGCAAAGGAAAAACGCTTAACGTTTTACTCTTCCCGGGGCCGGAG  19191
Query  541    GAAGCTGGCGGCAGTCAGGAGATGGTGGAGGGTTTTCGTCAGGCAATTAAAGGCAGCGCC  600
              ||||||||||||||||||||||||||||||||||||||||||||||||||||||||||||
Sbjct  19192  GAAGCTGGCGGCAGTCAGGAGATGGTGGAGGGTTTTCGTCAGGCAATTAAAGGCAGCGCC  19251
Query  601    ATCAACATTGTGGATATCGCCTGGGGCGATAACGACATTGAAGTGCAGAGAAACTTACTC  660
              ||||||||||||||||||||||||||||||||||||||||||||||||||||||||||||
Sbjct  19252  ATCAACATTGTGGATATCGCCTGGGGCGATAACGACATTGAAGTGCAGAGAAACTTACTC  19311
Query  661    CAGGAAATGCTGGAGCGTCATCCGGACGCCAATGTGGTCGCCGGGTCGGCGATAGCGGCG  720
              ||||||||||||||||||||||||||||||||||||||||||||||||||||||||||||
Sbjct  19312  CAGGAAATGCTGGAGCGTCATCCGGACGCCAATGTGGTCGCCGGGTCGGCGATAGCGGCG  19371
Query  721    GAGGCGGCGATGGGCGAAGGGCGAAATTTGACGACTCCGCTCACGATCGTCTCATTTTAT  780
```

Supplementary Figure 1 BLAST search results using *Salmonella* Gallinarum *torT* nucleotide sequence (GenBank accession no. AM933173.1 segment 3797861-3798901) against the nucleotide collection (nr/nt) database. The maximum number of aligned sequences to display was set to the maximum value of 5,000, and the other parameters were set to default values. The results showed that a 126 bp-region of deletion in the carboxyl terminal of *torT* was observed only in *S.* Pullorum after comparison with that of *S.* Gallinarum and other *Salmonella* serovars, which could be used for the specific identification of *S.* Pullorum.
