# Supplementary material for: Accurate identification and discrimination of Salmonella enterica serovar Gallinarum biovars Gallinarum and Pullorum by a multiplex PCR based on the new genes of torT and I137_14430
Source: Front Vet Sci. 2023 Jul 5;10:1220118. doi: 10.3389/fvets.2023.1220118 (PMC10354433; doi:10.3389/fvets.2023.1220118)
Supplement: Supplementary file 4 [file Data_Sheet_3.PDF]

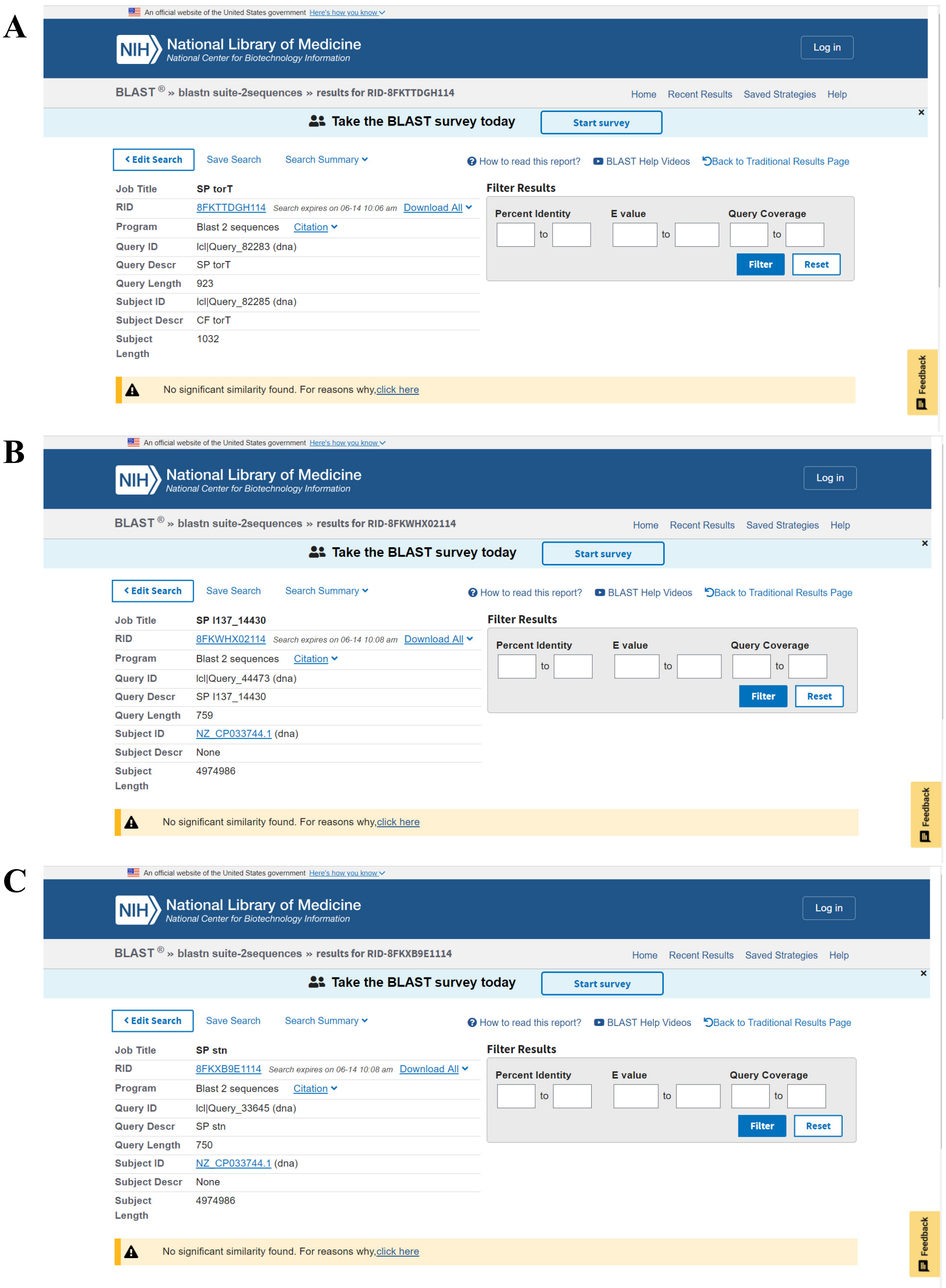

Supplementary Figure 3 BLAST search results using *S.* Pullorum *torT*, *I137_14430* and *stn* nucleotide sequences against the *torT* or genome sequences of *Citrobacter freundii* strain FDAARGOS_549 (GenBank accession no. NZ_CP033744.1). The results show that the length of *S.* Pullorum *torT* gene (CP006575.1) is 923 bp. However, the length of *C. freundii torT* gene is 1032 bp. Besides, no significant similarity was found in the *torT* sequences between the two species (**A**). *I137_14430* and *stn* genes of *S.* Pullorum was aligned with the genome of *C. freundii*, and no significant similarity was found in *C. freundii* genome for both *I137_14430* sequence (**B**) and *stn* sequence (**C**).
