# Supplementary material for: Accurate identification and discrimination of Salmonella enterica serovar Gallinarum biovars Gallinarum and Pullorum by a multiplex PCR based on the new genes of torT and I137_14430
Source: Front Vet Sci. 2023 Jul 5;10:1220118. doi: 10.3389/fvets.2023.1220118 (PMC10354433; doi:10.3389/fvets.2023.1220118)
Supplement: Supplementary file 5 [file Data_Sheet_4.PDF]

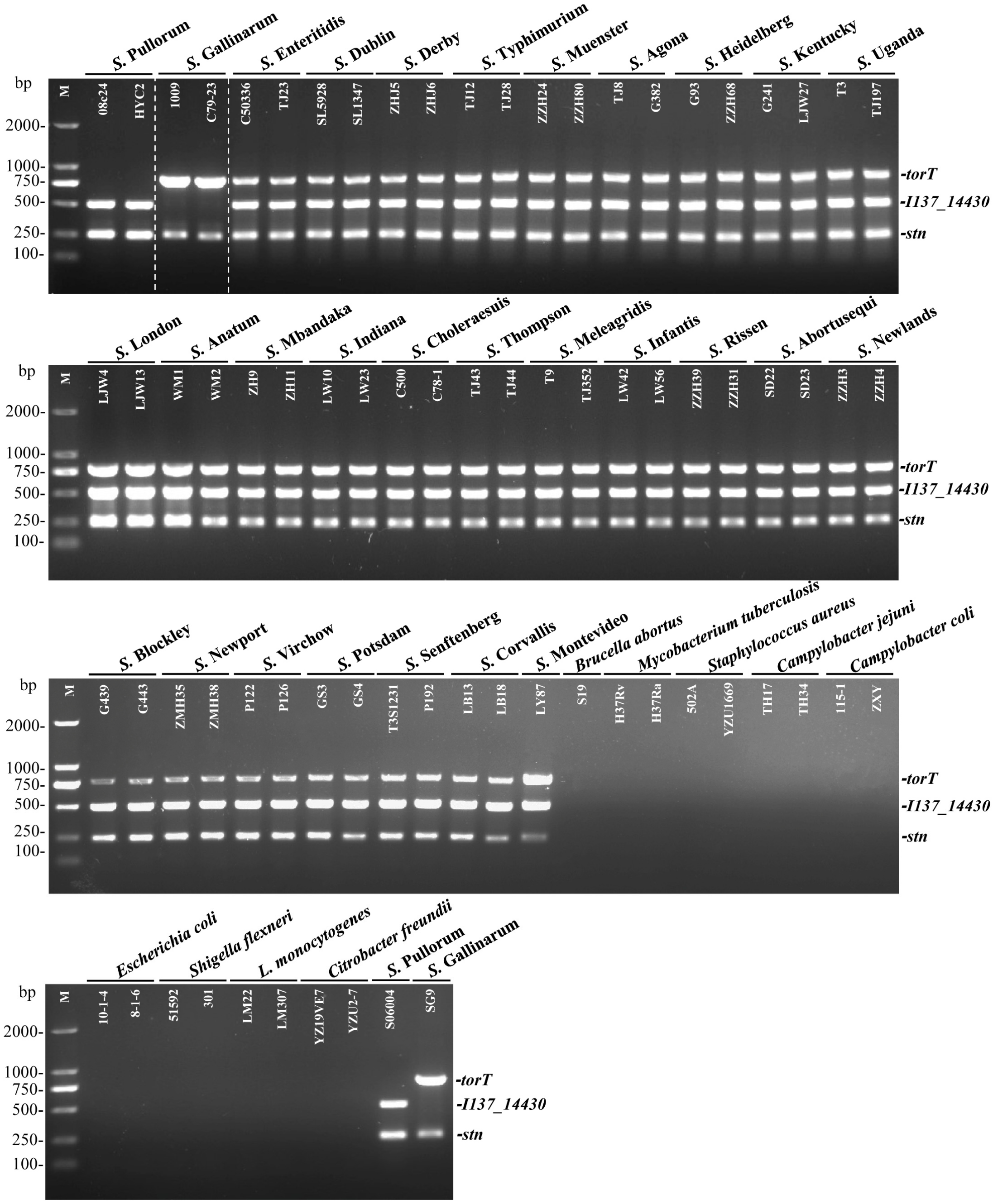

Supplementary Figure 4 Specificity of the one-step multiplex PCR for the detection and differentiation of *S.* Pullorum and *S.* Gallinarum was cross-validated in another laboratory. Two strains were randomly selected for each *Salmonella* serotype and non-*Salmonella* pathogens (including *Citrobacter* spp.). The detailed strains were presented in Supplementary Table S1. The PCR amplifies only two specific products of 508-bp *I137_14430* and 252-bp *stn* for *S.* Pullorum. Only two products of 801-bp *torT* and 252-bp *stn* were generated for *S.* Gallinarum. All three products of *torT*, *I137-14430*, and *stn* were amplified for other *Salmonella* serovars. Nonetheless, there was not a single band generated for any other non-*Salmonella* pathogens. Lane M: DL2000 DNA marker (Takara Biotechnology Co., Dalian, China).
